# Novel Paracrine Action of Endothelium Enhances Glucose Uptake in Muscle and Fat

**DOI:** 10.1161/CIRCRESAHA.121.319517

**Published:** 2021-08-20

**Authors:** Hema Viswambharan, Nadira Y. Yuldasheva, Helen Imrie, Katherine Bridge, Natalie J. Haywood, Anna Skromna, Karen E. Hemmings, Emily R. Clark, V. Kate Gatenby, Paul Cordell, Katie J. Simmons, Natallia Makava, Yilizila Abudushalamu, Naima Endesh, Jane Brown, Andrew M.N. Walker, Simon T. Futers, Karen E. Porter, Richard M. Cubbon, Khalid Naseem, Ajay M. Shah, David J. Beech, Stephen B. Wheatcroft, Mark T. Kearney, Piruthivi Sukumar

**Affiliations:** 1 Leeds Institute for Cardiovascular and Metabolic Medicine, University of Leeds, United Kingdom (H.V., N.Y.Y., H.I., K.B., N.J.H., A.S., K.E.H., E.R.C., V.K.G., P.C., K.J.S., N.M., Y.A., N.E., J.B., A.M.N.W., S.T.F., K.E.P., R.M.C., K.N., D.J.B., S.B.W., M.T.K., P.S.).; 2 British Heart Foundation Centre of Research Excellence, King’s College London (A.M.S.).

**Keywords:** catalase, endothelium, fasting, glucose, insulin, microRNAs, muscles

## Abstract

**Rationale:**

A hallmark of type 2 diabetes is insulin resistance, which leads to increased endothelial cell (EC) production of superoxide and a simultaneous reduction in the availability of the vasoprotective signaling radical NO. We recently demonstrated in preclinical models that type 2 diabetes simultaneously causes resistance to IGF-1 (insulin-like growth factor-1)–mediated glucose lowering and endothelial NO release.

**Objective:**

To examine the effect of insulin and IGF-1 resistance specifically in ECs in vivo.

**Methods and Results:**

We generated mice expressing mIGF-1Rs (mouse IGF-1 receptors), which form nonfunctioning hybrid receptors with native IRs (insulin receptors) and IGF-1R, directed to ECs under control of the *Tie2* promoter-enhancer. Despite EC insulin and IGF-1 resistance, mIGFREO (mutant IGF-1R EC overexpressing) mice had enhanced insulin and IGF-1–mediated systemic glucose disposal, lower fasting free fatty acids, and triglycerides. In hyperinsulinemic-euglycemic clamp studies, mIGFREO had increased glucose disposal and increased glucose uptake into muscle and fat, in response to insulin. mIGFREO had increased Nox (NADPH oxidase)-4 expression due to reduced expression of the microRNA, miR-25. Consistent with increased Nox4, mIGFREO ECs generated increased hydrogen peroxide (H_2_O_2_), with no increase in superoxide. Treatment with catalase—a H_2_O_2_ dismutase—restored insulin tolerance to WT (wild type) levels in mIGFREO.

**Conclusions:**

Combined insulin and IGF-1 resistance restricted to the endothelium leads to a potentially favorable adaptation in contrast to pure insulin resistance, with increased Nox4-derived H_2_O_2_ generation mediating enhanced whole-body insulin sensitivity.

## Abstract 2

Supplemental Digital Content is available in the text.

**In This Issue, see p 699**

**Meet the First Author, see p 700**

A hallmark of type 2 diabetes is insulin resistance, defined as an inability of insulin to activate its complex intracellular signaling network appropriately.^1^ In addition to regulating glucose homeostasis, insulin activates the enzyme eNOS (endothelial NO synthase) in endothelial cells (ECs) to stimulate generation of the signaling radical NO.^2,3^ Acting via its tyrosine kinase receptor, which is structurally homologous to the IR (insulin receptor), IGF-1 (insulin-like growth factor-1) also regulates metabolic and cellular responses to nutrient availability^4^ and at the same time may stimulate eNOS in ECs to generate NO.^5^

We have shown that insulin resistance specific to the endothelium induced by a range of different mechanisms leads to reduced availability of NO and generation of excess concentrations of the free radical superoxide—the principal enzymatic source of which is the Nox (NADPH oxidase) 2 isoform of Nox.^6–9^ However, we have also shown that diet-induced obesity leads to excess generation of the oxidant and dismutation product of superoxide, hydrogen peroxide (H_2_O_2_).^10^ Moreover, we showed that diet-induced obesity leads to resistance to both insulin- and IGF-1–mediated glucose lowering and serine phosphorylation-mediated activation of eNOS.^5^ To improve our understanding of the synergistic impact of insulin and IGF-1 signaling in the endothelium, we generated a transgenic mouse expressing mIGF-1R (mouse IGF-1 receptor), which forms nonfunctioning hybrid receptors with native IRs and IGF-1R specifically in the endothelium (herewith described as mIGFREO [mutant IGF-1R EC overexpressing]). When expressed exclusively in muscle, mIGF-1R induces resistance to both insulin and IGF-1.^11^ Here, we describe for the first time the effect of endothelium-restricted insulin and IGF-1 resistance, secondary to expression of mIGF-1R, on whole-body insulin sensitivity and EC homeostasis.

## Methods

### Data Availability

The authors declare that all supporting data and materials/protocols presented within this article and in the Data Supplement are available from the corresponding author by reasonable request. Materials and Methods are described in detail, in the Data Supplement. Please see the Major Resources Table in the Data Supplement.

## Results

ECs from patients with type 2 diabetes are resistant to both insulin- and IGF-1–mediated eNOS phosphorylation.

**Figure 1.**
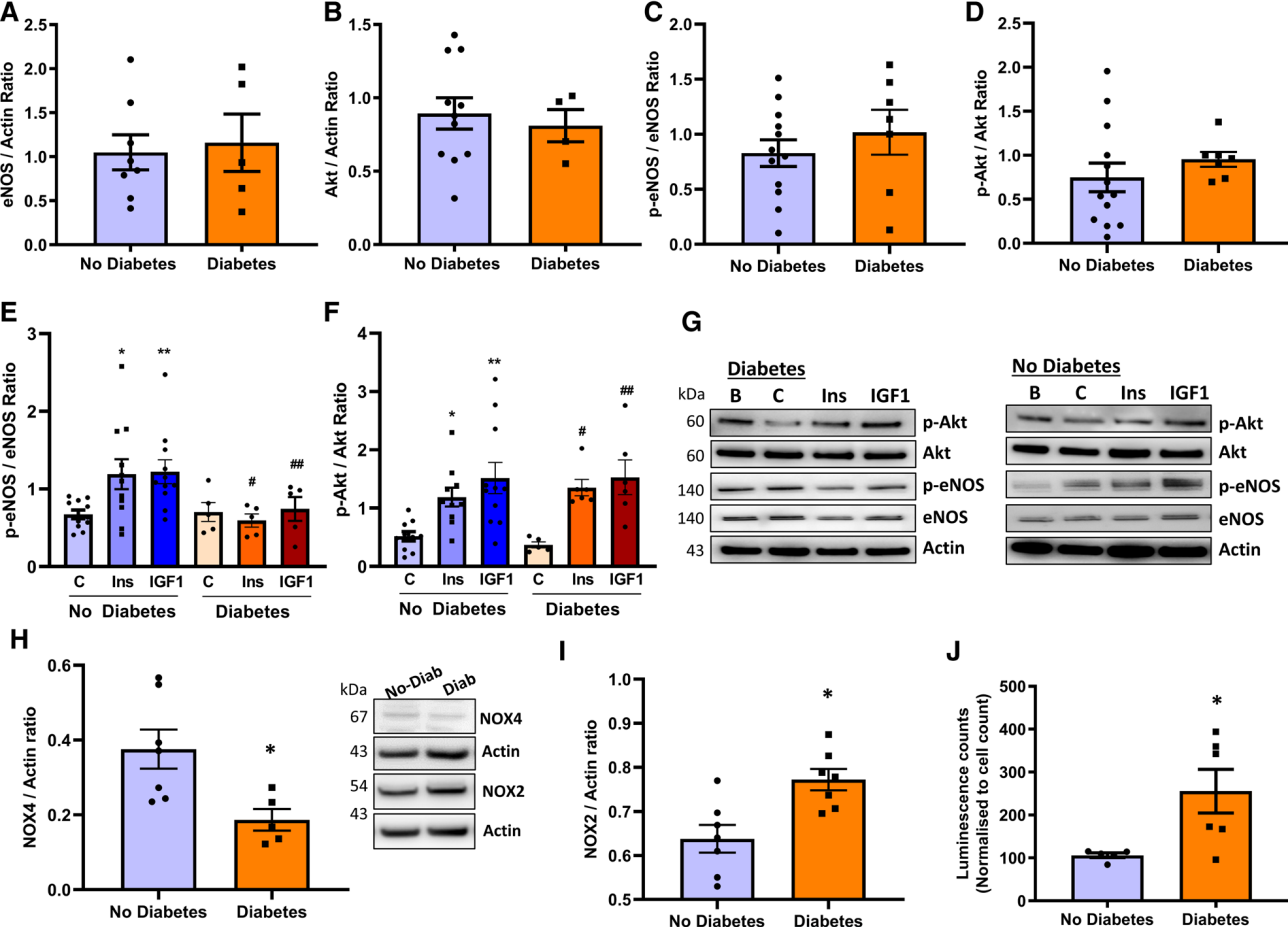
**Endothelial cells from humans with type 2 diabetes and advanced atherosclerosis are resistant to both insulin (Ins) and IGF-1.A** and **B**, Unstimulated, basal total eNOS (endothelial NO synthase; **A**; no diabetes, n=5; diabetes, n=8) and AKT (protein kinase B) (**B**; no diabetes, n=12; diabetes, n=5) expression in saphenous vein endothelial cells (SVECs) from patients with and without type 2 diabetes. **C** and **D**, Serine phosphorylated eNOS (**C**; no diabetes, n=12; diabetes, n=7) and serine phosphorylated Akt (**D**; no diabetes, n=13; diabetes, n=7) in SVEC under basal condition. **E**, Ins or IGF-1 stimulated eNOS serine phosphorylation (100 nM, 10 min) in SVEC from patients with diabetes (no diabetes, n=12; diabetes, n=7). **F**, Akt serine phosphorylation upon Ins (100 nM, 10 min) and IGF-1 (100 nM, 10 min) treatment in SVEC from patients with and without diabetes (no diabetes, n=11; diabetes, n=6). **G**, Representative blots for the data shown in **E** and **F**. **H** and **I**, Nox4 (**H**; no diabetes, n=7; diabetes, n=5) and Nox2 (**I**; no diabetes, n=7; diabetes, n=7) expression in SVEC from patients with (Diab) or without diabetes (No-Diab; representative blots presented on the right of **H**). **J**, Mean superoxide level in SVEC from patients with and without diabetes (no diabetes, n=5; diabetes, n=6). All SVECs used after isolation were at passages 2 to 5. Data expressed as mean±SEM. *P<0.05, no diabetes vs diabetes in **H–J**. **E**, *P<0.05, control vs Ins stimulation in patients without diabetes; **P<0.05, control vs IGF-1 stimulation in patients without diabetes; #*P*<0.05, Ins stimulation in diabetes vs without diabetes; ##*P*<0.05, IGF-1 stimulation in diabetes vs without diabetes. **F**, *P<0.05, control vs Ins stimulation in patients without diabetes; **P<0.05, control vs IGF-1 stimulation in patients without diabetes, #*P*<0.05, control vs Ins stimulation in diabetes; ##*P*<0.05, control vs IGF-1 stimulation in diabetes (**B** denotes basal unstimulated cells grown in full growth medium for total protein expression. **C** denotes control cells in 0.2% serum-containing medium. Data in **A–D** and **H–J** were analyzed using the unpaired Student *t* test. Others were analyzed using 1-way ANOVA Fisher test.

Patients with and without diabetes undergoing coronary bypass surgery were recruited. Total and serine phosphorylated eNOS and Akt (protein kinase B) in saphenous vein ECs (SVECs) were quantified using the Western blot under basal conditions and after stimulation with insulin or IGF-1. Basal eNOS and AKT was similar in SVEC from patients with and without type 2 diabetes (Figure 1A and 1B). Basal serine phosphorylated eNOS was similar in SVEC from patients with or without type 2 diabetes, as was basal serine phosphorylated Akt (Figure 1C and 1D). As we demonstrated in preclinical models,^5^ insulin and IGF-1 stimulated eNOS phosphorylation were blunted in SVEC from patients experiencing type 2 diabetes, compared with patients without type 2 diabetes (Figure 1E and 1G). Insulin and IGF-1 stimulated Akt phosphorylation were similar when comparing SVEC from patients with and without type 2 diabetes (Figure 1F and 1G). There was lower Nox4 expression in SVEC from patients with type 2 diabetes compared with patients without type 2 diabetes, whereas Nox2 protein levels were increased in SVEC from patients with type 2 diabetes compared with patients without diabetes (Figure 1H and 1I). Superoxide generation was higher in SVEC from patients with diabetes (Figure 1J).

### Generation and Characterization of Transgenic Mice With Endothelial Specific Expression of Mutant IGF-1 Receptors

**Figure 2.**
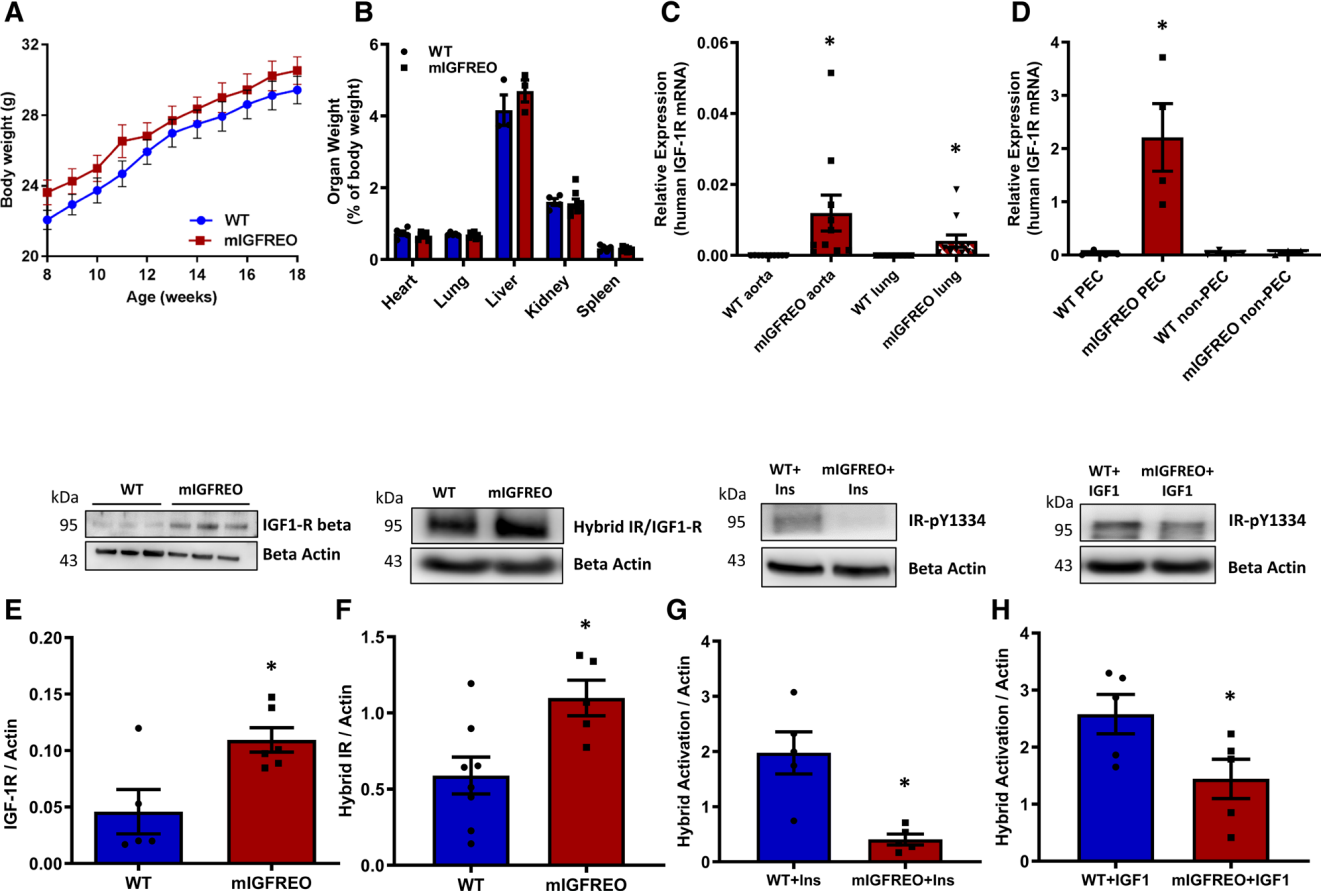
**Generation and characterization of transgenic mice overexpressing mutant IGF-1 receptor in the endothelium.A**, Body (n=11 each group) and (**B**) organ weights of mIGFREO (mutant IGF-1R EC overexpressing) and WT (wild type) littermate mice (WT, n=5; mIGFREO, n=8). **C** and **D**, Mutant human IGF-1R mRNA expression in aorta and lungs (**C**; WT aorta and mIGFREO aorta, n=10; WT lungs, n=9; mIGFREO lungs, n=11) in mIGFREO mice and in pulmonary endothelial cells (PECs) and nonendothelial cells (non-PEC, described in Methods; **D**; WT, n=4; mIGFREO, n=4). **E** and **F**, IGF-1 receptor (IGF-1R) expression (**E**; WT, n=5; mIGFREO, n=6) and hybrid receptor (**F**; WT, n=8; mIGFREO, n=5) protein expression in PEC of mIGFREO and WT. **G** and **H**, Hybrid receptor activation at insulin receptor IR (insulin receptor)-pY1334, upon insulin (Ins; 150 nM, 10 min; **G**; WT, n=5; mIGFREO, n=5) and IGF-1 stimulation (IGF-1; 150 nM, 10 min; **H**; WT, n=5; mIGFREO, n=6) in PEC of mIGFREO and WT. Data expressed as mean±SEM. *P<0.05. C–H, *P<0.05, WT vs mIGFREO. Data in **A** were analyzed using 2-way ANOVA, followed by Bonferroni multiple comparisons test. Data in **B** were analyzed by multiple *t* test. Data in **C–E** were analyzed by unpaired Student *t* test with Mann-Whitney comparison. Data in **F–H** were analyzed by unpaired Student *t* test.

To examine the effect of disrupting both insulin and IGF-1 signaling in the endothelium, we generated a novel transgenic mouse expressing a hIGF-1R (human IGF-1R) with an amino acid substitution in the ATPase domain^11^ directed to the endothelium under control of the *Tie2* promoter-enhancer (Figure IA through IC in the Data Supplement). Mutant IGF-1R endothelium overexpressing mice (described as mIGFREO) were born with the same frequency as their WT (wild type) littermates (data not shown). There was no difference in body weight, organ weight, fat pad size, or blood pressure between mIGFREO and their WT littermates (Figure 2A and 2B; Figure ID and IE in the Data Supplement). We next quantified the levels of human and native (mouse) IGF-1R mRNA expression in whole organs and pulmonary ECs (PECs). While hIGF-1R mRNA was detected only in mIGFREO aorta and lungs (Figure 2C), native (mIGF-1R) mRNA was similar in both mIGFREO and WT aorta and lung (Figure IF in the Data Supplement). There was no hIGF-1R mRNA detectable in PEC from WT mice or non-ECs from mIGFREO (Figure 2D), and there was almost no detectable expression of hIGF-1R in monocytes from mIGFREO (Figure IG in the Data Supplement). hIGF-1R mRNA was not detected in pancreatic islets, pericytes, or endothelium denuded aorta of mIGFREO mouse showing the specificity of the Tie-2 promoter in mIGFREO (Figure IH in the Data Supplement). Western blot analysis confirmed that total IGF-1R protein levels were significantly increased in mIGFREO ECs compared with WT littermates, as were IR/IGF-1R heterodimers (hybrid receptors; Figure 2E and 2F). Hybrid receptors in mIGFREO cells were resistant to insulin, as expected,^12^ IGF-1 stimulated activation of hybrids was also significantly reduced in mIGFREO (Figure 2G and 2H).

### Disrupted Insulin and IGF-1 Signaling in mIGFREO ECs

**Figure 3.**
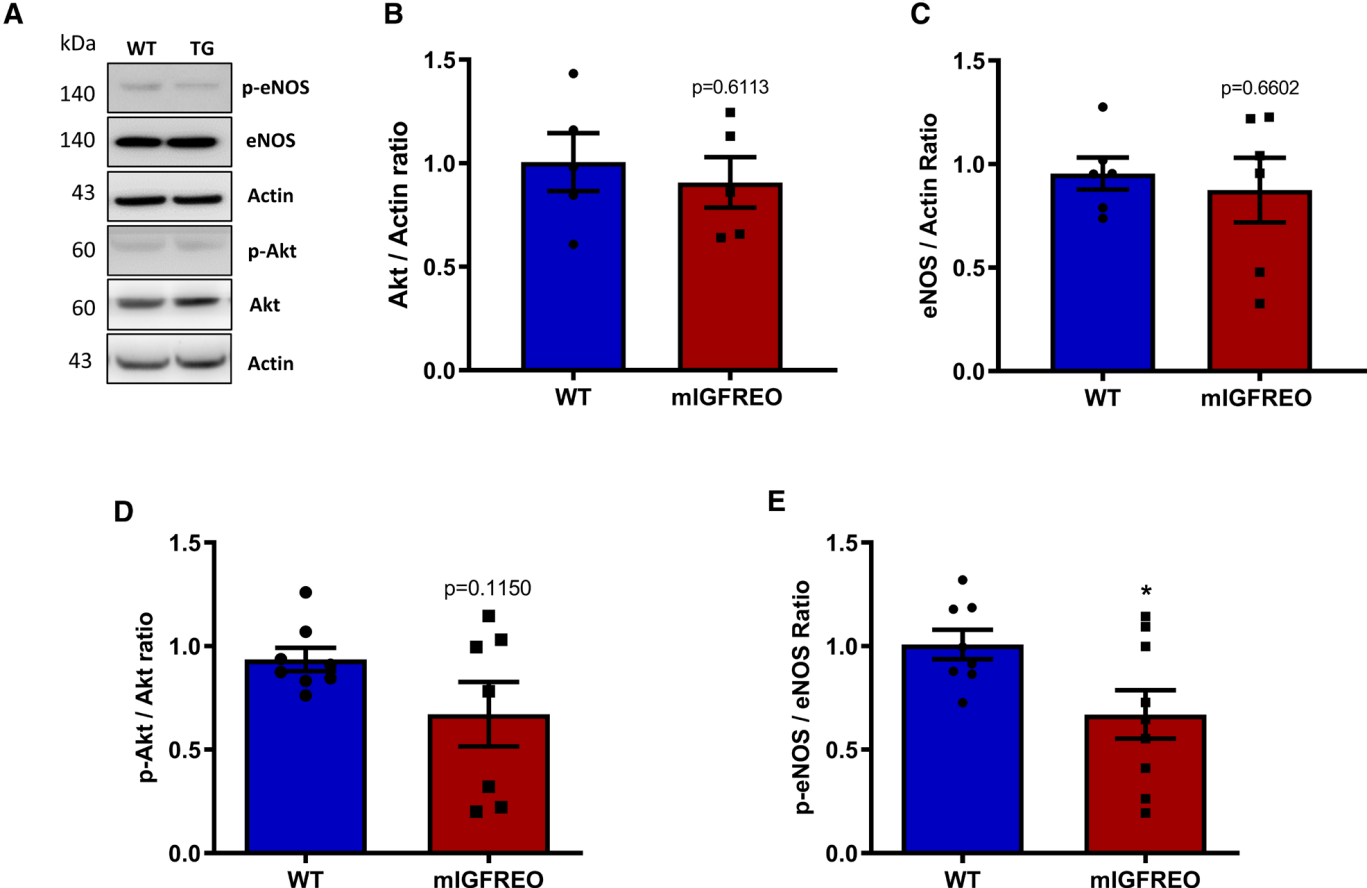
**Disrupted insulin and IGF-1 signaling in mIGFREO (mutant IGF-1R EC overexpressing) pulmonary endothelial cells (PECs).A**, Representative blots for B–E. **B** and **C**, Total Akt (protein kinase B) (**B**; WT [wild type], n=5; mIGFREO, n=5) and total eNOS (endothelial NO synthase; **C**; WT, n=6; mIGFREO, n=6) expression in WT and mIGFREO PEC. **D** and **E**, Basal serine phosphorylated AKT (**D**; WT, n=8; mIGFREO, n=7) and serine phosphorylated eNOS (**E**; WT, n=8; mIGFREO, n=9) expressions in WT and mIGFREO PEC. Data expressed as mean±SEM. *P<0.05, WT vs mIGFREO. Data in **B–E** were analyzed using the unpaired Student *t* test.

**Figure 4.**
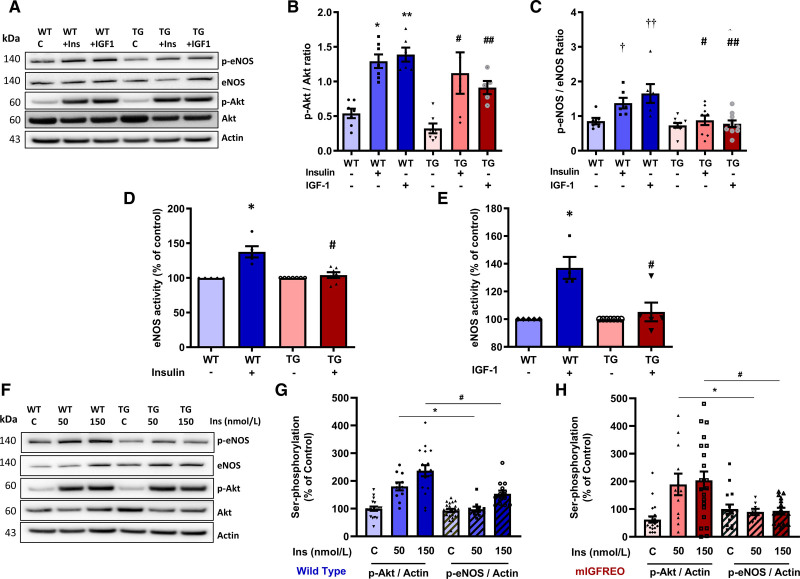
**mIGFREO (mutant IGF-1R EC overexpressing) mice had blunted eNOS (endothelial NO synthase) phosphorylation while sustained Akt (protein kinase B) phosphorylation in response to insulin or IGF-1.A**, Representative blots for B and C. **B** and **C**, Insulin (Ins; 150 nM, 10 min) and IGF-1 (IGF-1; 150 nM, 10 min) stimulated Akt (**B**; WT [wild type], n=7; mIGFREO, n=7), eNOS (**C**; WT, n=6; mIGFREO, n=9) serine phosphorylation in WT and mIGFREO pulmonary endothelial cell (PEC). **D** and **E**, eNOS activity in WT and mIGFREO (TG) PEC upon insulin (150 nM, 30 min; **D**; WT, n=5; mIGFREO, n=7) and IGF-1 (150 nM, 30 min; **E**; WT, n=5; mIGFREO, n=7) stimulation. **F**, Representative blots for G and H. **G** and **H**, Dose-dependent response to insulin (Ins; 50, 150 nM, 10 min) induced serine phosphorylation of Akt and eNOS in WT endothelial cells (PEC; **G**; WT, n=21; mIGFREO, n=22). Dose-dependent response to insulin (Ins; 50, 150 nM, 10 min) induced serine phosphorylation of Akt and eNOS in mIGFREO endothelial cells (**H**; WT, n=21; mIGFREO, n=22). mIGFREO/TG denotes mutant human IGF1-R transgenic mice. Data expressed as mean±SEM (**B**: *P<0.05, control vs insulin-stimulated Akt phosphorylation; **P<0.05, control vs IGF-1–stimulated Akt phosphorylation in WT littermates; #*P*<0.05, control vs insulin-stimulated Akt phosphorylation; ##*P*<0.05, control vs IGF-1–stimulated Akt phosphorylation in mIGFREO cells. **C**: †*P*<0.05, control vs insulin-stimulated eNOS phosphorylation; ††*P*<0.05, control vs IGF-1–stimulated eNOS phosphorylation in WT cells; #*P*<0.05, WT+insulin vs TG+insulin for eNOS phosphorylation; ##*P*<0.05, WT+IGF-1 vs TG+IGF-1 for eNOS phosphorylation. **D** and **E**: *P<0.05, WT control vs WT+insulin or WT control vs WT+IGF-1; #*P*<0.05, WT+insulin vs TG+insulin or WT+IGF-1 vs TG+IGF-1. **G** and **H**: *P<0.05, serine phosphorylation of Akt or eNOS at 50 nmol/L insulin in WT or mIGFREO; #*P*<0.05 serine phosphorylation of Akt or eNOS at 150 nmol/L insulin in WT or mIGFREO). Data in **B–H** were analyzed using 1-way ANOVA, followed by Fisher test.

There was no difference in total eNOS, total Akt, or phosphorylated Akt during standard culture conditions in mIGFREO PEC compared with WT littermates (Figure 3A through 3D). Serine phosphorylated eNOS was significantly reduced in mIGFREO ECs compared with WT littermates (Figure 3E). Basal and insulin-induced phosphorylation at Tyr657—an important residue in the negative regulation of eNOS—was not significantly different between WT and mIGREO PEC (Figure IIA in the Data Supplement). While, as seen in humans with type 2 diabetes, mIGFREO PEC had reduced serine phosphorylation of eNOS in response to insulin and IGF-1, insulin-stimulated Akt phosphorylation was preserved (Figure 4A through 4C). Radioactive eNOS activity assay and phosphorylation of eNOS analysis of whole aorta also showed that mIGFREO ECs were resistant to insulin and IGF-1 stimulation (Figure 4D and 4E; Figure IIB in the Data Supplement). We, therefore, examined the possibility that eNOS and Akt demonstrate differential sensitivities to insulin-mediated serine phosphorylation in ECs. We observed that in WT PEC, while AKT phosphorylation was induced on exposure to 50 nmol/L insulin, significant eNOS phosphorylation required a substantially higher concentration (150 nmol/L; Figure 4F and 4G). Similarly in insulin-resistant PEC from mIGFREO mice, although AKT phosphorylation was induced with 50 nmol/L insulin, eNOS was relatively resistant to phosphorylation at 150 nmol/L (Figure 4H). Next, we probed ERK (MAP kinase 1/2) and PKC (protein kinase C)—two key molecules involved in insulin signaling in ECs. While we did not observe any difference in insulin-induced ERK phosphorylation between WT and mIGFREO PEC, PKC activity in response to insulin stimulation was reduced in mIGFREO PEC compared with WT cells (Figure IIC and IID in the Data Supplement).

### mIGFREO Mice Have Normal Glucose Tolerance but Enhanced Glucose Lowering in Response to Systemic Insulin or IGF-1

**Figure 5.**
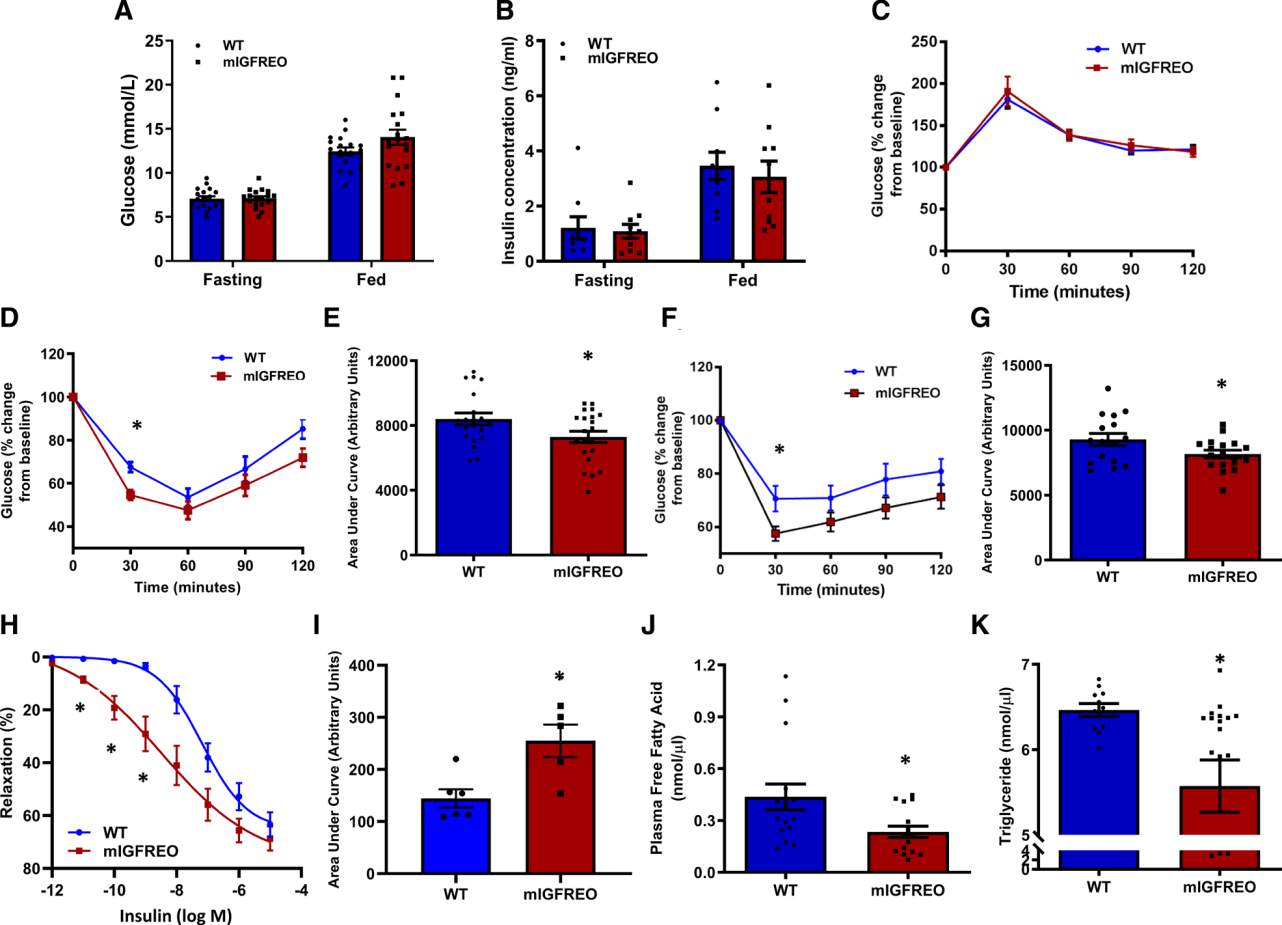
**mIGFREO (mutant IGF-1R EC overexpressing) mice have normal glucose tolerance but enhanced glucose lowering in response to systemic insulin or IGF-1.A** and **B**, Blood glucose (**A**; WT [wild type], n=18; mIGFREO, n=18) and insulin concentration (**B**; WT, n=5; mIGFREO, n=7) in mIGFREO and WT littermates in fasting and fed state. **C–G**, Glucose tolerance (**C**; WT, n=20; mIGFREO, n=22), insulin tolerance (**D**; WT, n=20; mIGFREO, n=22), area under the curve for insulin tolerance tests (**E**; WT, n=20; mIGFREO, n=21), IGF-1 tolerance (**F**; WT, n=16; mIGFREO, n=17), and area under the curve for IGF-1 tolerance tests (**G**; WT, n=16; mIGFREO, n=17) in mIGFREO mice and WT littermates. **H**, Insulin-induced vasorelaxation in second-order mesenteric vessels from WT (n=17) and mIGFREO (n=20). **I**, Mean area under the curve of the vasorelaxation data presented in **H**. **J** and **K**, Plasma-free fatty acid (**J**; WT, n=16; mIGFREO, n=16) and triglyceride concentration in mIGFREO and WT littermates (**K**; WT, n=11; mIGFREO, n=18). Data expressed as mean±SEM. *P<0.05, WT vs mIGFREO. Data in **A**, **B**, **E**, **G**, and **I** were analyzed using unpaired Student *t* test. Data in **C**, **D**, and **F** were analyzed using 2-way ANOVA, followed by Fisher test. Data in **H** were analyzed using two-way ANOVA, followed by Bonferroni multiple comparisons test. Data in **J** and **K** were analyzed using unpaired *t* test, followed by Mann-Whitney *U* test.

**Figure 6.**
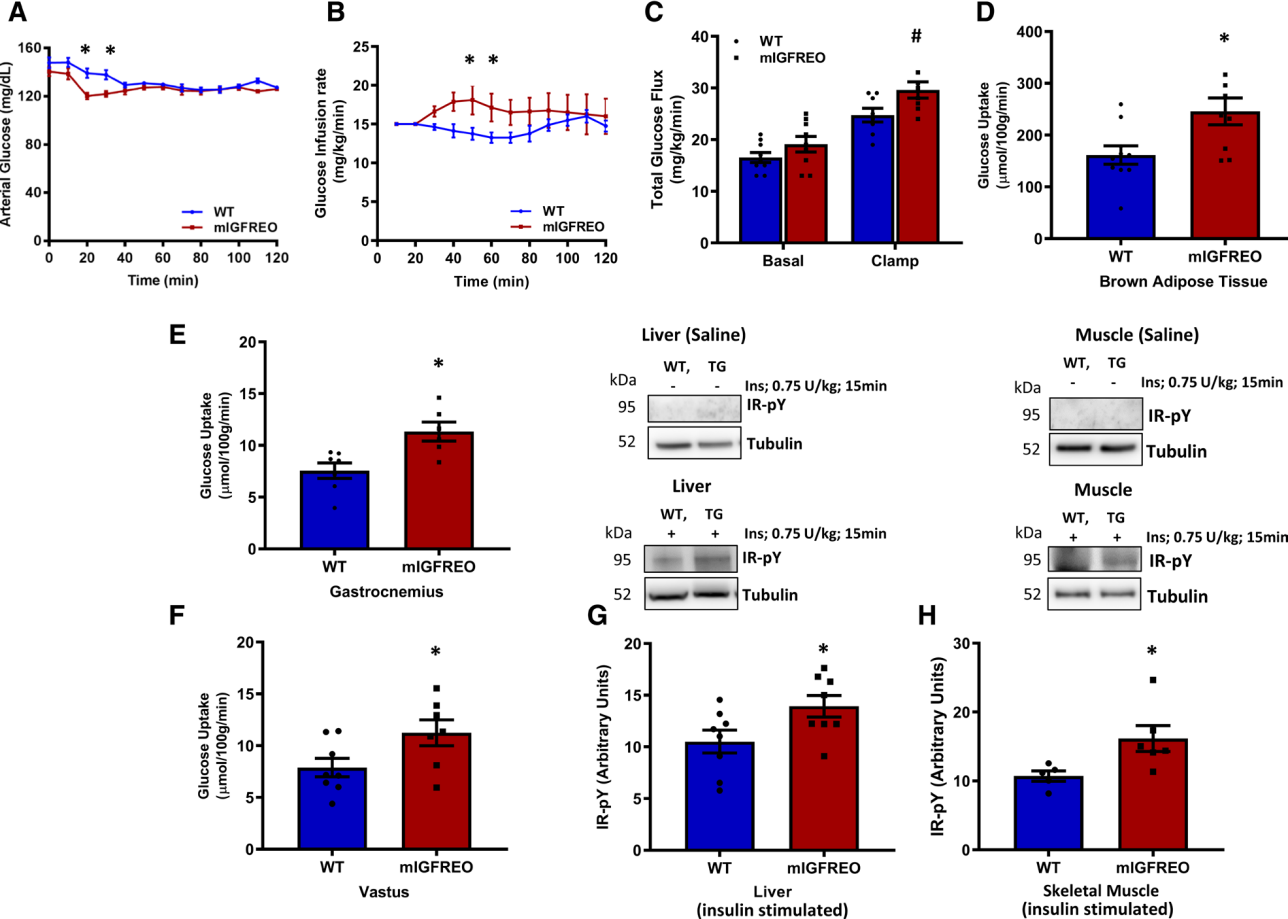
**Enhanced insulin sensitivity in mIGFREO (mutant IGF-1R EC overexpressing) mice.A–C**, Data from low-dose hyperinsulinemic-euglycemic clamp studies on mIGFREO and WT (wild type) littermates showing blood glucose (**A**), glucose infusion rate (**B**), and rate of glucose disappearance (Rd; **C**) during hyperinsulinemic-euglycemic clamp (WT, n=8; mIGFREO, n=8). **D–F**, Tissue-specific glucose uptake into brown adipose tissue (**D**; WT, n=10; mIGFREO, n=9), gastrocnemius skeletal muscle (**E**; WT, n=7; mIGFREO, n=6), and vastus skeletal muscle (**F**; WT, n=8; mIGFREO, n=7). **G** and **H**, Insulin (intraperitoneal injection; 0.75 U/kg, 15 min) stimulated tyrosine phosphorylation of IR (insulin receptor; pY-IR) in liver (**G**; WT, n=8; mIGFREO, n=8) and skeletal muscle (**H**; WT, n=5; mIGFREO, n=6). Data expressed as mean±SEM. **A** and **B**, #*P*<0.05, total glucose flux WT vs mIGFREO mice. **D–H**, *P<0.05 WT vs mIGFREO. Data in **A** and **B** were analyzed using 2-way ANOVA, followed by Bonferroni multiple comparisons test. All other data were analyzed by unpaired Student *t* test.

mIGFREO had similar fasting and fed capillary blood glucose, fasting and fed serum insulin, and random serum IGF-1 concentrations to WT littermates (Figure 5A and 5B; Figure IIIA in the Data Supplement). mIGFREO also had similar glucose tolerance as WT littermates but had enhanced glucose disposal in insulin and IGF-1 tolerance tests compared with WT littermates (Figure 5C through 5G). It has been suggested that by increasing delivery of glucose to its target tissues, insulin-induced vasodilation of small arteries is important for glucose uptake.^13^ In light of the enhanced insulin sensitivity at a whole-body level seen in mIGFREO, we examined insulin-induced relaxation in second-order mesenteric arteries. Consistent with the increased insulin-mediated glucose uptake seen in a range of tissues, we observed an increase in insulin-induced vasorelaxation of second-order mesenteric arteries from mIGFREO (Figure 5H and 5I; Figure IIIB in the Data Supplement). Consistent with enhanced insulin sensitivity, fasting free fatty acids and triglycerides were significantly lower in mIGFREO compared with WT littermates (Figure 5J and 5K). Serum concentrations of the adipokines leptin and adiponectin were not different between mIGFREO and WT controls (Figure IIIC and IIID in the Data Supplement). In low-dose hyperinsulinemic-euglycemic clamp studies, mIGFREO blood glucose was maintained throughout the clamp, glucose infusion rate was higher in mIGFREO, consistent with increased insulin sensitivity (Figure 6A and 6B). Fasting (basal) glucose turnover was no different between groups (Figure IVA in the Data Supplement). However, consistent with enhanced insulin sensitivity, the rate of glucose disappearance was significantly higher in mIGFREO (Figure 6C). Endogenous glucose production was no different between groups, indicative of no difference in hepatic gluconeogenesis (Figure IVB in the Data Supplement). In tracer studies, glucose uptake into brown adipose tissue, as well as skeletal muscle, was significantly increased in mIGFREO (Figure 6D through 6F; Figure IVC in the Data Supplement). Consistent with increased insulin sensitivity, in vivo insulin stimulation led to greater tyrosine phosphorylation of IR in liver and skeletal muscle in mIGFREO compared with WT littermates (Figure 6G and 6H).

### Increased Vascular H_2_O_2_ in mIGFREO

Aortic rings from mIGFREO mice had similar responses to the endothelium- and NO-dependent vasodilator acetylcholine (Figure VA in the Data Supplement) and the endothelium-independent vasodilator sodium nitroprusside, compared with WT littermates (Figure VIA in the Data Supplement). Vasoconstriction to phenylephrine remained unchanged in mIGFREO compared with their WT littermates (Figure VB in the Data Supplement). Bioavailable NO in response to isometric tension, assessed by measuring the constrictor response to the NOS inhibitor L-NG-monomethyl arginine citrate, was no different in mIGFREO compared with WT littermates (Figure VC in the Data Supplement). To examine the possibility that H_2_O_2_ generation may contribute to the vasorelaxation response to acetylcholine in mIGFREO, as we previously demonstrated in obese mice,^10^ we treated rings with the H_2_O_2_ dismutase, catalase (Figure VD and VE in the Data Supplement). There was no difference in log-EC_50_ (log-half maximal effective concentration or log-EC50) of acetylcholine responses between mIGFREO and WT littermates after catalase treatment (note logarithmic scale; Figure VF in the Data Supplement). However, percentage change in maximum relaxation was significantly increased in mIGFREO aorta compared with WT littermates (Figure VG in the Data Supplement) demonstrating increased hydrogen peroxide release in acetylcholine-induced vasorelaxation in mIGFREO mice. Next, to test whether inhibiting catalase can reverse catalase-induced reduction of acetylcholine-induced relaxations in aorta, we performed vasorelaxation studies with the catalase inhibitor (3-amino-1,2,4-triazole) in the presence of catalase. The data confirmed the significance of catalase inhibitable H_2_O_2_ in acetylcholine-induced relaxation in mIGFREO aorta (Figure VH in the Data Supplement). To confirm increased H_2_O_2_ production in mIGFREO aortic rings, we examined the amount of catalase-inhibited aortic H_2_O_2_ production using the Amplex Red assay. mIGFREO aorta had significantly higher concentrations of H_2_O_2_ compared with WT littermates (Figure VI in the Data Supplement). We quantified superoxide generation in ECs from mIGFREO using NADPH-dependent chemiluminescence and found no difference in superoxide generation between mIGFREO and WT littermates (Figure VJ in the Data Supplement).

### Excessive Endothelial H_2_O_2_ Leads to Enhanced Whole-Body Insulin Sensitivity

**Figure 7.**
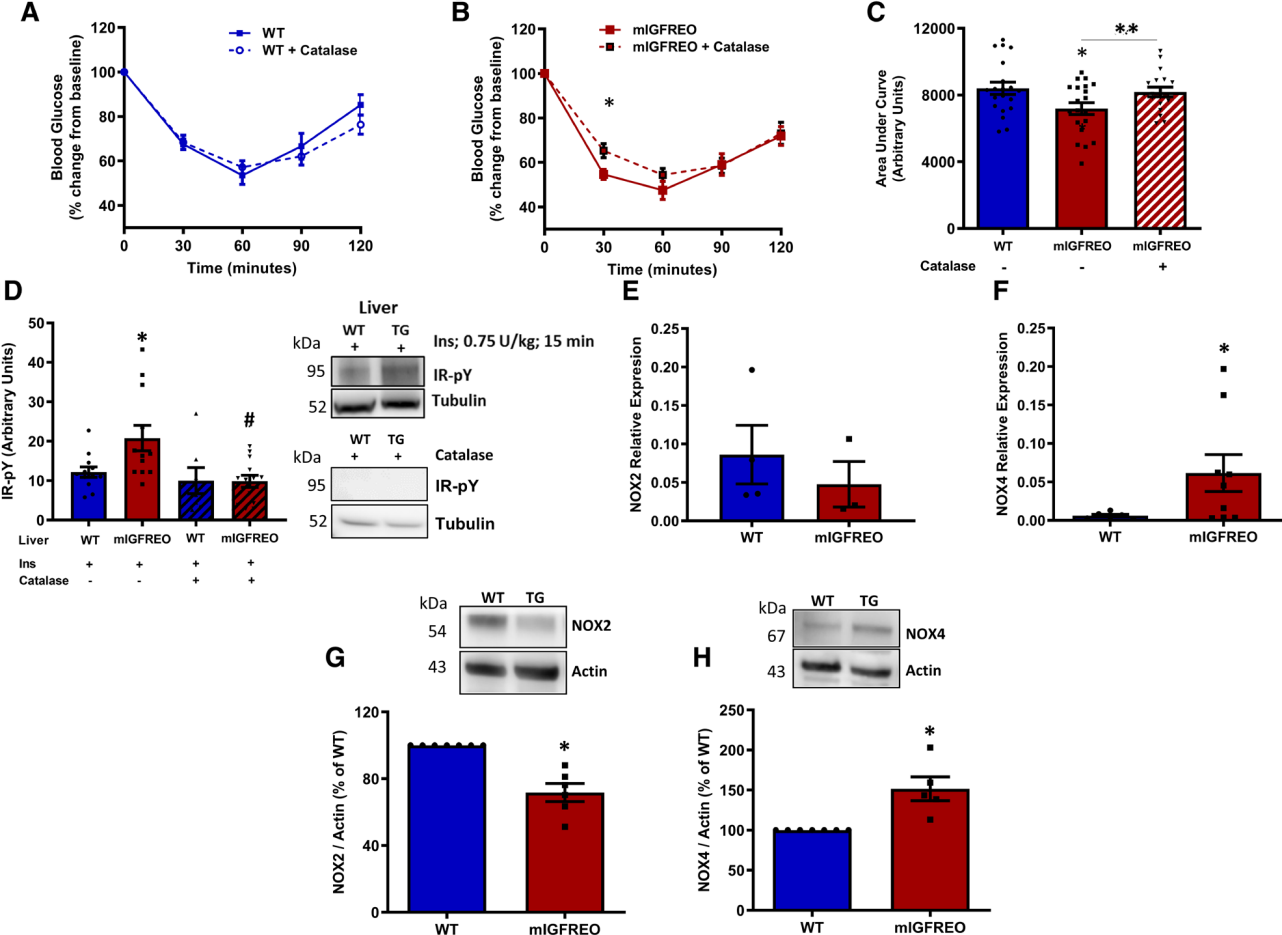
**Excess generation of hydrogen peroxide in mIGFREO (mutant IGF-1R EC overexpressing) mice.A** and **B**, Insulin tolerance testing after catalase infusion in WT (wild type; **A**) and mIGFREO (**B**) mice (WT, n=17; mIGFREO, n=20). **C**, Area under the curve for ITT presented in **A** and **B** showing mead data for WT, mIGFREO, and mIGFREO postcatalase infusion (10 000 U/kg per day; 5 d; WT, n=18; mIGFREO, n=21; **D**; WT, n=12; mIGFREO, n=12) Tyrosine phosphorylation of IR (insulin receptor) upon insulin stimulation (Ins; 0.75 U/kg, 15 min) with and without catalase infusion in liver (n=8; representative blots shown on the right). **E** and **F**, Relative mRNA expression of Nox2 (**E**; WT, n=3; mIGFREO, n=4) and Nox4 (**F**; WT, n=6; mIGFREO, n=9) in endothelial cells of mIGFREO and WT littermates. **G** and **H**, Protein expression of Nox2 (**G**; WT, n=7; mIGFREO, n=6) and Nox4 (**H**; WT, n=7; mIGFREO, n=5) in endothelial cells of mIGFREO and WT littermates. Data expressed as mean±SEM. **B**, *P<0.05 mIGFREO vs mIGFREO+catalase. **C**, **P<0.05 mIGFREO vs mIGFREO+catalase. **D**, *P<0.05, WT vs mIGFREO; #*P*<0.05 mIGFREO vs mIGFREO+catalase. **F–H**, *P<0.05 WT vs mIGFREO. Data in **A** and **B** were analyzed using multiple *t* test. Data in **C** and **D** were analyzed by 1-way ANOVA with Fisher test. Data in **E** were analyzed using unpaired Student *t* test. All others were analyzed using unpaired Student *t* test and Mann-Whitney *U* test.

H_2_O_2_ has previously been described as an insulin sensitizer in skeletal muscle, and we too found that it can increase glucose uptake in muscle cells in culture in a dose-dependent manner (data not shown). Hence, we examined the effect of H_2_O_2_ quenching on insulin sensitivity in vivo in mIGFREO mice. Chronic treatment with catalase restored insulin sensitivity in mIGFREO to WT levels in insulin tolerance testing (Figure 7A through 7C). Moreover, catalase treatment reduced levels of tyrosine phosphorylated IR in mIGFREO liver (Figure 7D). Nox4 has been described as the primary source of H_2_O_2_ in ECs. On the contrary, the related Nox isoform Nox2 is predominately responsible for superoxide generation. Nox2 mRNA expression in ECs from mIGFREO was similar to WT littermates, whereas Nox4 mRNA was significantly higher in mIGFREO (Figure 7E and 7F). EC Nox2 protein expression was lower in mIGFREO compared with WT littermates, whereas and consistent with mRNA expression, Nox4 protein expression was higher in mIGFREO compared with WT littermates (Figure 7G and 7H). No other reactive oxygen species (ROS)-related gene expression was changed in mIGFREO PEC (Figure VIB in the Data Supplement). To dissect how endothelial insulin and IGF-1 resistance contributes to whole-body insulin sensitivity in the pathological context of diet-induced insulin resistance, we fed mIGFREO and WT littermates a high-fat diet (HFD) for 10 days. We have previously shown that short-term HFD feeding can result in resistance to insulin-induced glucose lowering before it causes insulin resistance in the endothelium.^10,12^ There was no significant difference in changes in body weight before and after HFD (Figure VIIA in the Data Supplement) or fasting glucose levels (Figure VIIB in the Data Supplement) between mIGFREO and WT animals upon HFD feeding. mIGFREO mice fed HFD, despite insulin resistance at the level of the endothelium, did not develop glucose intolerance compared with WT littermates (Figure VIIC and VIIE in the Data Supplement). However, detailed exploratory glucose tolerance tests (GTT) analysis at 30 minutes showed that glucose levels trended to be lower in mIGFREO (*P*=0.05) compared with WT littermates (Figure VIIC and VIID in the Data Supplement).

### Mutant IGF-1R Regulates Nox4 Expression Through miR-25 in ECs From mIGFREO and Patients With Type 2 Diabetes

**Figure 8.**
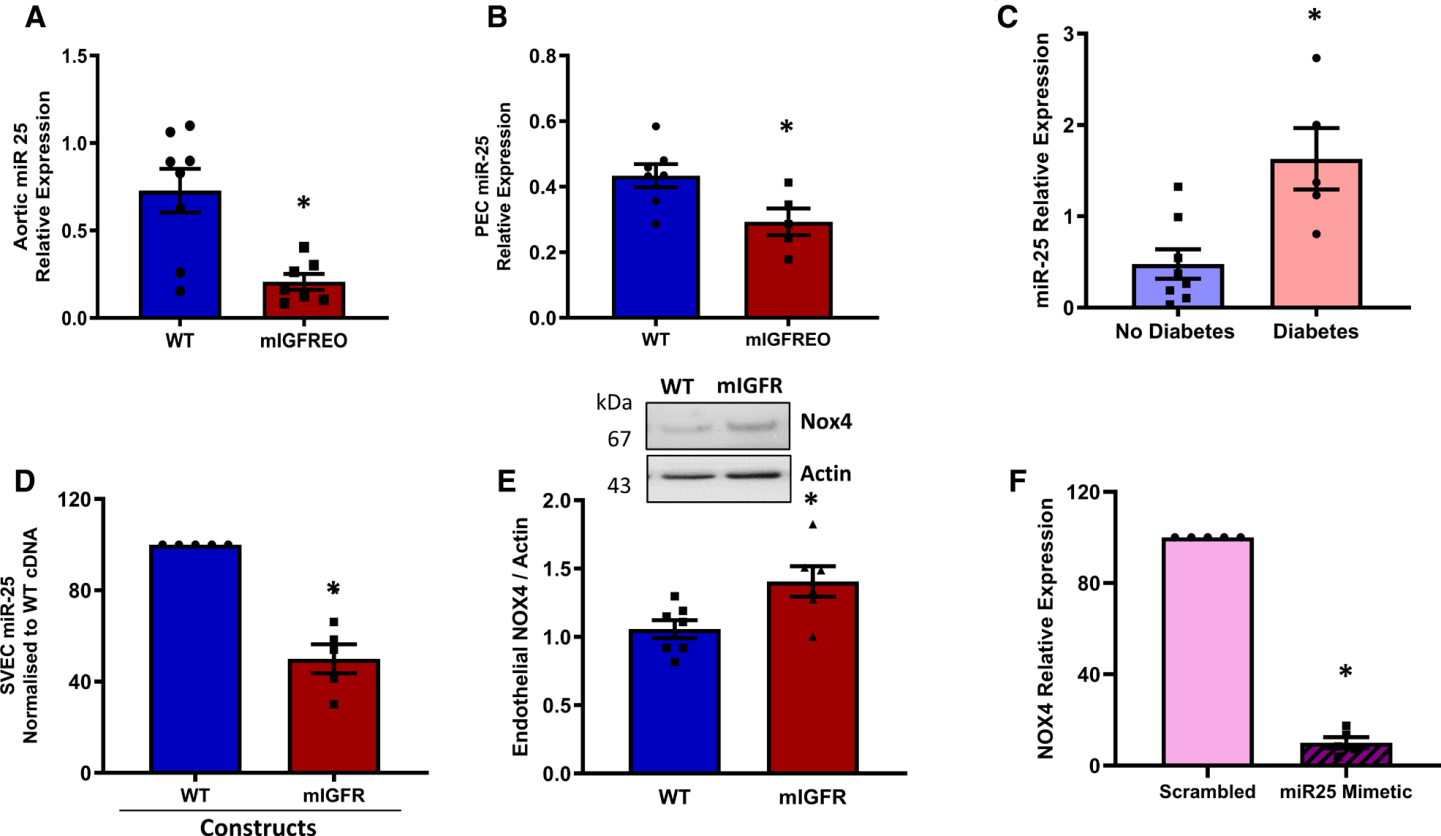
**miR-25 levels in mIGFREO (mutant IGF-1R EC overexpressing) and saphenous vein endothelial cells (SVECs).A** and **B**, Aortic (**A**; WT [wild type], n=6; mIGFREO, n=9) and pulmonary endothelial cell (PEC; **B**; WT, n=7; mIGFREO, n=5) levels of miR-25 mRNA expression in mIGFREO and WT littermates (WT). **C**, miR-25 mRNA level in SVEC from patients with and without diabetes (no diabetes, n=7; diabetes, n=5). **D** and **E**, miR-25 mRNA levels (**D**; no diabetes, n=5; diabetes, n=5) and Nox4 protein (**E**; WT, n=7; mIGFR, n=6) levels in SVEC from patients without diabetes, transfected with mIGF-1R (mouse IGF-1 receptor) cDNA compared with WT-cDNA. **F**, Nox4 mRNA expression in SVEC from patients without diabetes transfected with miR-25 mimetic or scrambled control (scrambled, n=5; mimetic, n=5. Schematic representation showing conceptual framework for insulin/IGF-1 resistance in endothelial cells leading to decreased miR-25 and a concomitant increase in Nox4, hydrogen peroxide (H_2_O_2_), leading to enhanced whole-body insulin sensitivity (Graphical Abstract). Data expressed as mean±SEM. *P<0.05, WT vs mIGFREO or no diabetes vs diabetes or WT-cDNA vs mIGF-1R-cDNA or scrambled vs miR-25 mimetic. Data were analyzed using unpaired Student *t* test. Data in **D** were analyzed using unpaired Student *t* test, Mann-Whitney *U* test.

Unlike other Nox isoforms, Nox4 activity is dependent primarily on its abundance. While transcriptional regulation of Nox4 is incompletely understood, a number of regulators of Nox4 expression, both negative and positive, have been proposed.^14^ One such transcriptional regulator is the microRNA mIR-25, which has been suggested to be a negative regulator of Nox4 transcription. We measured mIR-25 level in aorta and PEC of mIGFREO mice and found it was significantly reduced compared with WT littermates (Figure 8A and 8B). Moreover, miR-25 level was higher in SVEC of patients with diabetes (Figure 8C), and, importantly, when we transfected SVEC from patients without diabetes with mutant IGF-1R, miR-25 expression was significantly reduced with a reciprocal increase in the Nox4 expression (Figure 8D and 8E). Finally, we used a synthetic miR-25 mimetic to elevate its level directly in SVEC and found it significantly decreased mRNA expression of Nox4 (Figure 8F). Taken together, these results show that disrupting IGF-1R and IR signaling by mutant IGF-1R expression leads to increased Nox4 expression and H_2_O_2_ generation, by reducing miR-25 levels in the ECs (Graphic Abstract).

## Discussion

Lower order organisms have a single receptor (DAF-2 [DAuer Formation protein-2]) that transmits external cues to regulate glucose homeostasis, metabolism, and growth,^15^ whereas mammals have evolved to have 2 separate receptors that regulate glucose homeostasis (the IR) and growth (the IGF-1 receptor). Insulin may act in synergy with IGF-1 to coordinate responses to nutrient availability. An unexplained paradox exists whereby, in worms and flies, downregulation of DAF-2 has an advantageous effect on health, leading to stress resistance and extended life span,^15^ whereas in humans, insulin resistance leads to a range of chronic disorders of health. To date, no studies have examined the effect of the combination of diminished insulin and IGF-1 actions in vascular cells on metabolic and vascular homeostasis in mammals. To examine the effect of the combination of prolonged and selective insulin and IGF-1 resistance at the level of the endothelium, we generated a transgenic mouse expressing mutant IGF-1R that forms nonfunctioning hybrid receptors with native IRs and IGF-1R, specifically in ECs.

The following novel findings are reported: (1) ECs from humans with advanced atherosclerosis and type 2 diabetes are resistant to both insulin- and IGF-1–mediated eNOS activation; (2) EC-specific expression of mutant IGF-1R leads to resistance to insulin- and IGF1-mediated eNOS serine phosphorylation in ECs; (3) despite this, mIGFREO has enhanced glucose disposal in response to systemic insulin and IGF-1; (4) in contrast to mice with EC-specific insulin resistance,^7,8^ mIGFREO has reduced Nox2 Nox expression and increased Nox4 Nox expression at an mRNA and protein level; (5) catalase, which reduces H_2_O_2_, restores insulin-mediated glucose lowering to WT levels in mIGFREO; and (6) mIGFREO mice reveal the microRNA miR-25 as an important regulator of Nox4 expression; this was recapitulated in ECs from humans with type 2 diabetes and accelerated atherosclerosis.

### ECs From Humans With Advanced Atherosclerosis and Type 2 Diabetes Are Resistant to Both Insulin- and IGF-1–Mediated eNOS Serine Phosphorylation

As proof of principle, we examined the responses of SVECs from patients undergoing aortocoronary bypass surgery to insulin and IGF-1. We showed that, consistent with our preclinical studies, SVECs from humans with type 2 diabetes and advanced atherosclerosis have blunted serine phosphorylation of eNOS in response to both insulin and IGF-1. We also demonstrated decreased Nox4 Nox expression and increased Nox2 expression. This is perhaps not surprising when one considers that SVEC from patients with type 2 diabetes have been exposed to multiple systemic factors that are not present in mIGFREO (which have increased Nox4 and reduced Nox2 expression in EC) including, but not limited to, hyperglycemia, hyperlipidemia, hyperinsulinemia, and excess circulating cytokines. This notwithstanding, we demonstrate here for the first time that type 2 diabetes in humans is accompanied by both insulin and IGF-1 resistance at the level of the endothelium, providing a conceptual framework for the present study.

### Obesity Leads to Resistance to Insulin and IGF-1 in the Endothelium

Obesity and type 2 diabetes induce defects at multiple points in the insulin signaling pathway, resulting in resistance to insulin-mediated glucose uptake into skeletal muscle and other insulin target tissues.^1^ We have shown that whole-body genetic^7^ and diet-induced insulin resistance^5,10^ also lead to insulin resistance at the level of the endothelium. During the development of obesity and simultaneous insulin resistance, we have also demonstrated a similar decline in IGF-1 actions at a whole-body level^5,16^ and within the endothelium.^5^ While we have a deep understanding of the effects of whole-body and EC-specific insulin resistance on NO availability,^6–9,17^ the local and systemic consequences of prolonged insulin and IGF-1 resistance in the endothelium are unexplored. Moreover, the effects of endothelium-restricted insulin and IGF-1 resistance on systemic glucose homeostasis remain unclear.

### mIGFREO Mice Reveal Differential Sensitivity of Akt and eNOS to Insulin-Mediated Serine Phosphorylation in ECs

Akt is a critical node^18^ in the downstream insulin signaling pathway that lies proximal to eNOS. In ECs from humans with advanced atherosclerosis and type 2 diabetes, we showed that at 100-nM insulin-induced Akt phosphorylation was preserved, whereas eNOS phosphorylation was blunted. This scenario was recapitulated in mIGFREO mice. The role of Akt in insulin signaling in the endothelium in health and disease is of particular interest to the field. Elegant studies in adipocytes from the Accilli laboratory^19^ raised the possibility that activation of different nodes in the insulin signaling pathway downstream of the IR requires different concentrations of insulin, differing by as much as 10-fold to phosphorylate the protein sufficiently. This has not been examined in ECs from insulin-sensitive or insulin-resistant mammals. We, therefore, examined the effect of different concentrations of insulin on Akt and eNOS phosphorylation in ECs from mIGFREO mice and their WT littermates. Interestingly, we found that Akt was more sensitive to insulin than eNOS with a significant increase in phosphorylated Akt occurring at 50 nM, which did not lead to an increase in serine phosphorylated eNOS in WT or mIGFREO. These data are important to our understanding of insulin signaling in the endothelium in health and disease. The contrast between blunted insulin-induced eNOS activation seen in ECs from mIGFREO and the enhanced responses in resistance vessels most likely reflects the different mediators of relaxation in large and small arteries.^20^ Whereas NO has been shown to be most important in insulin-induced relaxation in large arteries,^8^ EDHF (endothelium-derived hyperpolarizing factor) has been shown to be the principal mediator of insulin-induced relaxation of small arteries.^21^ Our data demonstrating increased release of H_2_O_2_, a putative EDHF, from mIGFREO in response to insulin fit with the enhanced relaxation seen in second-order mesenteric arteries from mIGFREO.

### Insulin Resistance in the Endothelium and Glucose Intolerance

Insulin signaling in the endothelium has been suggested to be important in glucose uptake into skeletal muscle.^22^ In vitro studies have shown divergent results addressing the question of whether insulin signaling regulates glucose transport and metabolism in ECs. It has been shown that insulin signaling does not regulate glucose transport in human micro- and macrovascular ECs,^23^ bovine brain, and retinal ECs,^24,25^ whereas glucose transport and glycogen synthesis are increased by insulin in bovine ECs isolated from adipose tissue or retinas and rabbit ECs.^26–28^ Consistent with the mIGFREO phenotype we have described here, seminal in vivo studies from Vicent et al^29^ demonstrate that mice with EC depletion of IR display no changes in fed and fasting blood glucose levels and in fact, at 6 months of age, have better glucose tolerance than WT littermates, suggesting that normal glucose uptake is not dependent on insulin signaling in ECs. These data suggest that the presence of endothelial insulin and IGF-1 resistance may represent a potentially favorable adaptation to metabolic stress, leading to enhanced glucose uptake in response to insulin and a favorable effect on lipid profile.

### Reactive Oxygen Species as Signaling Molecules in Glucose Homeostasis

Some biomolecules may be modified by oxidation.^30^ Once specific types of oxidants are generated at a given time and place, they can mediate reversible and irreversible modifications in a range of molecules. In relation to insulin signaling, it is thought that inhibition of PTPs (phosphotyrosine phosphatases) by H_2_O_2_-mediated oxidation of cysteine residues is necessary for optimal signaling.^31–34^ It has also been shown that mild oxidative conditions enhance the activation of IGF-1R,^35^ suggesting that optimal insulin and IGF-1R responsiveness involves redox priming. Mice deficient in GPrx (glutathione peroxidase), which reduces H_2_O_2_ to water, provide evidence that H_2_O_2_ is important in insulin sensitivity. GPrx-deficient mice are protected against HFD-induced insulin resistance.^36^ H_2_O_2_ and Nox4 may, therefore, be components of a complex system of receptor tyrosine kinases/PTPs and oxidants that regulate insulin-mediated glucose lowering. Consistent with this hypothesis, we showed that the enhanced insulin-stimulated glucose disposal of mIGFREO was blunted by infusion of the H_2_O_2_ degrading enzyme, catalase. Moreover, we show that IRs in liver from mIGFREO treated with systemic insulin have increased tyrosine phosphorylation, which is reduced to WT levels by catalase.

A number of mechanisms may underpin EC release of H_2_O_2_ at levels that enhance whole-body insulin sensitivity in mIGFREO. We have shown increased expression of Nox4 NAD(P)H oxidase in ECs from mIGFREO. Nox4 is unique among the Nox in that it generates H_2_O_2_^37^ and is constitutively active, generating H_2_O_2_ at concentrations proportionate to its expression.^38^ Consistent with this, we have shown that increased EC expression of Nox4 leads to increased basal H_2_O_2_ release.^39^ In mIGFREO, as discussed above, this enhances insulin sensitivity by redox priming of IRs. Nox4 is also an insulin-responsive enzyme.^40^ When insulin binds to its receptor, it rapidly activates Nox4 to generate a transient burst of H_2_O_2_.^33,34^ This short-lived increment in H_2_O_2_ enhances insulin sensitivity by inhibition of PTP1B (protein tyrosine phosphatase 1B) and PTEN (phosphatase and tensin) both of which are negative regulators of insulin signaling.^33^

### Nox-Derived Oxidants as Signaling Molecules

The Nox are a group of enzymes whose specific function is to generate superoxide.^41^ Members of the family are named after the transmembrane protein Nox. All 7 Nox proteins share highly conserved structural features; despite this, Nox proteins differ in their mode of activation, their interaction with the transmembrane protein p22^*phox*^ and the requirement for additional maturation and activation factors.^41^ Nox4 is highly expressed in human ECs^42^ and unlike other Nox isoforms, is constitutively active and independent of cytosolic activator proteins or regulatory domains. Recent studies demonstrate that H_2_O_2_ is the principal oxidant generated by Nox4, rather than O_2_·^−^.^37^ In the present report, we identify a previously unidentified pathway, activated in the presence of an evolutionarily conserved response to cellular stress, that is, downregulation of insulin and IGF-1 signaling. We show that the combination of insulin and IGF-1 resistance increases Nox4 expression and leads to increased generation of the signaling oxidant H_2_O_2_, which in turn leads to redox priming of IRs in canonical insulin target tissues, enhancing whole-body insulin sensitivity and reducing fasting free fatty acid levels (see schematic representation in the Graphic Abstract).

### mIGFREO Mice Reveal the microRNA miR-25 as an Important Transcriptional Regulator of Nox4 That Is Dysregulated in Humans With Type 2 Diabetes and Advanced Atherosclerosis

Nox4 is thought to be unique among the Nox isoforms, in that the principal mechanism of regulation is transcriptional.^43^ Among a number of potential transcriptional regulators of the Nox4 expression, the microRNA miR-25 has emerged as potentially important in diabetes.^44^ mIR-25 has been shown to negatively regulate Nox4 expression in a number of studies.^45,46^ miRNAs are a class of noncoding RNAs that play a critical role in cell differentiation, proliferation, and survival by binding to complementary target mRNAs, leading to transcriptional inhibition or degradation.^47^ miRNAs are found to be dysregulated in a range of disorders associated with abnormal cellular growth and metabolism.^48^ We examined expression of mIR-25 in ECs and whole aorta from mIGFREO mice, consistent with increased Nox4 expression, we found mIR-25 to be decreased in both aorta and ECs. We, then examined the expression of mIR-25 in SVECs from patients with advanced atherosclerosis and type 2 diabetes, showing increased mIR-25 consistent with the reduced Nox4 seen in ECs from these patients. To take this a step further, we expressed mutant IGF-1R in SVECs from patients with advanced atherosclerosis and demonstrated a reduction in mIR-25 expression and an increase in Nox4 expression. Another intriguing finding in the present study was the reduced Nox2 in mIGFREO mice. Consistent with our findings, studies have shown that Nox4 may inhibit Nox2 expression.^49,50^ Our data set raises the possibility that by manipulating miR-25, it may be possible to change expression of Nox4 and insulin sensitivity at a whole-body level. An interesting initial experiment would be to administer miR-25 mimetic to mIGFREO mice, where the initial proof of concept would be a reduction in Nox4 and a commensurate decline in whole-body insulin sensitivity.

### Study Limitations

The *Tie-2* promoter has been shown on occasions to drive expression in populations of myeloid cells,^51^ although using a similar approach to generate mice overexpressing the IR in ECs,^8^ we did not demonstrate significant off-target expression. Consistent with this, we did not demonstrate significant expression of mIGF-1R in monocytes from mIGFREO (Figure I in the Data Supplement). While one could argue that the changes in glucose tolerance in mIGFREO mice are relatively small, in the context of normal glucose homeostasis, they remain striking and of therapeutic and physiological relevance.

### Conclusions

Here, we show that in the setting of insulin and IGF-1 resistance, the endothelium undergoes a phenotypic change underpinned by increased EC Nox4 Nox expression that augments H_2_O_2_ generation. This H_2_O_2_ release is likely to act in a paracrine fashion to enhance insulin-mediated glucose lowering in skeletal muscle and brown adipose tissue; therefore, revealing novel cross talk between the endothelium and insulin-sensitive tissues. Thus, this data set significantly contributes to our understanding of the nature and mechanism of mammalian responses to metabolic stress and provides a new perspective in understanding of the regulation of the insulin/IGF-1 pathways under normal conditions and in the context of disease.

## Acknowledgments

We would like to acknowledge Dr Theresa Munyombwe, biostatistician from LICAMM (Leeds Institute for Cardiovascular and Metabolic Medicine, UK), for statistical advice; Samuel Tate, Annick Farrell, Luke Morris, and Daniel Smitham of the LICAMM University of Huddersfield Sandwich Year, and Nathan Sharlotte of University of Sheffield-Hallam Sandwich Year Programme for their invaluable technical support in this project.

## Sources of Funding

This work was supported by the British Heart Foundation (BHF) project grant to P. Sukumar (PG/17/16/32853). M.T. Kearney is BHF Professor of Cardiovascular and Diabetes Research (CH/13/1/30086). S.B. Wheatcroft was supported by a European Research Council starter award 310747. R.M. Cubbon is supported by the BHF Intermediate Research Fellowship FS/12/80/29821, and D.J. Beech is a Wellcome Trust Investigator (110044/Z/15/Z). A.M. Shah is a BHF Professor of Cardiology (CH/1999001/11735). Y. Abudushalamu received a BHF 4-Year PhD Studentship. N. Endesh was supported by a Wellcome Trust Investigator award to David J. Beech.

## Disclosures

None.

## Supplemental Materials

Data Supplement Tables

Data Supplement Figures I–VII

Major Resources Table

Full Unedited Blots

References 52–59

## Supplementary Material



## Novelty and Significance

### What Is Known?

- Type 2 diabetes is characterized by an inability of both insulin and IGF-1 (insulin-like growth factor-1) to appropriately activate their intracellular signaling networks in the endothelium and other tissues.
- The combination of insulin and IGF-1 resistance in skeletal muscle in mice, due to expression of a mIGF-1R (mouse IGF-1 receptor), which forms nonfunctioning hybrids with native IR (insulin receptor) and IGF-1Rs (IGF-1 receptors), leads to type 2 diabetes.
- The effect of the combination of insulin and IGF-1 resistance specific to the endothelium at a cellular and systemic level remains unexplored.

## References

[1] Johnson AM Olefsky JM. The origins and drivers of insulin resistance. Cell. 2013;152:673–684. doi: 10.1016/j.cell.2013.01.041 23415219 10.1016/j.cell.2013.01.041

[2] Fulton D Gratton JP McCabe TJ Fontana J Fujio Y Walsh K Franke TF Papapetropoulos A Sessa WC. Regulation of endothelium-derived nitric oxide production by the protein kinase Akt. Nature. 1999;399:597–601. doi: 10.1038/21218 10376602 10.1038/21218 PMC3637917

[3] Dimmeler S Fleming I Fisslthaler B Hermann C Busse R Zeiher AM. Activation of nitric oxide synthase in endothelial cells by Akt-dependent phosphorylation. Nature. 1999;399:601–605. doi: 10.1038/21224 10376603 10.1038/21224

[4] Tan KT Luo SC Ho WZ Lee YH. Insulin/IGF-1 receptor signaling enhances biosynthetic activity and fat mobilization in the initial phase of starvation in adult male C. elegans. Cell Metab. 2011;14:390–402. doi: 10.1016/j.cmet.2011.06.019 21907144 10.1016/j.cmet.2011.06.019

[5] Imrie H Abbas A Viswambharan H Rajwani A Cubbon RM Gage M Kahn M Ezzat VA Duncan ER Grant PJ. Vascular insulin-like growth factor-I resistance and diet-induced obesity. Endocrinology. 2009;150:4575–4582. doi: 10.1210/en.2008-1641 19608653 10.1210/en.2008-1641

[6] Duncan ER Crossey PA Walker S Anilkumar N Poston L Douglas G Ezzat VA Wheatcroft SB Shah AM Kearney MT. Effect of endothelium-specific insulin resistance on endothelial function in vivo. Diabetes. 2008;57:3307–3314. doi: 10.2337/db07-1111 18835939 10.2337/db07-1111 PMC2584137

[7] Sukumar P Viswambharan H Imrie H Cubbon RM Yuldasheva N Gage M Galloway S Skromna A Kandavelu P Santos CX. Nox2 NADPH oxidase has a critical role in insulin resistance-related endothelial cell dysfunction. Diabetes. 2013;62:2130–2134. doi: 10.2337/db12-1294 23349484 10.2337/db12-1294 PMC3661635

[8] Viswambharan H Yuldasheva NY Sengupta A Imrie H Gage MC Haywood N Walker AM Skromna A Makova N Galloway S. Selective enhancement of insulin sensitivity in the endothelium in vivo reveals a novel proatherosclerotic signaling loop. Circ Res. 2017;120:784–798. doi: 10.1161/CIRCRESAHA.116.309678 27920123 10.1161/CIRCRESAHA.116.309678

[9] Watt NT Gage MC Patel PA Viswambharan H Sukumar P Galloway S Yuldasheva NY Imrie H Walker AMN Griffin KJ. Endothelial SHIP2 suppresses Nox2 NADPH oxidase-dependent vascular oxidative stress, endothelial dysfunction, and systemic insulin resistance. Diabetes. 2017;66:2808–2821. doi: 10.2337/db17-0062 28830894 10.2337/db17-0062

[10] Noronha BT Li JM Wheatcroft SB Shah AM Kearney MT. Inducible nitric oxide synthase has divergent effects on vascular and metabolic function in obesity. Diabetes. 2005;54:1082–1089. doi: 10.2337/diabetes.54.4.1082 15793247 10.2337/diabetes.54.4.1082

[11] Fernández AM Kim JK Yakar S Dupont J Hernandez-Sanchez C Castle AL Filmore J Shulman GI Le Roith D. Functional inactivation of the IGF-I and insulin receptors in skeletal muscle causes type 2 diabetes. Genes Dev. 2001;15:1926–1934. doi: 10.1101/gad.908001 11485987 10.1101/gad.908001 PMC312754

[12] Mughal RS Bridge K Buza I Slaaby R Worm J Klitgaard-Povlsen G Hvid H Schiødt M Cubbon R Yuldasheva N. Effects of obesity on insulin: insulin-like growth factor 1 hybrid receptor expression and Akt phosphorylation in conduit and resistance arteries. Diab Vasc Dis Res. 2019;16:160–170. doi: 10.1177/1479164118802550 30295509 10.1177/1479164118802550 PMC6484231

[13] Gangadhariah MH Dieckmann BW Lantier L Kang L Wasserman DH Chiusa M Caskey CF Dickerson J Luo P Gamboa JL. Cytochrome P450 epoxygenase-derived epoxyeicosatrienoic acids contribute to insulin sensitivity in mice and in humans. Diabetologia. 2017;60:1066–1075. doi: 10.1007/s00125-017-4260-0 28352940 10.1007/s00125-017-4260-0 PMC5921930

[14] Santos CX Hafstad AD Beretta M Zhang M Molenaar C Kopec J Fotinou D Murray TV Cobb AM Martin D. Targeted redox inhibition of protein phosphatase 1 by Nox4 regulates eIF2α-mediated stress signaling. EMBO J. 2016;35:319–334. doi: 10.15252/embj.201592394 26742780 10.15252/embj.201592394 PMC4741303

[15] Apfeld J Kenyon C. Cell nonautonomy of C. elegans DAF-2 function in the regulation of diapause and life span. Cell. 1998;95:199–210. doi: 10.1016/s0092-8674(00)81751-1 9790527 10.1016/s0092-8674(00)81751-1

[16] Wheatcroft SB Kearney MT Shah AM Ezzat VA Miell JR Modo M Williams SC Cawthorn WP Medina-Gomez G Vidal-Puig A. IGF-binding protein-2 protects against the development of obesity and insulin resistance. Diabetes. 2007;56:285–294. doi: 10.2337/db06-0436 17259371 10.2337/db06-0436 PMC4295171

[17] Rask-Madsen C Li Q Freund B Feather D Abramov R Wu IH Chen K Yamamoto-Hiraoka J Goldenbogen J Sotiropoulos KB. Loss of insulin signaling in vascular endothelial cells accelerates atherosclerosis in apolipoprotein E null mice. Cell Metab. 2010;11:379–389. doi: 10.1016/j.cmet.2010.03.013 20444418 10.1016/j.cmet.2010.03.013 PMC3020149

[18] Taniguchi CM Emanuelli B Kahn CR. Critical nodes in signalling pathways: insights into insulin action. Nat Rev Mol Cell Biol. 2006;7:85–96. doi: 10.1038/nrm1837 16493415 10.1038/nrm1837

[19] Gonzalez E Flier E Molle D Accili D McGraw TE. Hyperinsulinemia leads to uncoupled insulin regulation of the GLUT4 glucose transporter and the FoxO1 transcription factor. Proc Natl Acad Sci USA. 2011;108:10162–10167. doi: 10.1073/pnas.1019268108 21646544 10.1073/pnas.1019268108 PMC3121842

[20] Matoba T Shimokawa H Nakashima M Hirakawa Y Mukai Y Hirano K Kanaide H Takeshita A. Hydrogen peroxide is an endothelium-derived hyperpolarizing factor in mice. J Clin Invest. 2000;106:1521–1530. doi: 10.1172/JCI10506 11120759 10.1172/JCI10506 PMC387255

[21] Miller AW Hoenig ME Ujhelyi MR. Mechanisms of impaired endothelial function associated with insulin resistance. J Cardiovasc Pharmacol Ther. 1998;3:125–134. doi: 10.1177/107424849800300205 10684490 10.1177/107424849800300205

[22] Kubota T Kubota N Kumagai H Yamaguchi S Kozono H Takahashi T Inoue M Itoh S Takamoto I Sasako T. Impaired insulin signaling in endothelial cells reduces insulin-induced glucose uptake by skeletal muscle. Cell Metab. 2011;13:294–307. doi: 10.1016/j.cmet.2011.01.018 21356519 10.1016/j.cmet.2011.01.018

[23] Artwohl M Brunmair B Fürnsinn C Hölzenbein T Rainer G Freudenthaler A Porod EM Huttary N Baumgartner-Parzer SM. Insulin does not regulate glucose transport and metabolism in human endothelium. Eur J Clin Invest. 2007;37:643–650. doi: 10.1111/j.1365-2362.2007.01838.x 17635575 10.1111/j.1365-2362.2007.01838.x

[24] Takakura Y Kuentzel SL Raub TJ Davies A Baldwin SA Borchardt RT. Hexose uptake in primary cultures of bovine brain microvessel endothelial cells. I. Basic characteristics and effects of D-glucose and insulin. Biochim Biophys Acta. 1991;1070:1–10. doi: 10.1016/0005-2736(91)90139-y 1751515 10.1016/0005-2736(91)90139-y

[25] Betz AL Bowman PD Goldstein GW. Hexose transport in microvascular endothelial cells cultured from bovine retina. Exp Eye Res. 1983;36:269–277. doi: 10.1016/0014-4835(83)90011-8 6337860 10.1016/0014-4835(83)90011-8

[26] Bar RS Siddle K Dolash S Boes M Dake B. Actions of insulin and insulinlike growth factors I and II in cultured microvessel endothelial cells from bovine adipose tissue. Metabolism. 1988;37:714–720. doi: 10.1016/0026-0495(88)90003-0 3043144 10.1016/0026-0495(88)90003-0

[27] Gerritsen ME Burke TM Allen LA. Glucose starvation is required for insulin stimulation of glucose uptake and metabolism in cultured microvascular endothelial cells. Microvasc Res. 1988;35:153–166. doi: 10.1016/0026-2862(88)90059-3 3285141 10.1016/0026-2862(88)90059-3

[28] King GL Buzney SM Kahn CR Hetu N Buchwald S Macdonald SG Rand LI. Differential responsiveness to insulin of endothelial and support cells from micro- and macrovessels. J Clin Invest. 1983;71:974–979. doi: 10.1172/jci110852 6339562 10.1172/JCI110852 PMC436955

[29] Vicent D Ilany J Kondo T Naruse K Fisher SJ Kisanuki YY Bursell S Yanagisawa M King GL Kahn CR. The role of endothelial insulin signaling in the regulation of vascular tone and insulin resistance. J Clin Invest. 2003;111:1373–1380. doi: 10.1172/JCI15211 12727929 10.1172/JCI15211 PMC154437

[30] Finkel T. Signal transduction by reactive oxygen species. J Cell Biol. 2011;194:7–15. doi: 10.1083/jcb.201102095 21746850 10.1083/jcb.201102095 PMC3135394

[31] Goldstein BJ Mahadev K Kalyankar M Wu X. Redox paradox: insulin action is facilitated by insulin-stimulated reactive oxygen species with multiple potential signaling targets. Diabetes. 2005;54:311–321. doi: 10.2337/diabetes.54.2.311 15677487 10.2337/diabetes.54.2.311 PMC1464057

[32] Mahadev K Zilbering A Zhu L Goldstein BJ. Insulin-stimulated hydrogen peroxide reversibly inhibits protein-tyrosine phosphatase 1b in vivo and enhances the early insulin action cascade. J Biol Chem. 2001;276:21938–21942. doi: 10.1074/jbc.C100109200 11297536 10.1074/jbc.C100109200

[33] Goldstein BJ Mahadev K Wu X Zhu L Motoshima H. Role of insulin-induced reactive oxygen species in the insulin signaling pathway. Antioxid Redox Signal. 2005;7:1021–1031. doi: 10.1089/ars.2005.7.1021 15998257 10.1089/ars.2005.7.1021 PMC1434604

[34] Mahadev K Motoshima H Wu X Ruddy JM Arnold RS Cheng G Lambeth JD Goldstein BJ. The NAD(P)H oxidase homolog Nox4 modulates insulin-stimulated generation of H_2_O_2_ and plays an integral role in insulin signal transduction. Mol Cell Biol. 2004;24:1844–1854. doi: 10.1128/MCB.24.5.1844-1854.2004 14966267 10.1128/MCB.24.5.1844-1854.2004 PMC350558

[35] Handayaningsih AE Iguchi G Fukuoka H Nishizawa H Takahashi M Yamamoto M Herningtyas EH Okimura Y Kaji H Chihara K. Reactive oxygen species play an essential role in IGF-I signaling and IGF-I-induced myocyte hypertrophy in C2C12 myocytes. Endocrinology. 2011;152:912–921. doi: 10.1210/en.2010-0981 21239445 10.1210/en.2010-0981

[36] Loh K Deng H Fukushima A Cai X Boivin B Galic S Bruce C Shields BJ Skiba B Ooms LM. Reactive oxygen species enhance insulin sensitivity. Cell Metab. 2009;10:260–272. doi: 10.1016/j.cmet.2009.08.009 19808019 10.1016/j.cmet.2009.08.009 PMC2892288

[37] Takac I Schröder K Zhang L Lardy B Anilkumar N Lambeth JD Shah AM Morel F Brandes RP. The E-loop is involved in hydrogen peroxide formation by the NADPH oxidase Nox4. J Biol Chem. 2011;286:13304–13313. doi: 10.1074/jbc.M110.192138 21343298 10.1074/jbc.M110.192138 PMC3075677

[38] Lambeth JD. NOX enzymes and the biology of reactive oxygen. Nat Rev Immunol. 2004;4:181–189. doi: 10.1038/nri1312 15039755 10.1038/nri1312

[39] Ray R Murdoch CE Wang M Santos CX Zhang M Alom-Ruiz S Anilkumar N Ouattara A Cave AC Walker SJ. Endothelial Nox4 NADPH oxidase enhances vasodilatation and reduces blood pressure in vivo. Arterioscler Thromb Vasc Biol. 2011;31:1368–1376. doi: 10.1161/ATVBAHA.110.219238 21415386 10.1161/ATVBAHA.110.219238

[40] Anilkumar N Weber R Zhang M Brewer A Shah AM. Nox4 and nox2 NADPH oxidases mediate distinct cellular redox signaling responses to agonist stimulation. Arterioscler Thromb Vasc Biol. 2008;28:1347–1354. doi: 10.1161/ATVBAHA.108.164277 18467643 10.1161/ATVBAHA.108.164277

[41] Drummond GR Selemidis S Griendling KK Sobey CG. Combating oxidative stress in vascular disease: NADPH oxidases as therapeutic targets. Nat Rev Drug Discov. 2011;10:453–471. doi: 10.1038/nrd3403 21629295 10.1038/nrd3403 PMC3361719

[42] Datla SR Peshavariya H Dusting GJ Mahadev K Goldstein BJ Jiang F. Important role of Nox4 type NADPH oxidase in angiogenic responses in human microvascular endothelial cells in vitro. Arterioscler Thromb Vasc Biol. 2007;27:2319–2324. doi: 10.1161/ATVBAHA.107.149450 17717289 10.1161/ATVBAHA.107.149450

[43] Chen F Haigh S Barman S Fulton DJ. From form to function: the role of Nox4 in the cardiovascular system. Front Physiol. 2012;3:412. doi: 10.3389/fphys.2012.00412 23125837 10.3389/fphys.2012.00412 PMC3485577

[44] Fu Y Zhang Y Wang Z Wang L Wei X Zhang B Wen Z Fang H Pang Q Yi F. Regulation of NADPH oxidase activity is associated with miRNA-25-mediated NOX4 expression in experimental diabetic nephropathy. Am J Nephrol. 2010;32:581–589. doi: 10.1159/000322105 21071935 10.1159/000322105

[45] Varga ZV Kupai K Szűcs G Gáspár R Pálóczi J Faragó N Zvara A Puskás LG Rázga Z Tiszlavicz L. MicroRNA-25-dependent up-regulation of NADPH oxidase 4 (NOX4) mediates hypercholesterolemia-induced oxidative/nitrative stress and subsequent dysfunction in the heart. J Mol Cell Cardiol. 2013;62:111–121. doi: 10.1016/j.yjmcc.2013.05.009 23722270 10.1016/j.yjmcc.2013.05.009

[46] Oh HJ Kato M Deshpande S Zhang E Das S Lanting L Wang M Natarajan R. Inhibition of the processing of miR-25 by HIPK2-Phosphorylated-MeCP2 induces NOX4 in early diabetic nephropathy. Sci Rep. 2016;6:38789. doi: 10.1038/srep38789 27941951 10.1038/srep38789 PMC5150532

[47] Gebert LFR MacRae IJ. Regulation of microRNA function in animals. Nat Rev Mol Cell Biol. 2019;20:21–37. doi: 10.1038/s41580-018-0045-7 30108335 10.1038/s41580-018-0045-7 PMC6546304

[48] Vienberg S Geiger J Madsen S Dalgaard LT. MicroRNAs in metabolism. Acta Physiol (Oxf). 2017;219:346–361. doi: 10.1111/apha.12681 27009502 10.1111/apha.12681 PMC5297868

[49] Gray SP Di Marco E Kennedy K Chew P Okabe J El-Osta A Calkin AC Biessen EA Touyz RM Cooper ME. Reactive oxygen species can provide atheroprotection via NOX4-dependent inhibition of inflammation and vascular remodeling. Arterioscler Thromb Vasc Biol. 2016;36:295–307. doi: 10.1161/ATVBAHA.115.307012 26715682 10.1161/ATVBAHA.115.307012

[50] Gajos-Draus A Duda M Beręsewicz A. Cardiac and renal upregulation of Nox2 and NF-κB and repression of Nox4 and Nrf2 in season- and diabetes-mediated models of vascular oxidative stress in guinea-pig and rat. Physiol Rep. 2017;5:e13474. doi: 10.14814/phy2.13474 29084841 10.14814/phy2.13474 PMC5661235

[51] Murdoch C Tazzyman S Webster S Lewis CE. Expression of Tie-2 by human monocytes and their responses to angiopoietin-2. J Immunol. 2007;178:7405–7411. doi: 10.4049/jimmunol.178.11.7405 17513791 10.4049/jimmunol.178.11.7405

[52] Li J Bruns AF Hou B Rode B Webster PJ Bailey MA Appleby HL Moss NK Ritchie JE Yuldasheva NY. Orai3 surface accumulation and calcium entry evoked by vascular endothelial growth factor. Arterioscler Thromb Vasc Biol. 2015;35:1987–1994. doi: 10.1161/ATVBAHA.115.305969 26160956 10.1161/ATVBAHA.115.305969 PMC4548547

[53] Guillot PV Liu L Kuivenhoven JA Guan J Rosenberg RD Aird WC. Targeting of human eNOS promoter to the Hprt locus of mice leads to tissue-restricted transgene expression. Physiol Genomics. 2000;2:77–83. doi: 10.1152/physiolgenomics.2000.2.2.77 11015585 10.1152/physiolgenomics.2000.2.2.77

[54] Minami T Kuivenhoven JA Evans V Kodama T Rosenberg RD Aird WC. Ets motifs are necessary for endothelial cell-specific expression of a 723-bp Tie-2 promoter/enhancer in Hprt targeted transgenic mice. Arterioscler Thromb Vasc Biol. 2003;23:2041–2047. doi: 10.1161/01.ATV.0000089326.63053.9A 12893685 10.1161/01.ATV.0000089326.63053.9A

[55] Li S Ferber A Miura M Baserga R. Mitogenicity and transforming activity of the insulin-like growth factor-I receptor with mutations in the tyrosine kinase domain. J Biol Chem. 1994;269:32558–32564. 7798258

[56] Kato H Faria TN Stannard B Roberts CT Jr LeRoith D. Role of tyrosine kinase activity in signal transduction by the insulin-like growth factor-I (IGF-I) receptor. Characterization of kinase-deficient IGF-I receptors and the action of an IGF-I-mimetic antibody (alpha IR-3). J Biol Chem. 1993;268:2655–2661. 7679099

[57] Nabeebaccus AA Zoccarato A Hafstad AD Santos CX Aasum E Brewer AC Zhang M Beretta M Yin X West JA. Nox4 reprograms cardiac substrate metabolism via protein O-GlcNAcylation to enhance stress adaptation. JCI Insight. 2017;2:96184. doi: 10.1172/jci.insight.96184 29263294 10.1172/jci.insight.96184 PMC5752273

[58] Schröder K Zhang M Benkhoff S Mieth A Pliquett R Kosowski J Kruse C Luedike P Michaelis UR Weissmann N. Nox4 is a protective reactive oxygen species generating vascular NADPH oxidase. Circ Res. 2012;110:1217–1225. doi: 10.1161/CIRCRESAHA.112.267054 22456182 10.1161/CIRCRESAHA.112.267054

[59] Lyle AN Joseph G Fan AE Weiss D Landázuri N Taylor WR. Reactive oxygen species regulate osteopontin expression in a murine model of postischemic neovascularization. Arterioscler Thromb Vasc Biol. 2012;32:1383–1391. doi: 10.1161/ATVBAHA.112.248922 22492090 10.1161/ATVBAHA.112.248922 PMC3376537

