# Supplementary figures and images for: Novel Paracrine Action of Endothelium Enhances Glucose Uptake in Muscle and Fat
Source: Circ Res. 2021 Aug 20;129(7):720–34. doi: 10.1161/CIRCRESAHA.121.319517 (PMC8448413; doi:10.1161/CIRCRESAHA.121.319517)

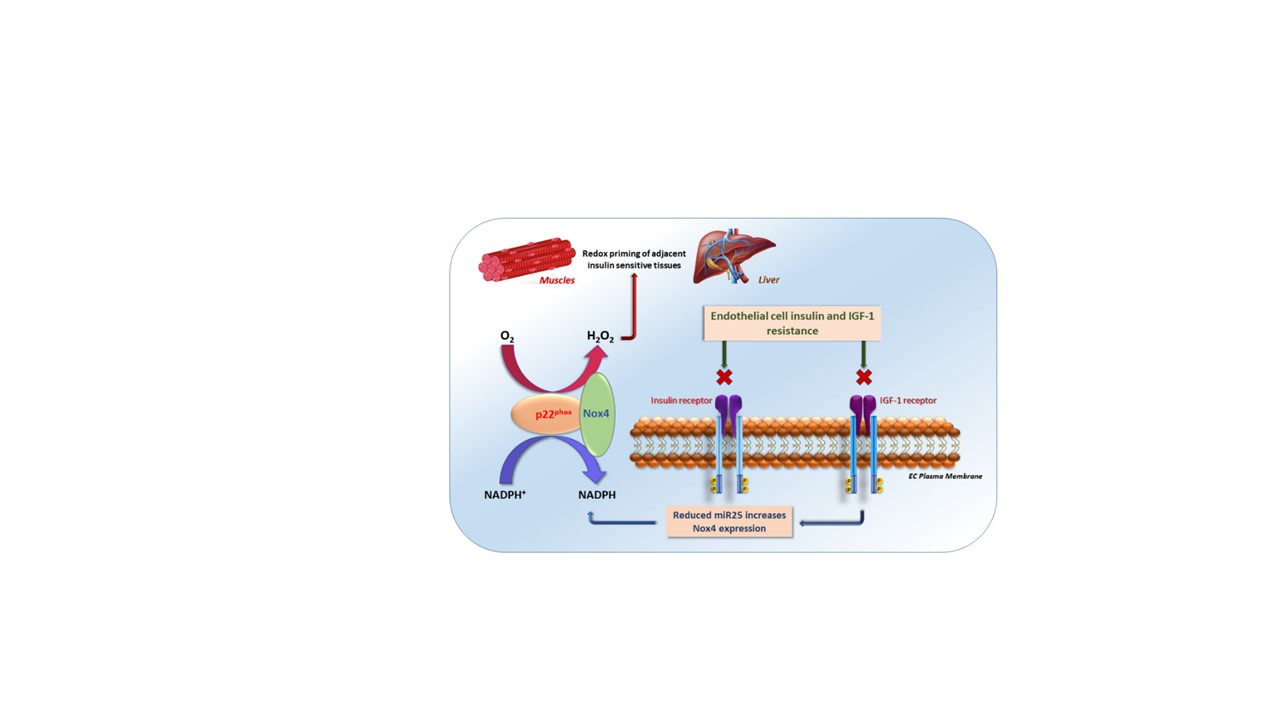

Supplement: Supplementary file 1 [file res-129-720-s001.jpg]
