# Supplementary material for: Novel Paracrine Action of Endothelium Enhances Glucose Uptake in Muscle and Fat
Source: Circ Res. 2021 Aug 20;129(7):720–34. doi: 10.1161/CIRCRESAHA.121.319517 (PMC8448413; doi:10.1161/CIRCRESAHA.121.319517)
Supplement: Supplementary file 2 [file res-129-720-s002.pdf]

Major Resources Table

I

Animals (in vivo studies)

Species	Vendor or Source	Background Strain	Sex	Persistent ID / URL
Mouse: C57BL/6 strain males for WT and mIGFREO	Breeding for experimental animal is in University of Leeds	C57BL/6	male	www.criver.com

Genetically Modified Animals

	Species	Vendor or Source	Backgro-und Strain	Other Information	Persistent ID / URL
Parent - Male	mouse	Breeding for experimental animal is in University of Leeds	C57Bl6	C57Bl6 was from Charles River, initially	criver.com
Parent - Female	mouse	Breeding for experimental animal is in University of Leeds	C57Bl6	C57Bl6 was from Charles River, initially	genoway.com

Antibodies

Target antigen	Vendor or Source	Catalog #	Working concentration	Persistent ID / URL
eNOS	BD Biosciences; Mouse	#610297	1:1000	https://www.bdbiosciences.com/en-us/products/reagents/microscopy-imaging-reagents/immunofluorescence-reagents/purified-mouse-anti-enos-nos-type-iii.610297
eNOS-pS1177	Cell Signaling; Rabbit	#9570	1:1000	https://www.cellsignal.co.uk/products/primary-antibodies/phospho-enos-ser1177-c9c3-rabbit-mab/9570
Akt	BD Biosciences; Mouse	#610861	1:1000	https://www.bdbiosciences.com/en-us/products/reagents/mic

				roscopy-imaging-reagents/immunofluorescence-reagents/purified-mouse-anti-akt.610861
Akt-pS473	Cell Signaling; Rabbit	#4060	1:1000	https://www.cellsignal.co.uk/products/primary-antibodies/phospho-akt-ser473-d9e-xp-rabbit-mab/4060
ERK-phospho (Thr202/ Tyr204)	Cell Signaling; Rabbit	#9101	1:1000	https://www.cellsignal.co.uk/products/primary-antibodies/phospho-p44-42-mapk-erk1-2-thr202-tyr204-antibody/9101?_=1626683098667&Ntt=9101&tahead=true
Tubulin	Santa Cruz Biotech; mouse	sc-5286	1:3000	https://www.scbt.com/p/alpha-tubulin-antibody-b-7
Beta-Actin	Santa Cruz Biotech; mouse	sc-47778	1:3000	https://www.scbt.com/p/beta-actin-antibody-ac-15
Nox2	AbCam; rabbit	ab129068	1:1000	https://www.abcam.com/nox2gp91phox-antibody-epr6991-ab129068.html
Nox2	BD Biosciences; mouse	#611414	1:1000	https://www.bdbiosciences.com/en-us/products/reagents/microscopy-imaging-reagents/immunofluorescence-reagents/purified-mouse-anti-gp91-phox.611414
Insulin Receptor	Cell Signaling; Rabbit	#3025	1:100	https://www.cellsignal.co.uk/products/primary-antibodies/insulin-receptor-b-4b8-rabbit-mab/3025
IGF1 receptor	Cell Signaling; Rabbit	#9750	1:100	https://www.cellsignal.co.uk/products/primary-antibodies/igf-i-receptor-b-d23h3-xp-rabbit-mab/9750
Phospho-INSR (Tyr1334)	Thermo Fisher	44-809G	1:1000	https://www.thermofisher.com/antibody/product/Phospho-INSR-Tyr1334-Antibody-Polyclonal/44-809G

Anti-Phospho-tyrosine, 4G10	Sigma-Aldrich, Millipore	05-1050X	1:2000	https://www.sigmaaldrich.com/GB/en/product/mm/051050x
ECL Mouse IgG, HRP	Amersham	NA931V	1:5000	https://www.cytivalifesciences.com/en/us/shop/protein-analysis/blotting-and-detection/blotting-standards-and-reagents/amersham-ecl-hrp-conjugated-antibodies-p-06260#overview
ECL Rabbit IgG, HRP	Amersham	NA934V	1:5000	https://www.sigmaaldrich.com/GB/en/product/sigma/gena9341ml?gclid=Cj0KCQjwxdSHBhCdARIsAG6zhlXgbjeJpWLL4Va1TiPAo-xVjw9D-07PwRLEESEPZJUNQelUpHlu9lMaAjrVEALw_wcB
eNOS-Ty657	ECM Biosciences; Rabbit	NP4031	1:1000	https://ecmbio.com/products/np4031
NOX4	Kind gift from prof Ajay Shah; Rabbit		1:2000	Santos CX et al. EMBO J. 2016; 35:319-34
ERK	Cell Signaling; Mouse	#4696	1:1000	https://www.cellsignal.co.uk/products/primary-antibodies/p44-42-mapk-erk1-2-l34f12-mouse-mab/4696

Cultured Cells

Name	Vendor or Source	Sex (F, M, or unknown)	Persistent ID / URL
Human Umbilical Vein Endothelial Cells (HUVECs)	Lonza Catalog #: CC-2935	F	
Saphenous Vein Endothelial cells	Gift of Dr Karen Porter	F and M	

Data & Code Availability

Description	Source / Repository	Persistent ID / URL
All datasets generated or analyzed during this study are included in the published article. All data are available from the Lead Contact upon reasonable request.		
