# Supplementary material for: Novel Paracrine Action of Endothelium Enhances Glucose Uptake in Muscle and Fat
Source: Circ Res. 2021 Aug 20;129(7):720–34. doi: 10.1161/CIRCRESAHA.121.319517 (PMC8448413; doi:10.1161/CIRCRESAHA.121.319517)

Full unedited gel for Figures

Novel Paracrine Action of Endothelium enhances Glucose Uptake in Muscle and Fat

[1]*Hema Viswambharan PhD, [1]*Nadira Yuldasheva PhD, [1]Helen Imrie PhD, [1]Katherine Bridge MBChB, PhD, [1]Natalie Haywood PhD, [1]Anna Skromna MSc, [1]Karen Hemmings PhD, [1]Emily Clark PhD, [1]V Kate Gatenby MBChB, PhD, [1]Paul Cordell PhD, [1]Katie J Simmons PhD, [1]Natallia Makava BSc, [1]Yilizila Abudushalamu MSc, [1]Naima Endesh PhD, [1]Jane Brown BSc, [1]Andrew Walker MBChB, PhD, [1]Simon T Futers PhD, [1]Karen E Porter PhD, [1]Richard M Cubbon PhD, [1]Khalid Naseem BSc, PhD, [2]Ajay M Shah MD, FMedSci, [1]David J Beech PhD, FMedSci, [1]Stephen B Wheatcroft MBChB, PhD, [1]Mark T Kearney DM, FRCP, [1]Piruthivi Sukumar MBBS, PhD.

[1]Leeds Institute for Cardiovascular and Metabolic Medicine, University of Leeds, United Kingdom.
[2] British Heart Foundation Centre of Research Excellence, Kings College London.

Full unedited gel for Figure 1G

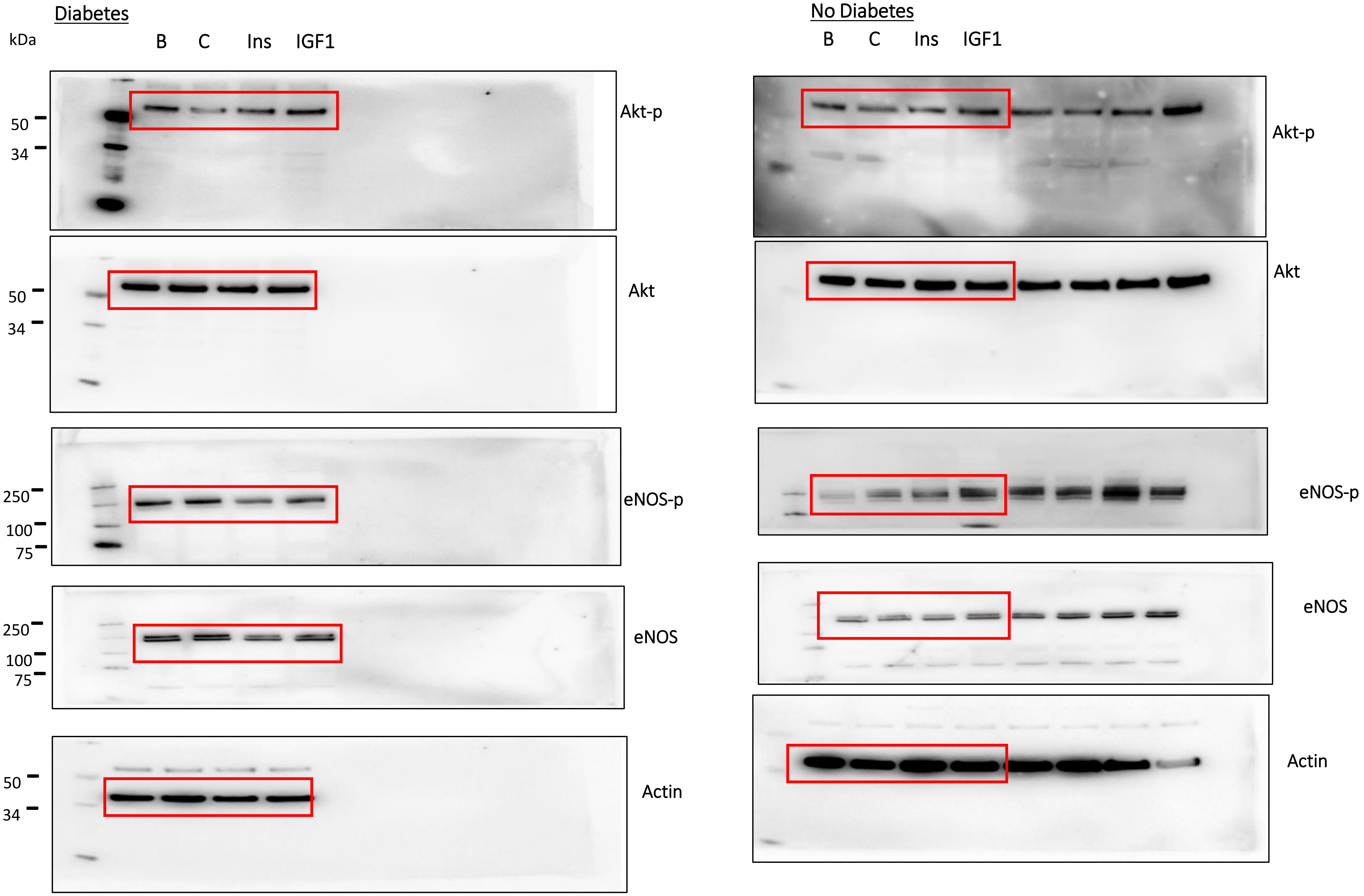

Full unedited gel for Figure 1H

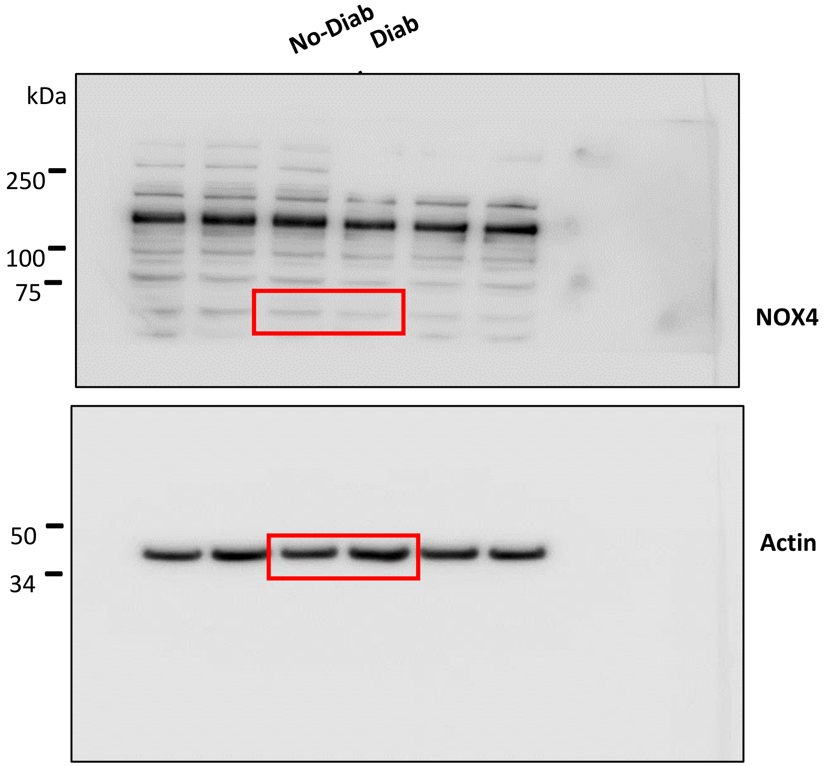

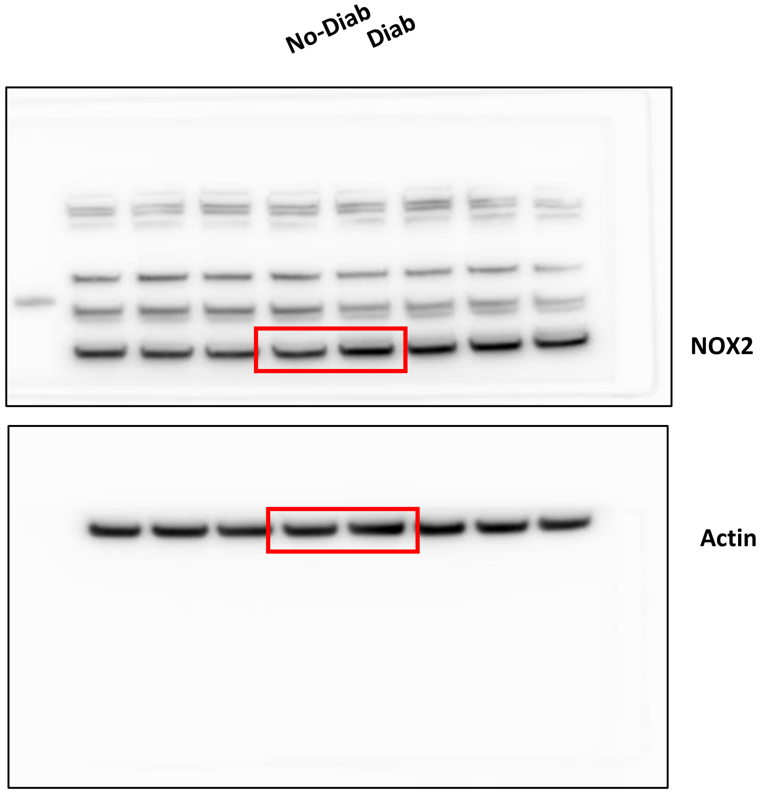

Full unedited gel for Figure 2E

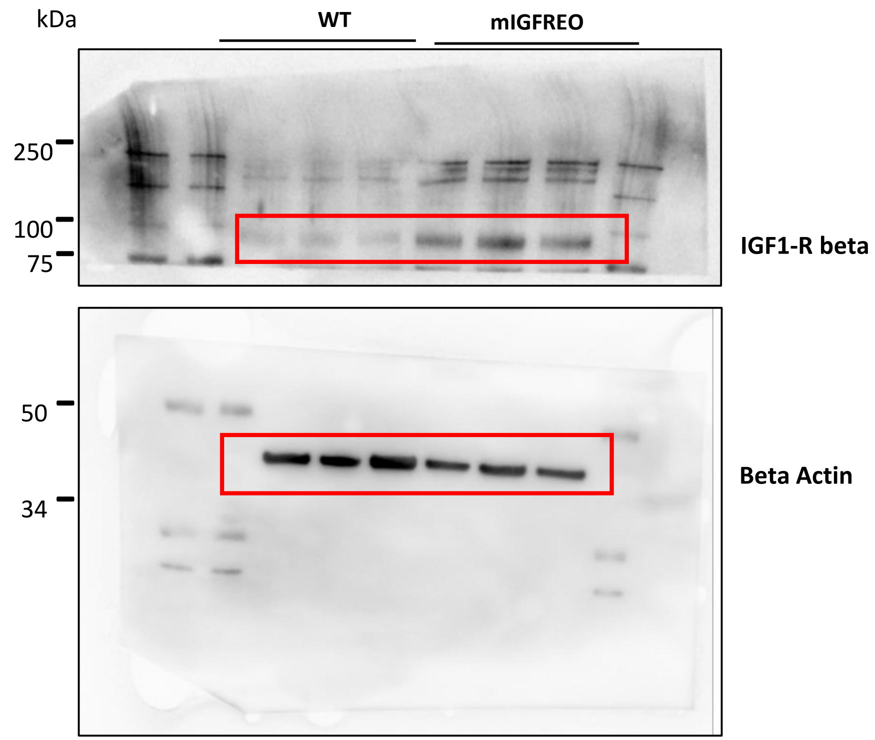

Full unedited gel for Figure 2F

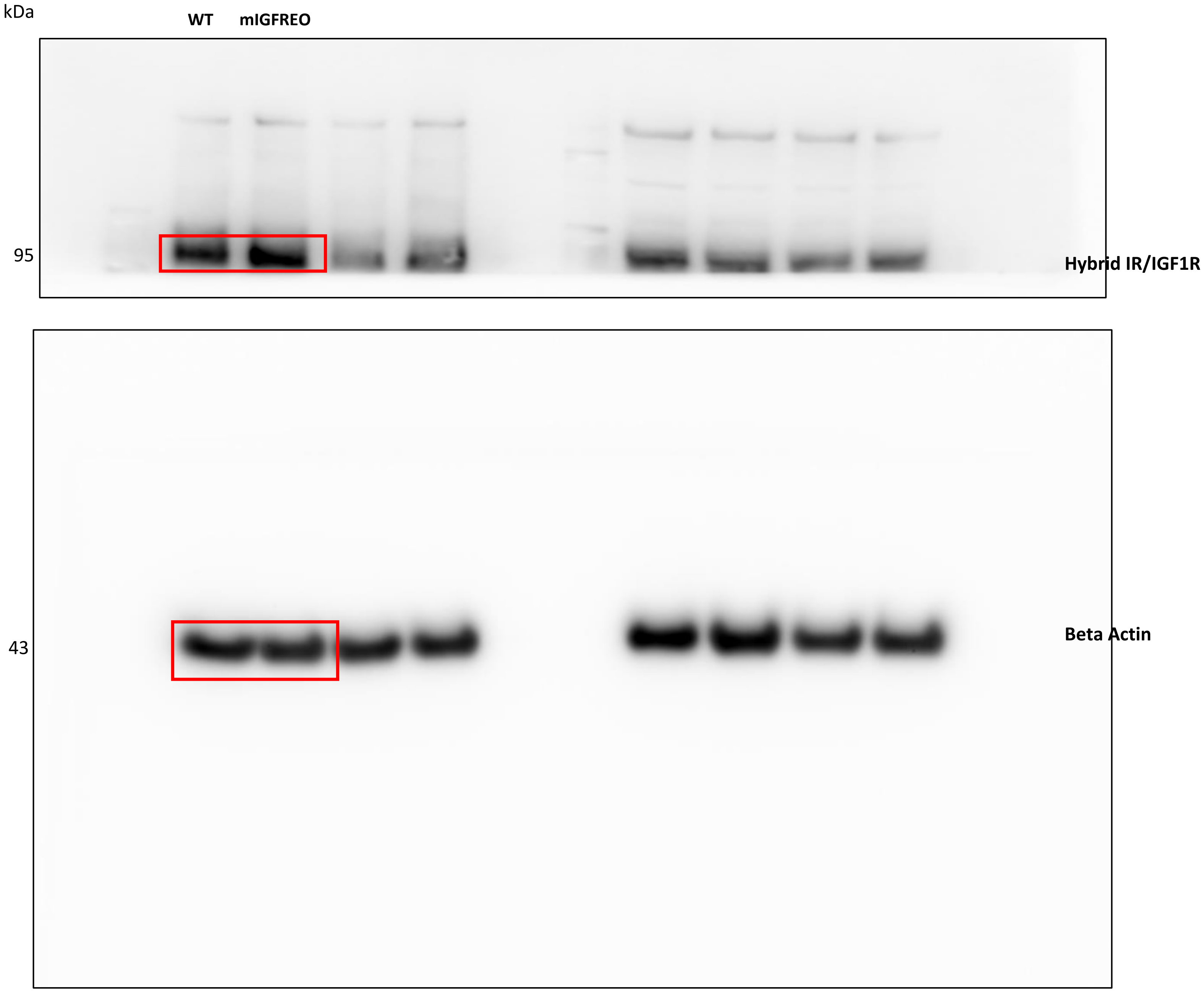

Full unedited gel for Figure 2G

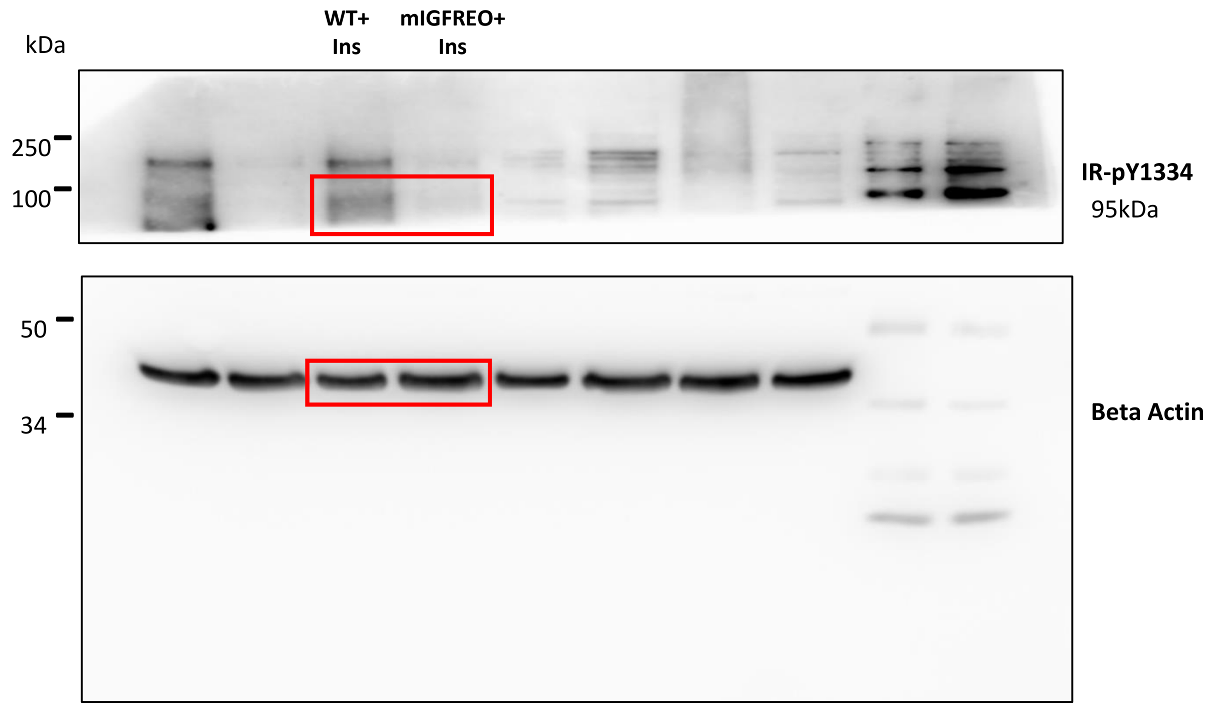

Full unedited gel for Figure 2H

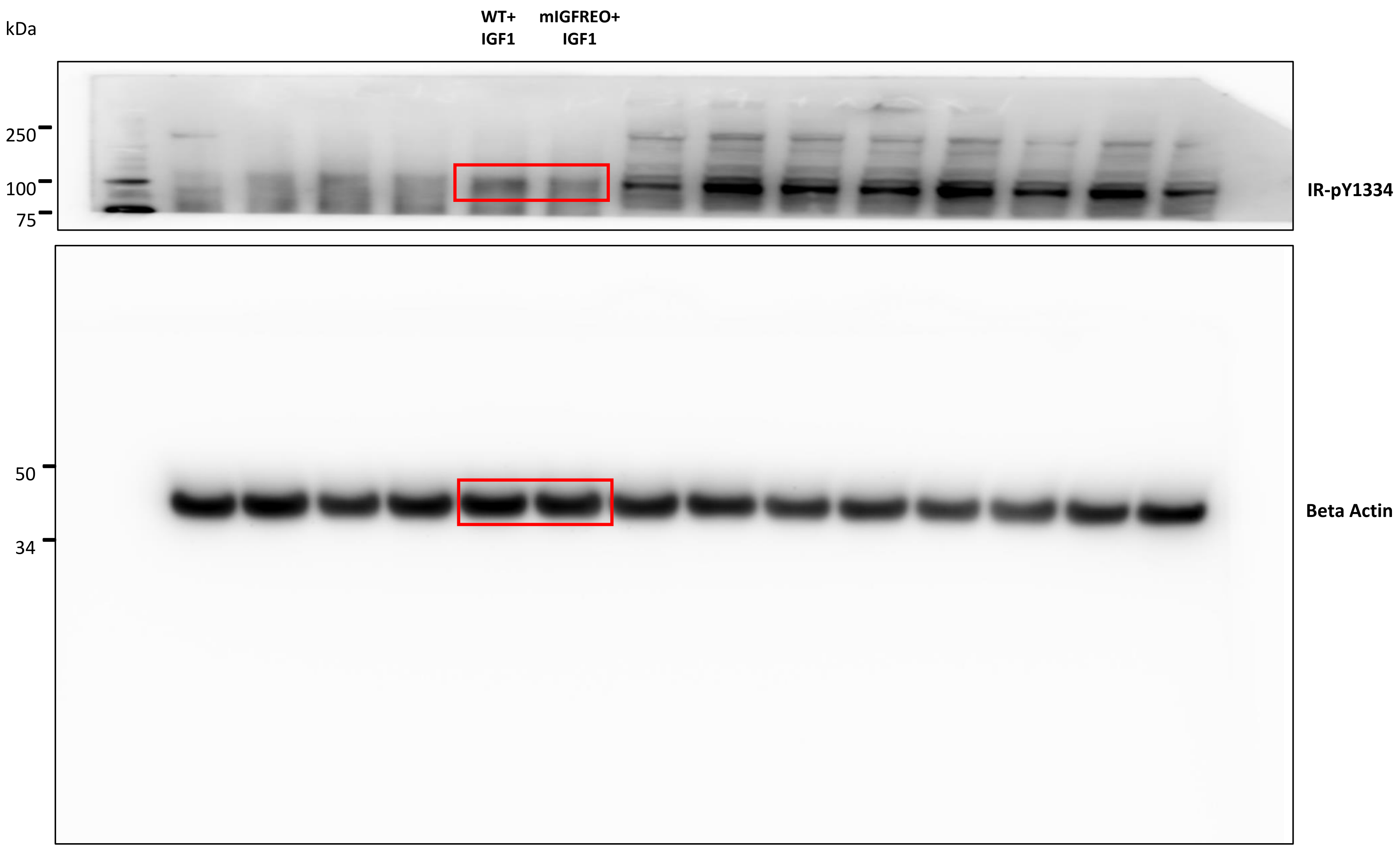

Full unedited gel for Figure 3A

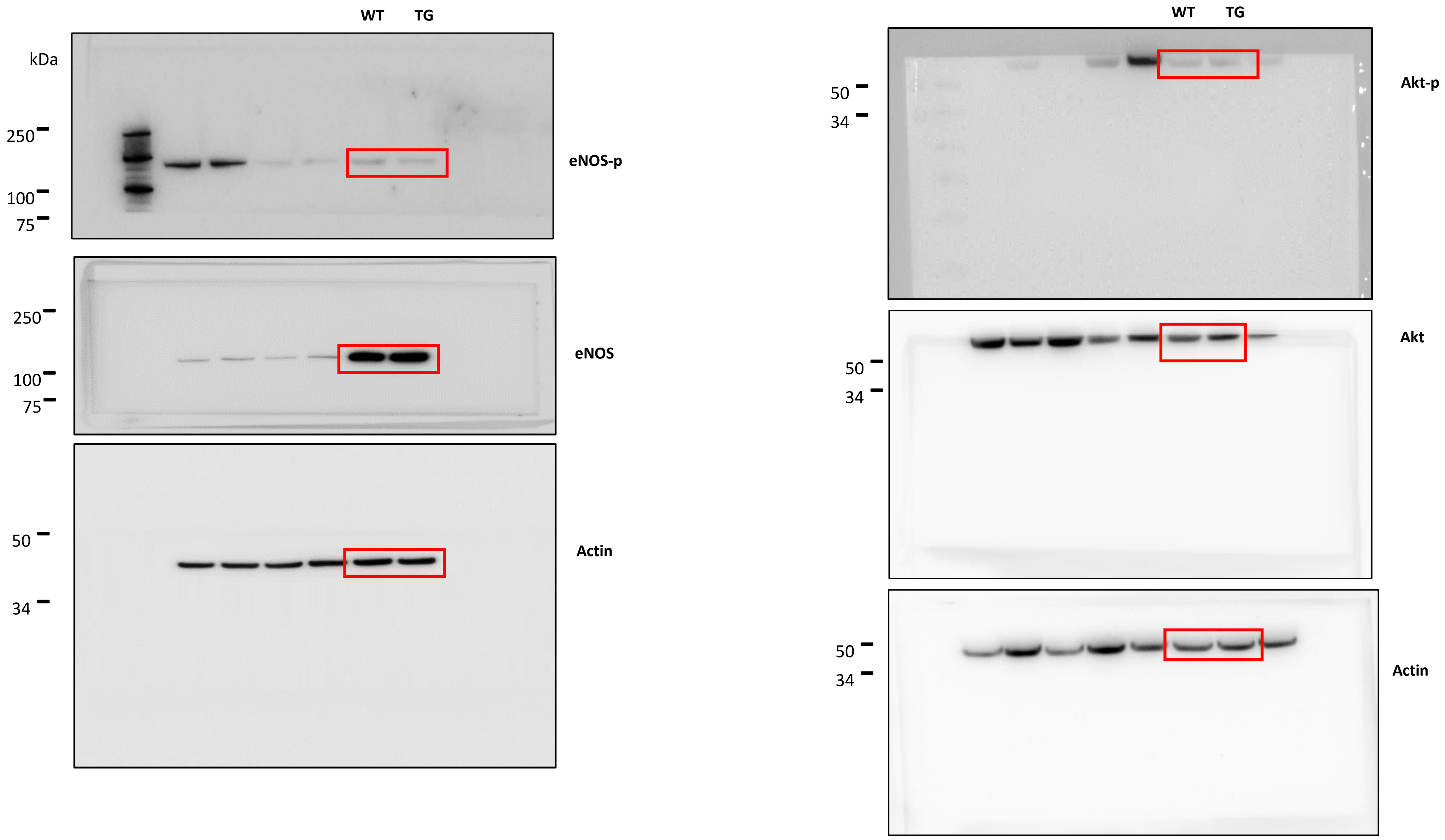

Full unedited gel for Figure 4A

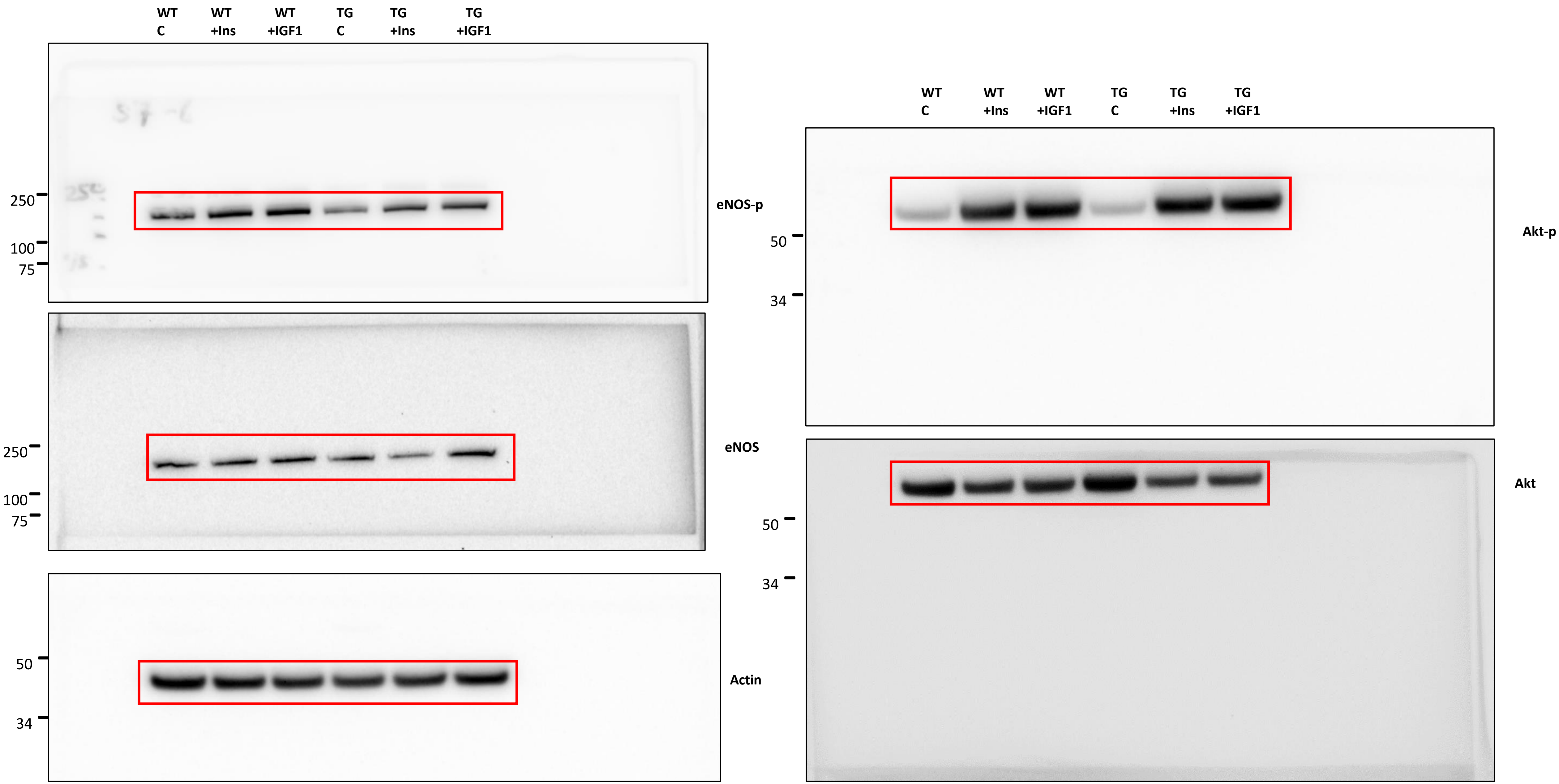

Full unedited gel for Figure 4F

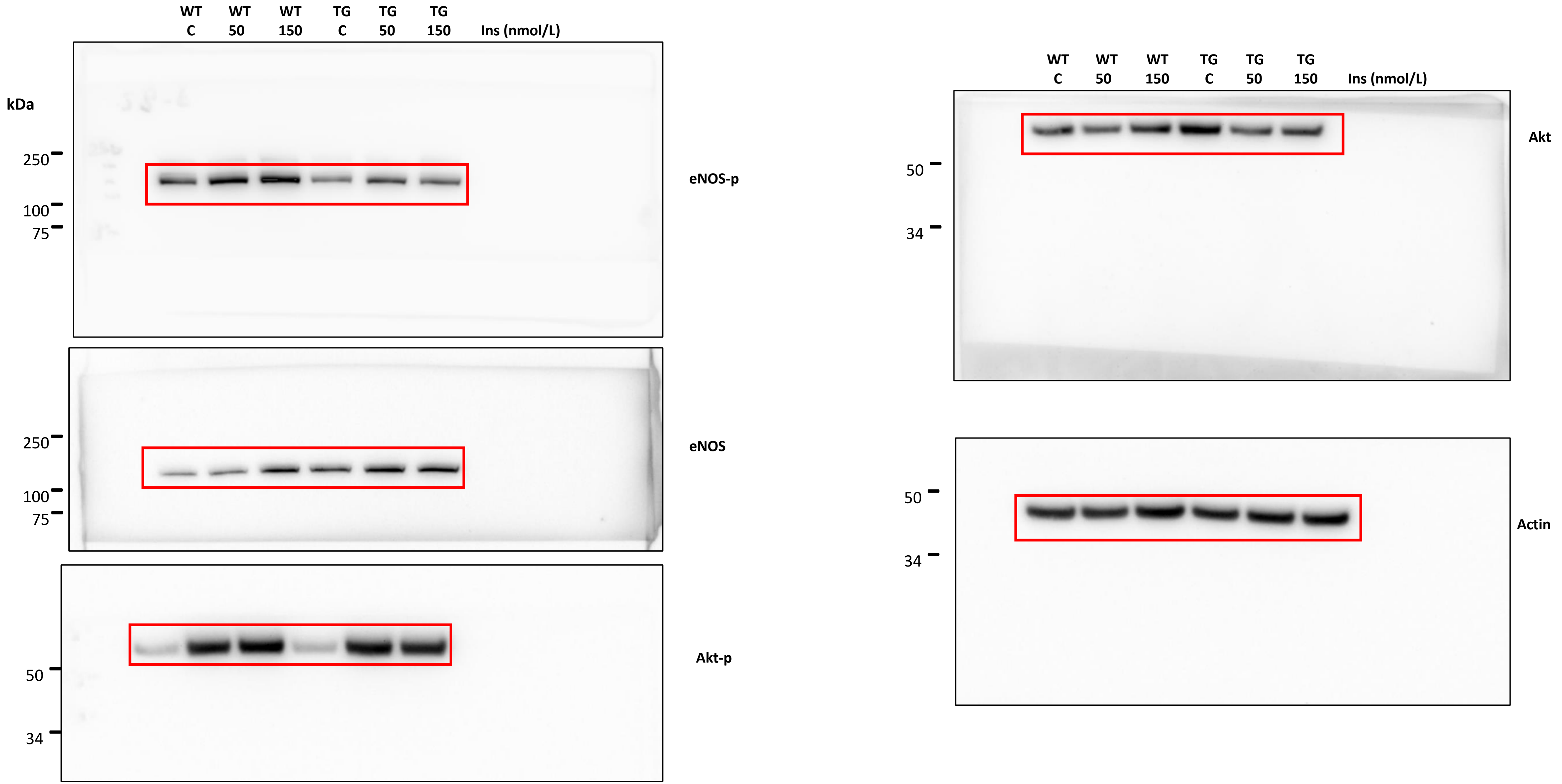

Full unedited gel for Figure 6G

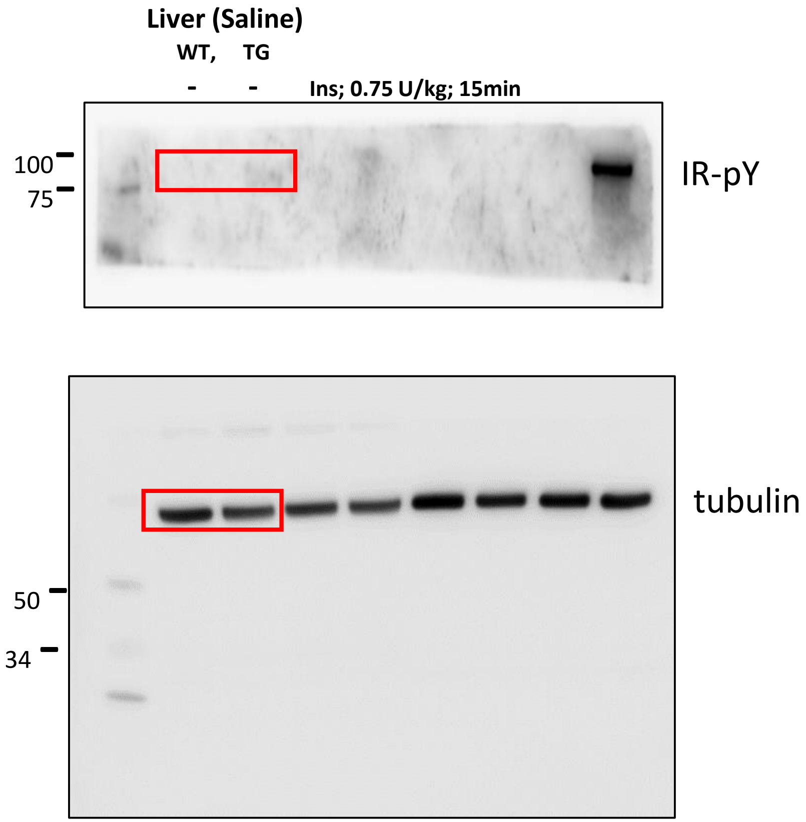

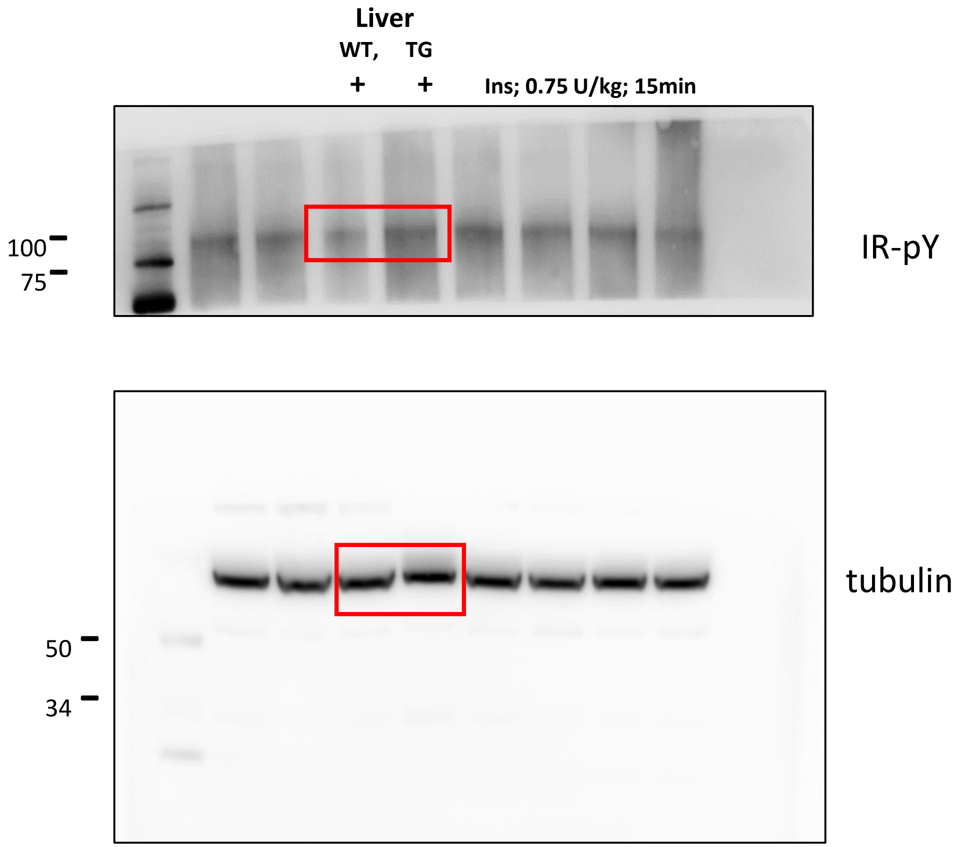

Full unedited gel for Figure 6H

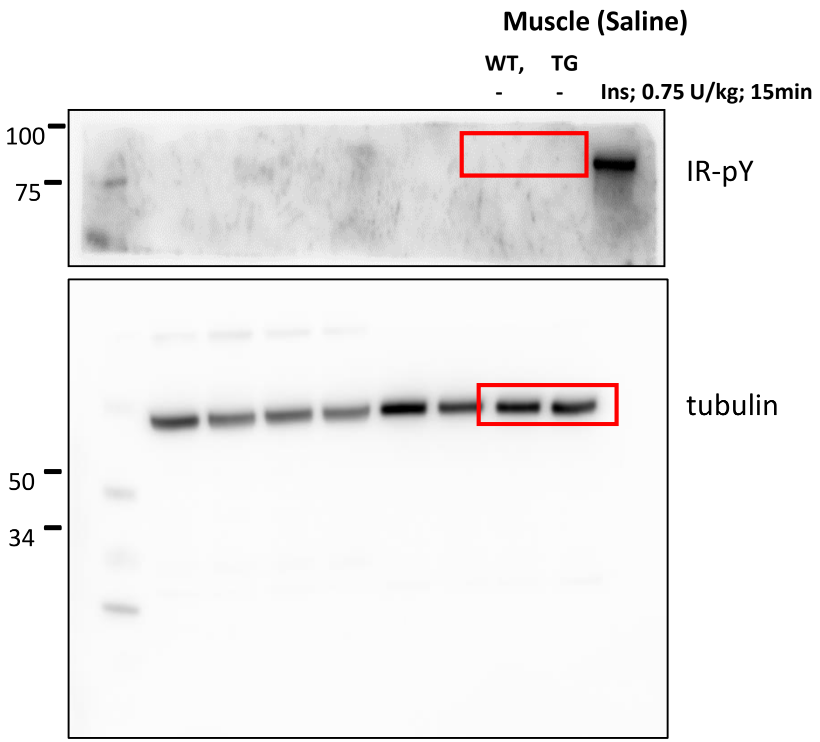

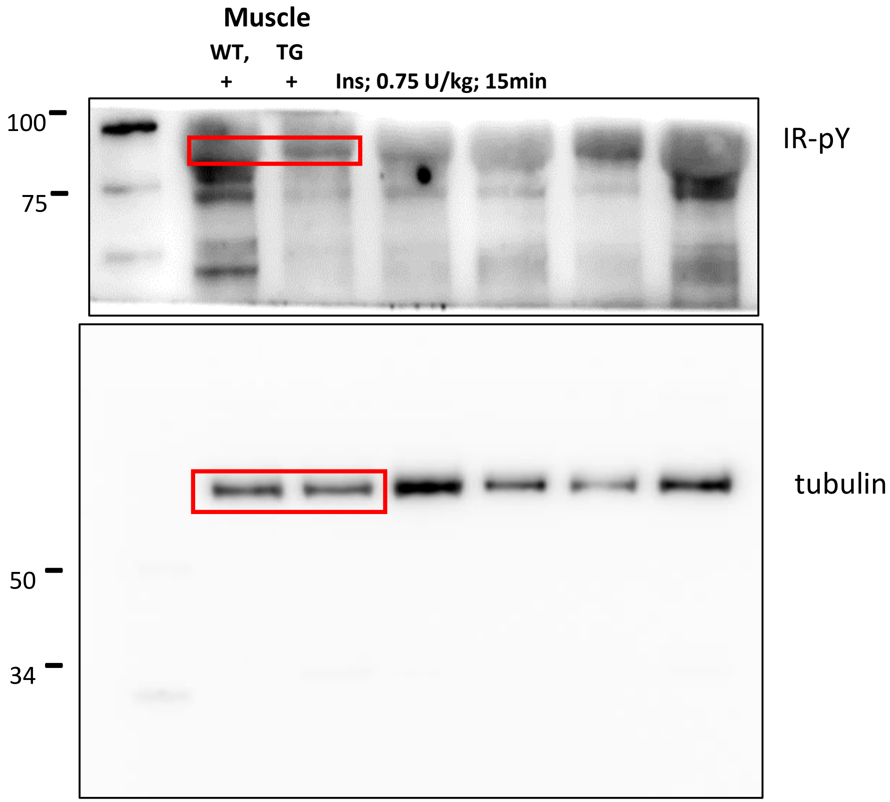

Full unedited gel for Figure 8D

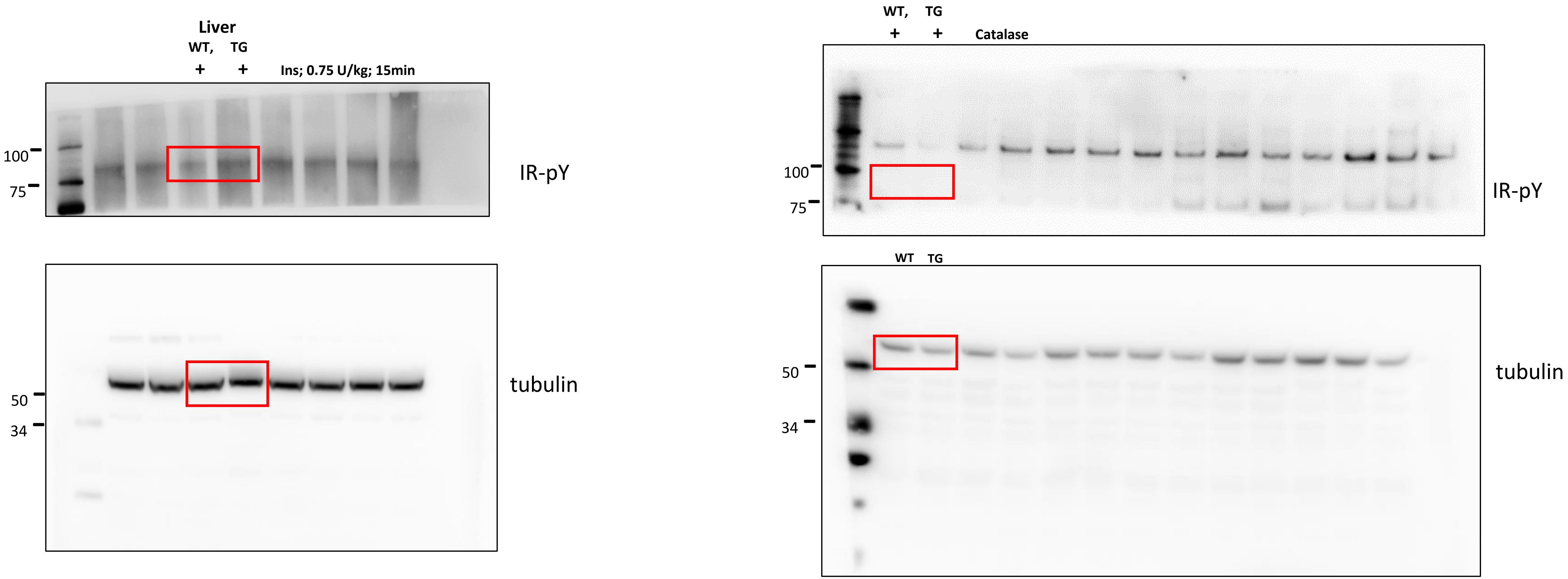

Full unedited gel for Figure 8G

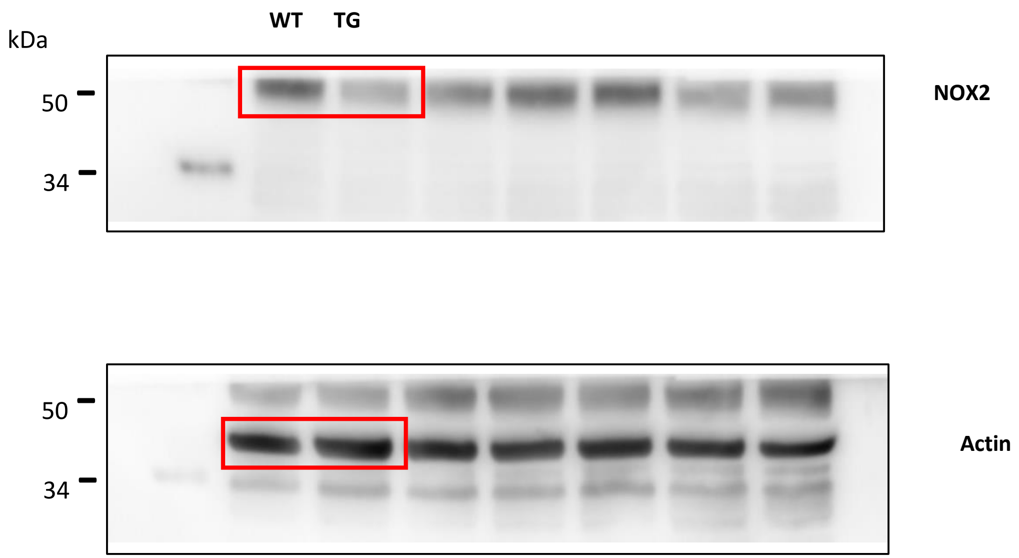

Full unedited gel for Figure 8H

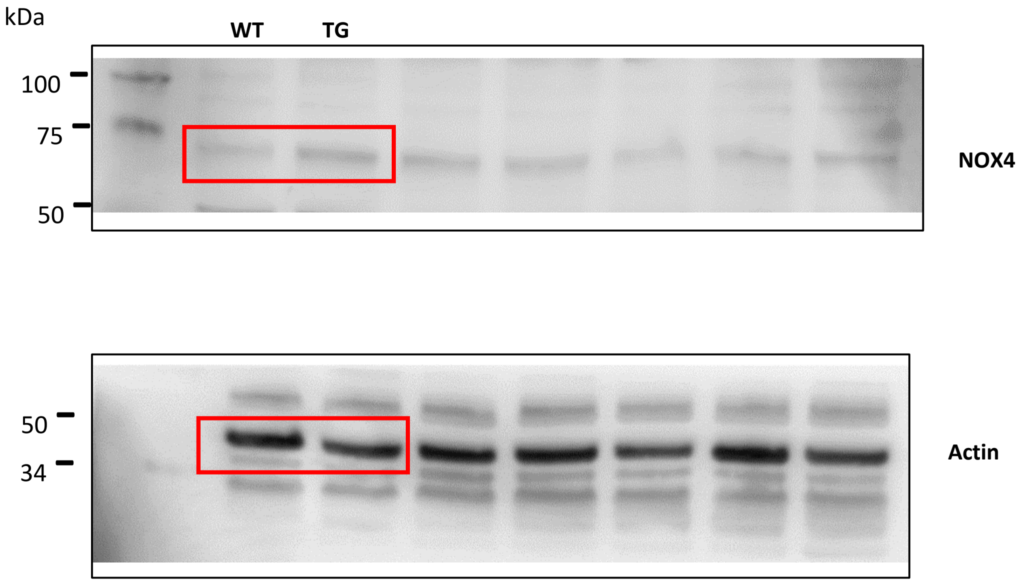

Full unedited gel for Figure 9E

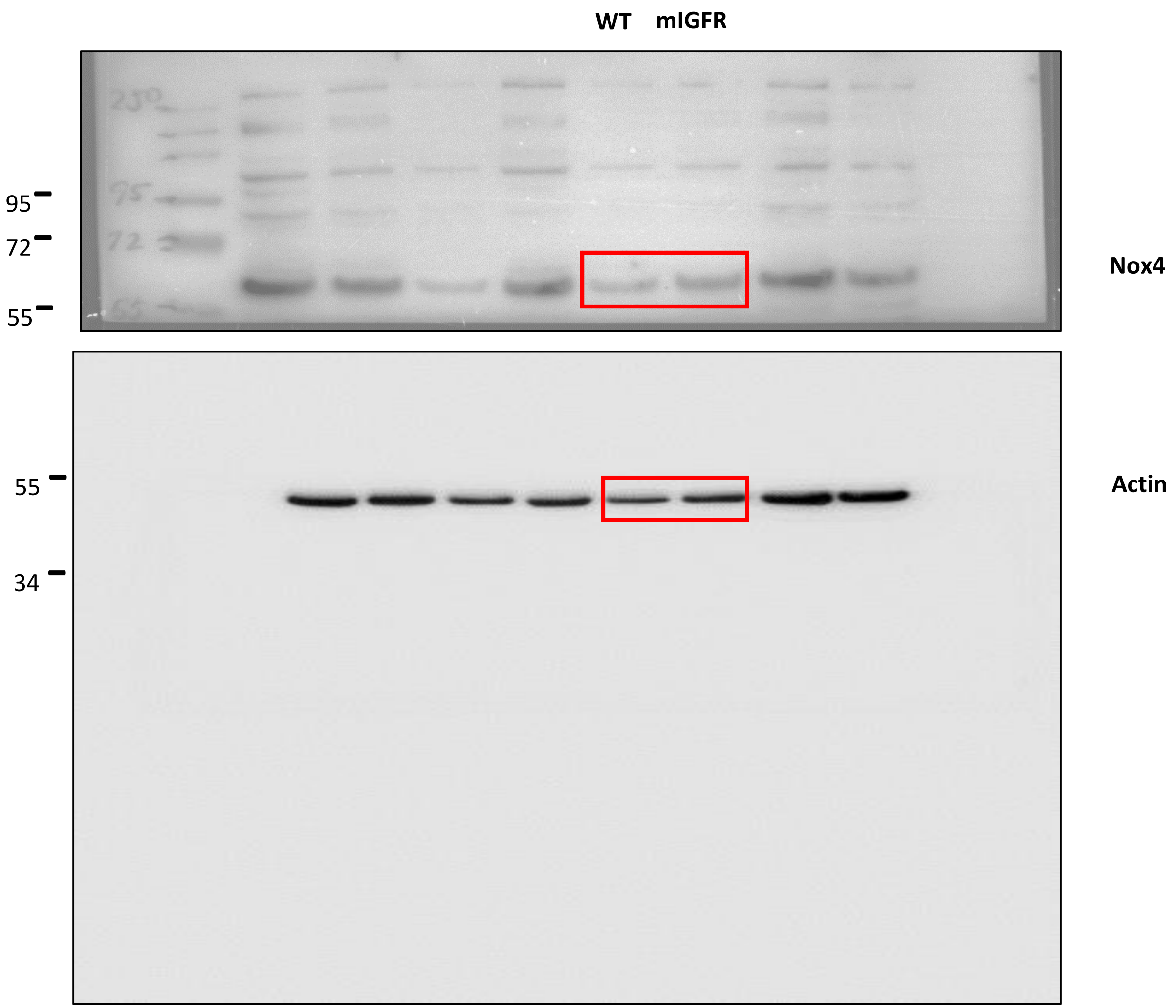

Full unedited gel for Online Figure IIA

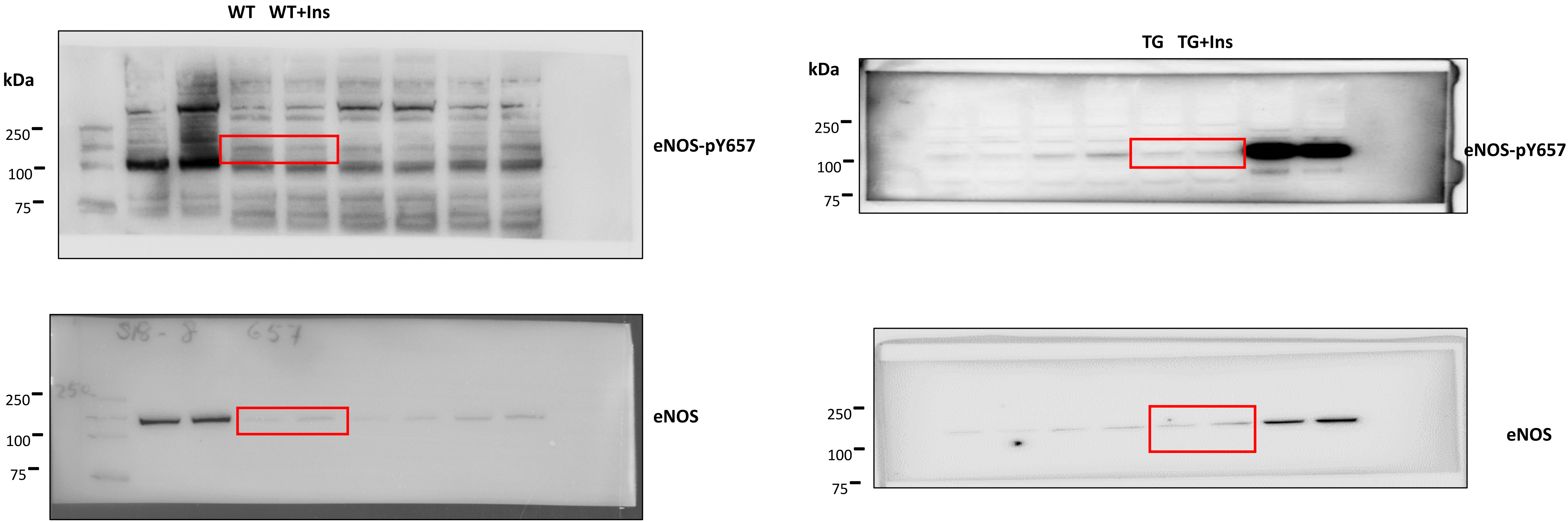

Full unedited gel for Online Figure IIB

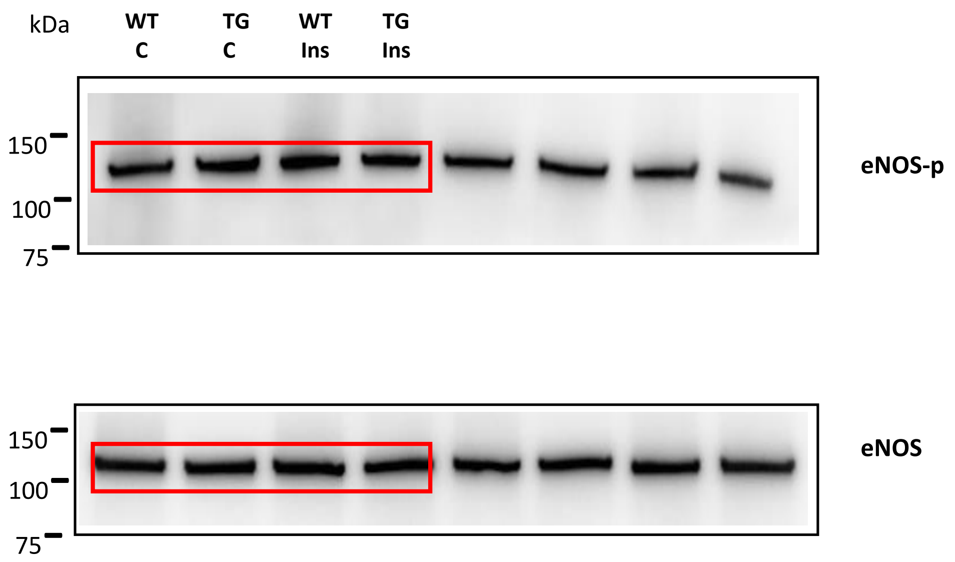

Full unedited gel for Online Figure IIC

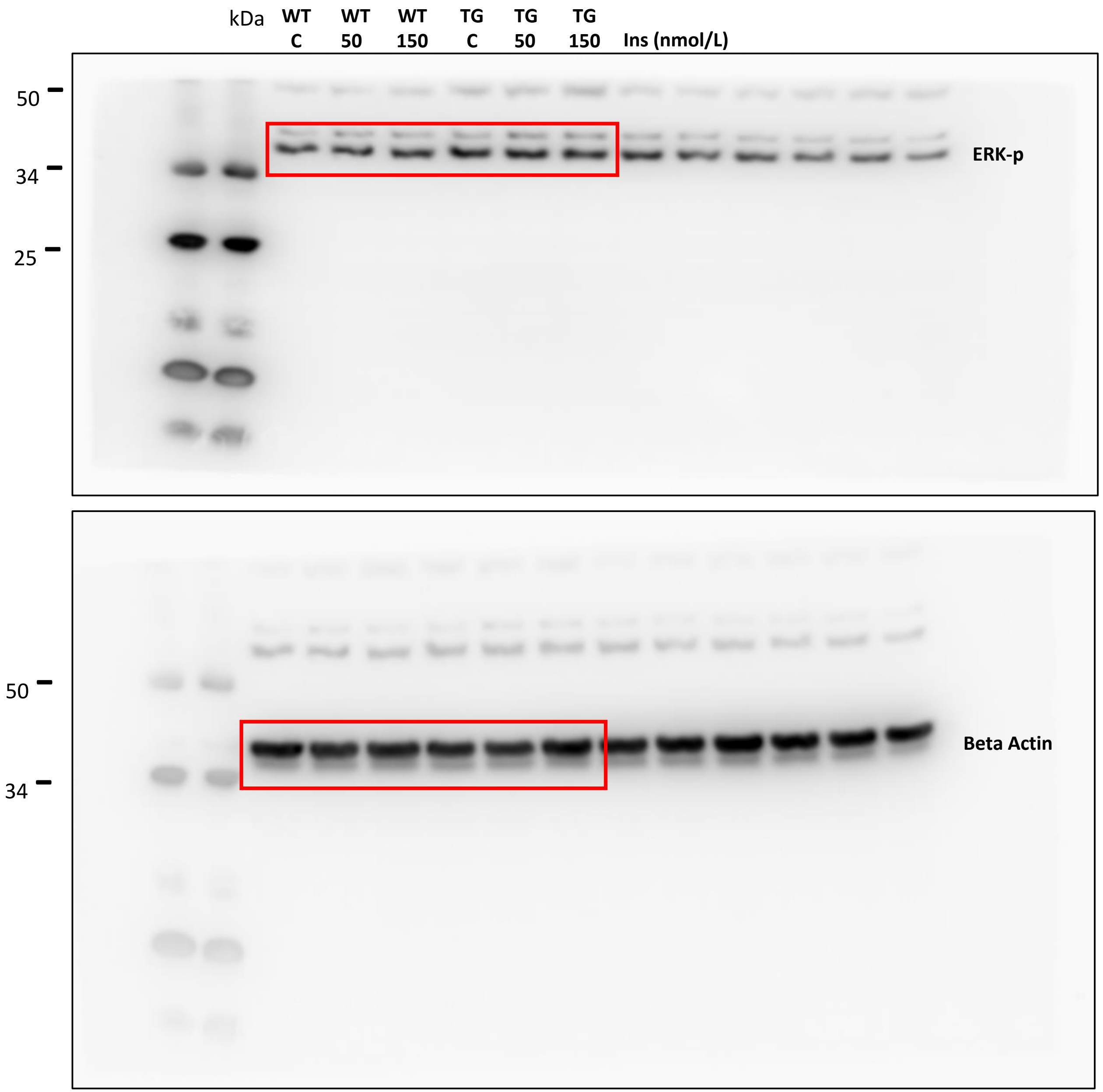

Supplement: Supplementary file 3 [file res-129-720-s003.pdf]
