# Supplementary material for: Novel Paracrine Action of Endothelium Enhances Glucose Uptake in Muscle and Fat
Source: Circ Res. 2021 Aug 20;129(7):720–34. doi: 10.1161/CIRCRESAHA.121.319517 (PMC8448413; doi:10.1161/CIRCRESAHA.121.319517)
Supplement: Supplementary file 4 [file res-129-720-s004.pdf]

# Supplemental Materials

**Novel Paracrine Action of Endothelium enhances Glucose Uptake in Muscle and Fat**

Short Title: Endothelial-specific insulin and IGF-1 resistance

[1]*Hema Viswambharan PhD, [1]*Nadira Yuldasheva PhD, [1]Helen Imrie PhD, [1]Katherine Bridge MBChB, PhD, [1]Natalie Haywood PhD, [1]Anna Skromna MSc, [1]Karen Hemmings PhD, [1]Emily Clark PhD, [1]V Kate Gatenby MBChB, PhD, [1]Paul Cordell PhD, [1]Katie J Simmons PhD, [1]Natallia Makava BSc, [1]Yilizila Abudushalamu MSc, [1]Naima Endesh PhD, [1]Jane Brown BSc, [1]Andrew Walker MBChB, PhD, [1]Simon T Futers PhD, [1]Karen E Porter PhD, [1]Richard M Cubbon PhD, [1]Khalid Naseem BSc, PhD, [2]Ajay M Shah MD, FMedSci, [1]David J Beech PhD, FMedSci, [1]Stephen B Wheatcroft MBChB, PhD, [1]Mark T Kearney DM, FRCP#, [1]Piruthivi Sukumar MBBS, PhD.

[1]Leeds Institute for Cardiovascular and Metabolic Medicine, University of Leeds, United Kingdom. [2] British Heart Foundation Centre of Research Excellence, Kings College London.

#Corresponding Author Prof Mark T Kearney; M.T.Kearney@leeds.ac.uk

Address for Correspondence:
Professor Mark Kearney,
The LIGHT Laboratories,
University of Leeds,
Clarendon Way,
Leeds,
LS2 9JT,
United Kingdom.

*Denotes joint first authors

**Methods: Subject recruitment.** Patients undergoing coronary artery bypass surgery were recruited from the department of cardiac surgery at Leeds Teaching Hospitals and saphenous vein samples obtained as previously described (52). Fifteen male patients with no diabetes, one female with no diabetes, as well as seven male patients with diabetes took part in the study. Ethics approval was granted by the Local Research Ethics Committee (Ref CA01/040).

**Human saphenous vein endothelial cell isolation & culture**. Saphenous vein endothelial cells (SVEC) were isolated using previously described methods (52). Primary SVEC were examined under basal conditions and after stimulation with insulin or IGF-1. SVEC were cultured in 10% FCS-containing media (PromoCells, C-22022) for analysis of protein levels. Insulin or IGF-1 stimulation (Sigma and GroPep, respectively) (100nmol/L; 10 minutes) was performed after overnight serum-deprivation in 0.2% FCS-containing basal media. Experiments were performed on SVECs from a total of 19 patients (out of 23 patients recruited for the study, SVEC were grown successfully only from 19), who were divided in two groups according to diabetic status: group 1: no diabetes (n = 12), aged 69 ± 2.6 years, range 45-79 years; and group 2: type 2 diabetes (n = 7), aged 68.43 ± 3.6 years, range 48–76 years.

**Mice over-expressing mutant IGF-1 receptor specific to the endothelium.** To examine the effects of the combination of insulin and IGF-1 resistance restricted to the endothelium, we generated transgenic mice with endothelial cell-specific over-expression of mIGF-1R (mIGFREO), previously shown to form non-functioning insulin and IGF-1 resistant hybrid receptors with native IR and IGF-1R (11). mIGFREO and wild type littermate (WT) mice were bred onto a C57BL/6J background for at least 12 generations in a conventional animal facility with a 12-h light/dark cycle. Male mice show significantly greater expansion of total body mass including subcutaneous adipose tissue. Since the study focuses on cardiovascular and metabolic changes induced by IGF1R signalling and dietary interventions and also as we are not able to time/control/account for menstrual cycle, we have conducted all the experiments on only the male mice. Male mice aged 12-14 weeks were used in all experiments, which were conducted in accordance with accepted standards of humane animal care under United Kingdom Home Office Project license No. P144DD0D6. All the animals used in the study are bred in-house in our animal facility. Transgenic and wild type littermates are always kept together and the study was carried out blinded to the genotype at the time of investigation. For interventions, we used “simple randomization” method i.e., assigning an animal to each group purely at random for every assignment. No animals were excluded for any experiment.

To overcome the limitations seen in standard transgenics we used the Hypoxanthine phophoribosyl transferase (*Hprt*) targeting system to generate genetically modified embryonic stem (ES) cells (GenOway) (53). This approach uses homologous recombination to target a single copy of a transgene (in this case mutant human IGF-1R) driven by a promoter (in this case the *Tie2* promoter) into the *Hprt* locus on the X chromosome. The model was developed with E15Tg2a (E14) cells derived from the strain 129P2/OiaHsd

(12901a). In E14 cells, 35kb of the *Hprt* gene encompassing the 5' UTR up to intron 2 is deleted. The *Hprt* gene encodes a constitutively expressed housekeeping enzyme involved in purine synthesis from the degradation products of nucleotide bases (salvage pathway). Cells normally synthesise purines by the salvage and *de novo* pathways. In *Hprt* deleted cell lines only the *de novo* pathway is functional, enabling the cells to grow in classical medium. In the presence of the aminopterin drug the *de novo* pathway is blocked, as a result *Hprt* deleted cells die in media called HAT (containing hypoxanthine, aminopterin and thymidine substrates). The targeted insertion of a transgenic cassette in E14 ES cells with a functional *Hprt* gene rescues these cells, which can then be selected using HAT media to identify ES cells showing the correct targeting event. Thus, ES cells with the correct insertion can be selected by virtue of their expression of *Hprt* and the resultant ability to grow in HAT medium. Numerous studies have shown that the functional properties of the *Hprt* locus protect transgenic constructs inserted in this region against gene silencing and positional or methylation effects. Furthermore, tissue-specific promoters (54) inserted into the *Hprt* locus maintain their expression properties.

**Vector construction.** Endothelial cell specific transgene expression was achieved using the mouse *Tie2* promoter and intronic enhancer, as previously described (8). In order to reduce the size of the transgene the minimal-enhancer sequence (core-enhancer) was inserted into the final construct. This sequence has been shown to confer uniform and high level of expression of Lac Z reporter gene in endothelial cells *in vivo* (47). The targeting vector was obtained by inserting human mutant IGF-1R cDNA (a kind gift from Dr R Baserga, Jefferson University USA, (55) with a single amino acid substitution (Lys 1003 to Arg 1003) generated using site directed mutagenesis (Online Figure IA) into the pHHNS plasmid comprising the murine *Tie2* promoter, *LacZ* cDNA, and SV40 polyA signal and a 10-kb intronic enhancer from the murine *Tie2*. The lys 1003 to arg 1003 mutation abolishes ATP binding within the β-subunit of the human IGF-1R cDNA (56). The final transgenic vector consisting of *Tie2* promoter, mutant IGF-1R, polyadenylation site and core-enhancer was then inserted into a *Hprt* targeting vector by a gateway reaction between the transgenic vector and the *Hprt* targeting vector. The final targeting vector had the following features; **1)** Isogenic with E14 ES cells favouring homologous recombination. **2)** Symmetrical homology arms (5' short arms-SA: 3.8kb, 3' long arm –LA: 3.7kb). **3)** Transgenic cassette expressing the human IR cDNA under control of the short form of the Tie2 promoter. **4)** Wild type *Hprt* sequences to reconstitute the *Hprt* gene in the E14 ES cells (Online Figure IB). In ES cells this process was highly successful with 3 clones selected for blastocyst injection. The 5' and 3' targeting events were unambiguously confirmed by Southern blot analysis. These 3 ES clones were expanded and genetically manipulated agouti ES cells were then injected into C57BL/6J derived blastocysts that were then implanted into the uteri of recipient females.

**Breeding of Chimeras and generation of F1 mice.** Five highly chimeric males (displaying 100% chimerism) generated by blastocyst injection of ES clones were mated with 2 wild-type C57BL/6J female mice to examine whether the targeted ES cells contributed to the germ layer. To assess whether ES cells have contributed to

the germ layer of chimeras, mouse coat colour markers are used. The ES cells used to develop the model were originally derived from a strain of mice which have an agouti coat colour. This marker is dominant over the black coat of C57BL/6 mice. Therefore, mating of the chimeras with C57BL/6J mice yields agouti coloured pups when the ES cells have contributed to the germ layer. Four agouti F1 females were genotyped by Southern blot (Online Figure IC). Southern blot validated the correct heterozygous status of tested F1 females by detecting the 7.9kb sized AvrII fragment of the C57BL/6 *Hprt* wild type allele and the 9.8kb sized AvrII fragment of the reconstituted *Hprt* allele (females #1, #2, #3 and #5; see Online Figure ID). Two of the F1 females were backcrossed for at least 12 generations into a C57BL/6J background.
For high fat diet study, mice were placed them on a 60% high-fat diet (HF; diet D12492, Research Diets Inc. New Brunswick, NJ, USA) for 10 days before commencing metabolic experiments.

**Metabolic tests.** Blood was sampled from tail vein. Glucose, insulin and IGF-1 tolerance tests were performed by blood sampling after an intraperitoneal injection of glucose (1mg/g; Sigma Aldrich UK), human recombinant insulin (0.75 unit/kg: Actrapid; Novo Nordisk, Bagsvaerd, Denmark), or human recombinant IGF-1 (0.75μg/g; GroPep, Adelaide Australia), as previously described (5–10). Glucose concentrations were determined in whole blood by a portable meter (Roche Diagnostics, 2 Burgess Hill, UK). Plasma insulin and IGF-1 concentrations were determined by enzyme-linked immunoassay (Ultrasensitive mouse ELISA; CrystalChem, Downers Grove, IL, USA) (8). Free fatty acids levels were determined using a free fatty acid quantification kit (AbCam) and triglycerides concentrations, using a triglyceride quantification kit (AbCam). Adiponectin and leptin were quantified using enzyme-linked immunoassay (EMD Millipore).

**Myeloid cell isolation and IGF-1R expression.** Peripheral blood mononuclear cells were isolated using density-gradient centrifugation (Histopaque 1083; Sigma) of heparinized venous blood collected during terminal anaesthesia (8,9). Peripheral blood mononuclear cells were incubated with CD11b microbeads (Miltenyi Biotec) before undergoing 2 rounds of magnetic purification using MS columns (Miltenyi Biotec), achieving purity exceeding 90%. CD11b+ cells were immediately lysed using TRIzol reagent (Life Technologies), and then, total RNA isolated according the manufacturer's protocol. RNA was then cleaned (RNeasy MinElute; Qiagen), amplified, and reverse transcribed to cDNA (QuantiTect; Qiagen), before real-time quantitative polymerase chain reaction (LightCycler; Roche) using validated TaqMan probes (Life Technologies) for human IGF-1R. All expression was normalized to the cycle threshold of murine β-actin using the $2^{-\Delta CT}$ method. The thermal cycling conditions are as follows: 95C for 10 sec followed by 40 cycles of 95C for 10 sec and 60C for 1 minute.

**Non-invasive measurement of arterial blood pressure.** Systolic and diastolic blood pressure were measured using tail-cuff plethysmography in conscious mice, as previously described (8). Mice were pre-warmed for 10 minutes in a thermostatically controlled restrainer (CODA2 System, XBP1000; Kent

Scientific). Three training sessions were performed during the week before measurements were taken. The mean of at least 5 separate recordings on 3 occasions was taken to calculate mean systolic blood pressure.

***In vivo* hyperinsulinaemic-euglycaemic clamp studies.** *In vivo* euglycaemic insulin clamps were performed at the Mouse Metabolic Phenotyping Centre at Vanderbilt University, as previously described (9,57), with investigators blinded to genotype. *In vivo* **hyperinsulinaemic-euglycaemic clamps** use tracer techniques *to assess: **1)** whole body insulin action, **2)** the ability of insulin to suppress endogenous (i.e., hepatic) glucose production and **3)** rates of glucose uptake by individual tissues including muscle and fat.* Mice were maintained on a chow diet (Harlan Teklad Diet 7012, USA). Catheters were implanted in a carotid artery for blood sampling and jugular vein for infusions 5 days before the study. On the morning of each study, food was removed and clamps initiated after a 5-hour fast. 120 min (t=-120 min) prior to initiation of the clamps, animals received a primed (1.5 µCi), followed by a continuous (0.075 µCi/min) infusion of [3-$^{3}$H] glucose. Baseline blood or plasma parameters were determined in blood samples collected at −10 and 0 min. At *t*=0 an insulin infusion (4 mU/kg/min) was started, the [3-$^{3}$H] glucose infusion rate increased (0.15 µCi/min), and a constant infusion of heparinised saline-washed erythrocytes from donor animals (5.5 µL/min) given to prevent a fall in haematocrit. These infusions were continued for the duration of the clamp (145min). Blood glucose was clamped at ~100-110mg/dL using a variable rate of glucose infusion (GIR). Insulin clamps are validated by assessment of blood glucose over time. Blood glucose was monitored every 10 min, and GIR adjusted accordingly. Blood was taken between 80–120 min for determination of [3-$^{3}$H] glucose. Clamp insulin was determined at *t*=120 and 145min. At 120min 13 µCi of 2[$^{14}$C] deoxyglucose ([$^{14}$C]2DG) was administered as an intravenous bolus. Blood was taken between 122–145 min for the determination of [$^{14}$C] 2DG. After the last sample, mice were euthanized and tissues collected.

**Plasma and muscle sample analysis post hyperinsulinaemic-euglycaemic clamp.** Immunoreactive insulin was assayed using a rat radioimmunoassay kit (Millipore, Billerica, MA). To measure 3-[$^{3}$H]-D-glucose plasma the sample was deproteinized with barium hydroxide ($Ba(OH)_2$) and zinc sulfate ($ZnSO_4$), dried, and radioactivity determined using liquid scintillation counting. Excised soleus, gastrocnemius, superficial vastus lateralis and gonadal brown adipose tissue, were deproteinized with perchloric acid and then neutralised to a pH of ~7.5. A portion of the extract was counted ([2-$^{14}$C]DG and [2-$^{14}$C]DG-G-phosphate ([2-$^{14}$C]DGP) and a portion treated with $Ba(OH)_2$ and $ZnSO_4$ and the supernatant counted ([2-$^{14}$C]DG). Both [2-$^{14}$C]DG and [2-$^{14}$C]DG-G-phosphate ([2-$^{14}$C]DGP) radioactivity levels were determined using liquid scintillation counting.

**Studies of vasomotor function in aortic rings.** Vasomotor function was assessed in aortic rings as previously described (5–7,9). Rings were mounted in an organ bath (PanLab Multi-chamber) containing Krebs-Henseleit buffer (reagents from ThermoFisher and Sigma) and equilibrated at a resting tension of 3g for 45min before the experiments. A cumulative dose response to the constrictor phenylephrine (PE, 1nmol/L to 10µmol/L), was first performed. Relaxation responses to cumulative addition of acetylcholine (Ach, 1nmol/L-10µmol/l) and sodium nitroprusside (SNP, 0.1nmol/L-1µmol/L), were performed and responses

expressed as % decrement in pre-constricted tension. Basal NO bioavailability in response to isometric tension was measured by recording the increase in tension elicited by L-NMMA (Sigma, 0.1mmol/L; 30 minutes), in aortic segments maximally pre-constricted with PE. To examine the possibility that $H_2O_2$ may contribute to vasomotor tone we performed ACh relaxation responses after exposure to the $H_2O_2$ dismutase, catalase (1250 U/mL, 5 minutes; Sigma) (56) or catalase inhibitor 3-Amino-1,2,4 triazole (Sigma, 10mmol/L, 30 minutes). Ach (1nmol/L to 0.01 mmol/L) relaxation responses were performed after exposure to amino triazole, catalase inhibitor 30 minutes prior to catalase treatment. We also compared area under curves (AUC), as well as $EC_{50}$ where appropriate.

**Studies of vasomotor function in mesenteric artery.** Segments of second-order mesenteric arteries were harvested from WT and mIGFREO mice and mounted in a wire myograph system (Multi Wire Myograph System, 620 M), containing Krebs solution at 37°C, 5% $CO_2$ and 95% $O_2$. Vessels were equilibrated at a resting lumen diameter of 0.9×L100 (L100 represents vessel diameter under passive transmural pressure of 100mmHg), then, two potassium-induced constrictions were performed using high potassium solution. Then, vessels were pre-constricted with phenylephrine, at a dose yielding approximately 40% constriction obtained with high potassium solution, and after stabilisation period, relaxation to cumulative addition of insulin (0.1nM to 0.01 mM) was assessed in pre-constricted vessels. Arteries were only used for investigation if they constricted in response to phenylephrine (PE) and dilated in response to ACh.

**Mouse Pulmonary endothelial cell isolation and culture.** Primary endothelial cells were isolated from lungs by immunoselection with CD146-antibody-coated magnetic beads (Miltenyi Biotech), as previously reported (8,9). Sheep anti-rat-IgG magnetic beads were coated with monoclonal rat anti-CD31 antibody (BD Biosciences), according to the manufacturer's instructions. Lungs were harvested, washed, finely minced and digested in HBSS (Gibco) containing 0.18U/mL collagenase type 2; 10mg/mL (Worthington Biochemical Corporation, NC9693955), and for 45 minutes at 37°C. The digested tissue was filtered through a 70μm cell strainer and centrifuged at 1000 rpm for 10 minutes. The cell pellet was washed with DMEM/10% FCS, centrifuged, resuspended in 1 ml DMEM/10%FCS and incubated with $1x10^6$ CD31-Ab-coated beads at 4°C for 30 minutes. Bead-bound cells were separated from non-bead bound cells using a magnet. Bead-bound (CD31 positive), and non-bead-bound cells were resuspended in 2 ml EGM-2-MV (Cambrex), supplemented with hEGF, hydrocortisone, VEGF, hFGF-B, R3-IGF-1, ascorbic acid, gentamicin, amphotericin-B and 5% FCS and plated out. Only the endothelial cell population tested positive for a range of endothelial cell markers including eNOS, *Tie2* and CD102 protein measured using immunoblotting. These are the pulmonary endothelial cells (PECs). Non-endothelial cells were the cell fractions in the eluates from the magnetic columns (Miltenyi Biotech), upon removal of endothelial fraction and cultured for experiments using MV2 medium containing antifungal-antimicrobial agents and MV2 supplements (Promocell).

**Nitric oxide synthase activity.** eNOS activity in endothelial cells was determined by conversion of [$^{14}$C]-L-arginine to [$^{14}$C]-L-citrulline as we previously described (5). Endothelial cells grown to 80% confluence (1x$10^5$ per well in a 6-well plate), were incubated at 37°C for 20 min in HEPES buffer pH 7.4 (in mmol/L): 10 HEPES, 145 NaCl, 5 KCl, 1 MgSO4, 10 glucose, 1.5 $CaCl_2$ containing 0.25% BSA. 0.5 µCi/ml L- [$^{14}$C] arginine was then added for 5 min and endothelial cells stimulated with, insulin (150nmol/L) or IGF-1 (150nmol/L) for 30 minutes, before the reaction was stopped with cold phosphate-buffered saline (PBS), containing 5 mmol/L L-arginine (Sigma) and 4 mmol/L EDTA (Sigma) after which tissue was denatured in 95% ethanol. After evaporation, the soluble cellular components were dissolved in 20 mmol/L HEPES-$Na^+$ (pH 5.5), and applied to a well-equilibrated DOWEX (Sigma; $Na^+$ form) column. The L-[$^{14}$C] citrulline content of the eluate was quantified by liquid scintillation counting and normalized against the total cellular protein.

**Immunoblotting.** Primary cells were lysed in extraction buffer containing (in mmol/L), 50 HEPES, 120 NaCl, 1 $MgCl_2$, 1 $CaCl_2$, 10 $NaP_2O_7$, 20 NaF, 1 EDTA, 10% glycerol, 1% NP40, 2 sodium orthovanadate, 0.5µg/mL leupeptin, 0.2 PMSF, and 0.5µg/mL aprotinin (Invitrogen Cell Extraction Buffer, FNN00 11). Cell extracts were incubated for 30 minutes in an ice-bath and centrifuged at 14,000 rpm for 15 minutes, before protein measurements were carried out by BCA assay kit (Pierce), using the supernatant (8). For the analysis of total protein expression, equal amounts of cellular protein were resolved on SDS polyacrylamide gels (Invitrogen) and transferred to polyvinylidene difluoride membranes. Membranes were then, immunoblotted with appropriate antibodies (Online Table I; eNOS Ser-1177, phosphorylated ERK and ERK from Cell Signaling. Beta-actin from Santa Cruz Biotechnology. NOX2 and NOX4 antibodies' details are provided in Supplemental Materials). We have previously employed the Nox2 antibody and demonstrated its' specificity using siRNA (7). The Nox4 antibody has been validated using shRNA (57) and knockout (58). Blots were incubated with appropriate peroxidise-conjugated secondary antibodies and developed with enhanced chemiluminescence imaging kit (Millipore). Levels of protein were quantified using densitometry (Syngene Gel Documentation) based on the intensity of bands from each sample. All bands density quantification for blots have been normalised against Beta-actin or tubulin as loading controls. All the blots were repeated at least 5 times with independent samples, using identical experimental conditions. Representative blots were selected that is the nearest representation to the mean data presented which supports the conclusions.

**Chronic treatment with catalase.** To examine the effect of dismutation of hydrogen peroxide ($H_2O_2$) to water and oxygen on insulin tolerance in mIGFREO mice, we performed chronic treatment studies using PEG-catalase. mIGFREO mice and wild type littermates were anesthetized, and osmotic mini-pumps (Alzet 2004) containing catalase implanted, as previously reported (5). The pump delivered 10,000U/kg/day of PEG-catalase (Sigma) daily (59) and insulin tolerance testing was performed after 5-days' treatment.

**Immunoprecipitation.** Tyrosine phosphorylation of the insulin receptor in liver and muscle was quantified using Western blotting. Immunoprecipitation of protein for the quantification of receptors was achieved by incubating equal amounts of protein lysates (200 µg) and 30 µL of protein A-Agarose beads (Roche) pre-

coated with indicated rabbit anti-insulin receptor antibody for 3 hours at 4ºC. After 5 rounds of washing with PBS-0.01%Tween-20, the beads were resuspended in 2x Laemmli loading buffer supplied with the NOVEX Gel Electrophoresis system. Cellular protein was separated by SDS-PAGE and blots were probed with a mouse phospho-tyrosine 4G10 (Millipore). For hybrid receptor or activated hybrid receptor analysis, samples were immunoprecipitated, as above using IR-beta antibody and probed with IGF-1R antibody (D23H3 or IR-pY1334 from Cell Signaling, respectively). Equal loading was confirmed using protein quantification and beta-actin or tubulin antibodies.

**Gene expression and miR-25 studies.** mRNA was isolated using a Trizol (Invitrogen) method, and reverse transcribed to cDNA (Primer Design, UK), before quantification of IGF-1R, IR, SOD 1-3, catalase, Nox2, and Nox4 mRNA by real-time polymerase chain reaction (LightCycler; Roche) using validated SYBR Green probes (Life Technologies; Online Table II). All expression was normalized to the cycle threshold of murine β-actin using the $2^{-\Delta CT}$ method. For miR-25 expression, specific Taqman assays were obtained (AB Systems; U6 snRNA; Part number: VY4427975 (both mouse and human-house keeper miRNA), mmu-miR-25-3p; Part number: 4427975 (for mouse); hsa-miR-25-3p; Part number: 4427975 (for human)) and PCR was performed in AB Systems 7500 real time machine. The miR reverse transcription conditions are: 16°C for 30 minutes, 42°C for 30 minutes followed by 85°C for 5 minutes and then held at 4°C. The thermal cycling conditions are as follows: 95C for 10 sec followed by 40 cycles of 95C for 10 sec and 60C for 1 minute. Pre-miR miRNA Precursors (miR-25 mimetic; Ambion; Part number: 4457175) was transfected into endothelial cells using Lipofectamine (AB Systems) following manufacturer's protocol and Nox4 expression was measured after 48-72 hours.

**Lucigenin enhanced chemiluminescence.** We used lucigenin (5µM) enhanced chemiluminescence to measure NAD(P)H-dependent superoxide production in endothelial cells as previously described (6–8). All experiments were performed in triplicate. Pulmonary endothelial cell monolayer was trypsinised and re-suspended in PBS containing 5% FCS, 0.5% BSA and 50uM gp91ds-tat (GenScript) or scrambled peptide (GenScript) and incubated in 37˚C for 30 min. Then luminescence was measured upon addition of a non-redox cycling concentration of lucigenin (5µM) and NADPH (100µM), using an auto dispenser (VarioSkan or Clariostar 96-well microplate luminometer, Thermo Scientific).

**Amplex red assay for hydrogen peroxide in aorta.** $H_2O_2$ was measured using an Amplex® Red Hydrogen Peroxide/Peroxidase Assay Kit (Molecular Probes, Life Technologies) as described (8,9). Freshly harvested aortas were collected into a modified Krebs-HEPES buffer, containing 20 mM HEPES, 119 mM NaCl, 4.6 mM KCl, 1 mM $MgSO_4.7H_2O$, 0.15 mM $Na_2HPO_4$, 0.4 mM $KH_2PO_4$, 5 mM $NaHCO_3$, 1.2 mM $CaCl_2$ and 5.5 mM glucose, pH 7.4. The aortas were cleaned of peripheral adipose tissue and divided in about 2mm aortic rings. Half of the aortic rings were incubated in 50µL modified Krebs-HEPES buffer and the remaining aortic rings were incubated in 50µL of the modified Krebs-HEPES buffer with 1250 U/mL catalase (free from tymol) for 1h at 37°C. Fifty microlitres of freshly-prepared 100 µM Amplex Red reagent with 0.2U/mL HRP was

added to the samples and incubated for 1 hour at 37°C, protected from light. The aortic rings were removed from the samples and fluorescence was measured on VarioSkan (Thermo Scientific) plate reader (excitation 530 nm and emission 590 nm). The average readings with catalase were subtracted from average readings without catalase, and the value was used as input into an $H_2O_2$ standard curve. The $H_2O_2$ standard curve was prepared in the same plate simultaneously, with the tissues and was used to determine $H_2O_2$ concentration released in the samples. Weight of blotted dry tissue was used for normalization.

**Statistics.** A priori sample size calculations for animal experiments were performed using our published pilot data using the online software package from Vanderbilt University for multiple types of power analysis (https://biostat.app.vumc.org/wiki/Main/PowerSampleSize; 6-8). Results are expressed as mean±standard error of mean (SEM). We used histogram plotting to examine data distribution and using Shapiro-Wilks test for normality, P>0.05 threshold for normal distribution. We used parametric testing for normally distributed data and non-parametric (t-test with ad hoc corrections) for non-normally distributed data. Analysis performed using GraphPad Prism 8.00 (GraphPad Software Inc) and OriginPro 2019b (OriginLab Corporation). Comparisons between groups were performed using unpaired Student t tests or repeated-measures ANOVA, as appropriate; For correction of multiple comparisons, Bonferroni (for repeated measurements) or Fisher's test (for non-repeated measurements) were used. A p value <0.05 was considered statistically significant.

## Supplemental Table and Figure Legends

**Online Table I.** Table showing sources, catalogue codes and dilutions of antibodies used in the study. We have previously validated the fidelity of antibodies to Nox2 and Nox4 in tissues and cells from mice with germline deletion of Nox2 or Nox4 and using RNAi knockdown approach (7,10&14).

**Online Table II.** Table showing different gene specific primers used in real-time RT-PCR.

**Online Figure I.** Scheme showing the steps and constructs used for the generation of mIGFREO line **(A)**. Photograph of (a, 1 and 2) wild type and (b, 3 and 4) mIGFREO mice shows no difference in size or gross features (**B)**. Southern blot validated the correct heterozygous status of tested F1 females by detecting the 7.9kb sized AvrII fragment of the C57BL/6 *Hprt* wild type allele and the 9.8kb sized AvrII fragment of the reconstituted *Hprt* allele females (#1, #2, #3, #5 - mIGFREO; WT - wild type) (**C)**. Fat pad weights of mIGFREO and wild type control mice (**D;** WT n=5, mIGFREO n=5). Systolic and diastolic blood pressure of wild type and mIGFREO mice (**E;** WT n=10, mIGFREO n=10). Endogenous mouse IGF-1R mRNA expression in aorta and lungs of mIGFREO and control animals (**F;** WT aorta and mIGFREO aorta n=11; WT lungs n=10, mIGFREO lungs n=12). Mutant human IGF-1R mRNA expression in aorta and monocytes isolated from mIGFREO and control animals (**G;** WT n=4, mIGFREO n=4). Mutant human IGF-1R mRNA (mIGF-1R) expression in pancreatic islets, denuded aorta, pericytes and endothelial cells (PECs; described

in Methods) isolated from mIGFREO animals (**H;** Islet, Denuded Aorta and Pericytes n=4, PECs n=3). Data expressed as mean±sem. * denotes p<0.05 WT vs mIGFREO or cell types. Data were analysed by unpaired Student t test.

**Online Figure II.** eNOS phosphorylation (endothelial p-Y657; **A;** WT n=5, mIGFREO n=5 and **B;** aortic p-S1177; WT n=5, mIGFREO n=5) in mIGFREO and WT littermates in the presence of insulin (0.75U/kg; 10 minutes). Dose-dependent insulin-stimulation of (50nM and 150nM; 10min) total ERK phosphorylation in WT and mIGFREO endothelial cells (**C;** WT n=5, mIGFREO n=8). PKC activity levels in wild type and mIGFREO endothelial cells stimulated with Insulin (Ins; 150nM; 10min) **D;** WT n=4, mIGFREO n=4). *WT denotes wild type mice and mIGFREO / TG denotes mutant human IGF1-R transgenic mice*. Data expressed as mean±sem. In Figure **B**, * denotes p<0.05 WT control vs WT+Insulin. In Figure **D**, * denotes p<0.05, WT+Insulin vs mIGFREO+Insulin. Data in **A, B** were analysed by ANOVA; Fisher's Multiple comparisons tests, **C** were analysed by were analysed by multiple t tests and **D** by unpaired Student T test; Mann Whitney test .

**Online Figure III.** Random blood IGF-1 levels in mIGFREO and control littermates in fed state (**A;** WT n=30, mIGFREO n=30). Mean EC50 for insulin-induced vasorelaxation in 2$^{nd}$ order mesenteric vessels of WT and mIGFREO (**B**; WT n=6, mIGFREO n=5). Mean serum leptin (**C**; WT n=18, mIGFREO n=18) and adiponectin (**D**; WT n=19, mIGFREO n=19) levels in mIGFREO mice and WT littermates. Data expressed as mean±sem. In Figure **B** * denotes p<0.05, WT vs mIGFREO. Data were analysed by unpaired Student t test.

**Online Figure IV. (A-C)** Data from low-dose hyperinsulinaemic euglycaemic clamp studies on mIGFREO and control littermate animals showing fasting (basal) glucose turnover (**A**; WT n=8, mIGFREO n=8) and endogenous glucose production (EndoRa) (**B**; WT Basal n=9, WT Clamp n=7; mIGFREO Basal and mIGFREO Clamp n=9) and glucose uptake into soleus muscle in mIGFREO and WT littermates (**C**; WT n=5, mIGFREO n=8). * denotes p<0.05, WT vs mIGFREO. Data expressed as mean±sem. Data in **A** and **B** were analysed by unpaired Student t test. Data in C were analysed by unpaired Student t test, Mann Whitney test.

**Online Figure V. (A-E)** Endothelial-dependent vasorelaxation to acetylcholine (Ach) in aortic ring from mIGFREO and WT littermates (WT) (**A**; n=8-12 animals, WT n=49, mIGFREO n=36 rings). Vasoconstrictor response to phenylephrine (PE) without the NO synthase inhibitor, L-NMMA (**B;** n=8-12 animals, WT n=49, mIGFREO n=36 rings) and in the presence of L-NMMA (**C;** WT n=10, mIGFREO n=7) in aortic rings from mIGFREO and WT littermate animals. Vasorelaxation response to Ach after catalase treatment (catalase; 10,000U/kg/day) for 7 days in WT (**D;** WT n=22, WT+catalase; n=22) and mIGFREO (**E;** mIGFREO+Catalase; n=30, mIGFREO n=30). **(F&G)** Combined graph for $LogEC_{50}$ **(F)** and change in maximum relaxation (Emax; **G**) to Ach after catalase treatment in mIGFREO and WT. **(H)** Ach-vasorelaxation curve of WT and mIGFREO aortae in the presence of catalase (left) and catalase + catalase inhibitor (right;

3-amino-1,2,4-triazole, 10mmol/L 30 minutes; n=6 animals; WT n=18 rings, mIGFREO n=20 rings). **(I&J)** Catalase-inhibitable levels of $H_2O_2$ in mIGFREO and WT aorta (**I;** n=7) and superoxide levels in PEC from mIGFREO and WT (**J;** n=6). Data expressed as mean±sem. In Figure **D** * denotes p<0.05 WT vs WT+Catalase. In Figure **E** * denotes p<0.05 mIGFREO vs mIGFREO+Catalase. In Figure **F**, * denotes p<0.05 WT vs WT+catalase and $^{\#}$ denotes p<0.05 mIGFREO vs mIGFREO+catalase. In Figure **G** and **H** * denotes p<0.05 WT+catalase vs mIGFREO+Catalase. In Figure **I** * denotes p<0.05 WT+Catalase vs mIGFREO+Catalase. Data in **G, I & J** were analysed using unpaired Student t test. Data in **F** analysed using One way ANOVA, Fisher's multiple comparisons test. Others were analysed by 2-way ANOVA, followed by Bonferroni's multiple comparisons test.

**Online Figure VI.** Endothelial-independent vasorelaxation to sodium nitroprusside (**A;** SNP, WT n=22, mIGFREO n=29 rings). Relative mRNA expression of ROS-related genes in endothelial cells of mIGFREO and WT littermates (**B;** WT n=5, mIGFREO n=5). Data expressed as mean±sem. Data in **A** were analysed by One-way ANOVA, followed by Bonferroni's multiple comparison test. Data in **B** were analysed by unpaired Student t test.

**Online Figure VII.** Changes in body weight before and after 60% high fat diet-feeding (HFD) of WT and mIGFREO mice for 10 days (**A,** WT n=8, mIGFREO n=12). Fasting glucose levels in wild type and mIGFREO mice after high fat feeding (**B**; WT n=8, mIGFREO n=12). Glucose tolerance tests **(C, left** WT n=8, mIGFREO n=12**)** and Area Under Curve for GTT **(C, right** WT n=8, mIGFREO n=12**)** after high fat diet feeding of wild type and mIGFREO mice and glucose levels 30 minutes' post-glucose tolerance tests (**D**; WT n=8, mIGFREO n=12) in WT and mIGFREO fed with high fat feeding. Insulin tolerance tests **(E, left)** and Area Under Curve for ITT **(E, right)** after high fat feeding of wild type and mIGFREO mice (WT n=8, mIGFREO n=12). Data expressed as mean±sem. Data in **A**, **B, C right panel, D and E, right panel** were analysed by unpaired Student t test. Others were analysed by one-way ANOVA with Bonferonni's multiple comparison.

**Online Table I: Sources of antibodies used**

| Antibody | Dilution | Catalogue Code |
|---|---|---|
| eNOS | 1 in 1000 | BD Bioscience;Mouse Cat # 610297 |
| eNOS-ser1177 | 1 in 1000 | Cell Signaling; Rabbit mAb #9570 |
| Akt | 1 in 1000 | Cell Signaling; Mouse #2920 |
| Akt-ser473 | 1 in 1000 | Cell Signaling; XP® Rabbit mAb #4060 |
| ERK-p | 1 in 1000 | Cell Signaling; XP® Rabbit mAb #4370 |
| Tubulin | 1 in 1000 | Santa Cruz Biotechnologies; Mouse sc-73242 |
| Actin | 1 in 3000 | Santa Cruz Biotechnologies; Mouse sc-47778 |
| Nox2 | 1 in 1000 | BD Biosciences; Mouse 611415 and AbCam; Rabbit ab129068 |
| Nox4 | 1 in 1000 | AbCam; Rabbit ab133303 and Rabbit antibody (ref no 14) |
| Ins Receptor | 1 in 100 | Cell Signaling; 4B8 Rabbit mAb #3025 |
| IGF1 receptor | 1 in 100 | Cell Signaling; XP® Rabbit mAb #9750 |
| IR-pY1334 | 1 in 1000 | Invitrogen; Phospho-INSR (Tyr1334) 44-809G |
| Phospho-tyrosine | 1 in 1000 | Millipore; 4G10® Platinum, Anti-Phosphotyrosine |
| HRP-conjugated secondary | 1 in 5000 | Invitrogen; # 31450 and # 31460 |

**Online Table II: mRNA PCR Primers**

| Gene | Forward (5'-3') | Reverse(5'-3') |
|---|---|---|
| Beta - Actin | CGTGAAAAGATGACCCAGATCA | TGGTACGACCAGAGGCATACAG |
| mNox2 | GGTTCCAGTGCGTGTTGCT | GCGGTGTGCAGTGCTATCAT |
| mNox4 | GGAGACTGGACAGAACGATTCC | TGTATAACTTAGGGTAATTTCTAGAGTGAATGA |
| hIGF1R | CCAGGCC AAAACAGGATATGAA | TCTCTTTCTATGGAAGACGTACAGCAT |
| mIGF1R | ACCGTCTAAACCCAGGGAACTAT | CTCATACGTCGTTTTGGCGG |
| mNox1 | TCGACACACAGGAATCAGGA | TTACACGAGAGAAATTCTTGGG |
| mNox3 | CCGGCAGATCCAATAGAAGT | GTCACTCCCTTCGCCTCTCT |
| mSOD1 | GGACCTCATTTTAATCCTCACTCTAAG | GGTCTCCAACATGCCTCTCTTC |
| mSOD2 | CACACATTAACGCGCAGATCA | GGTGGCGTTGAGATTGTTCA |
| mSOD3 | GGGATGGATCTAGAGCATTAAGGA | ACACCTTAGTTAACCCAGAAATCTTTTC |

Online Figure I

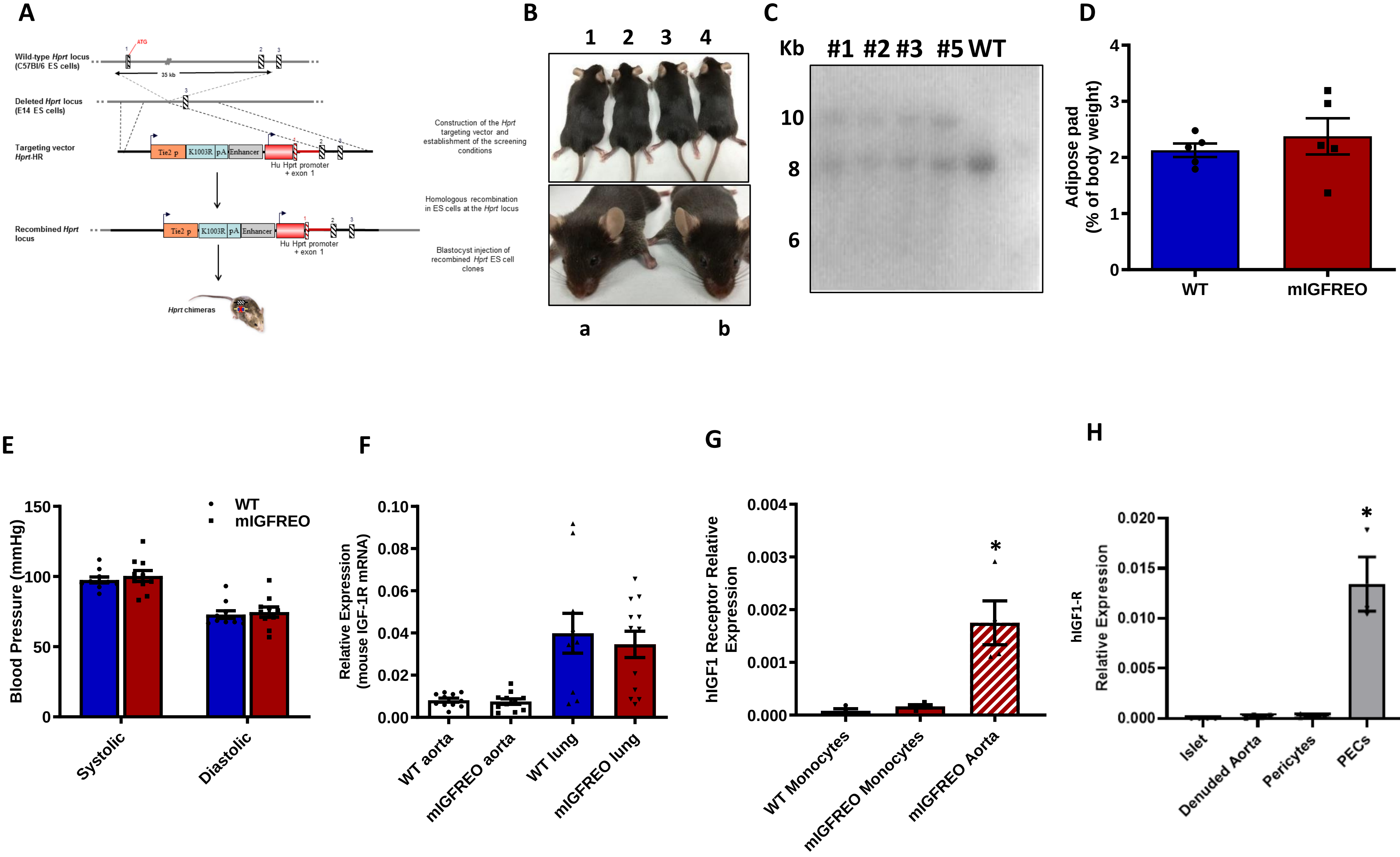

Online Figure II

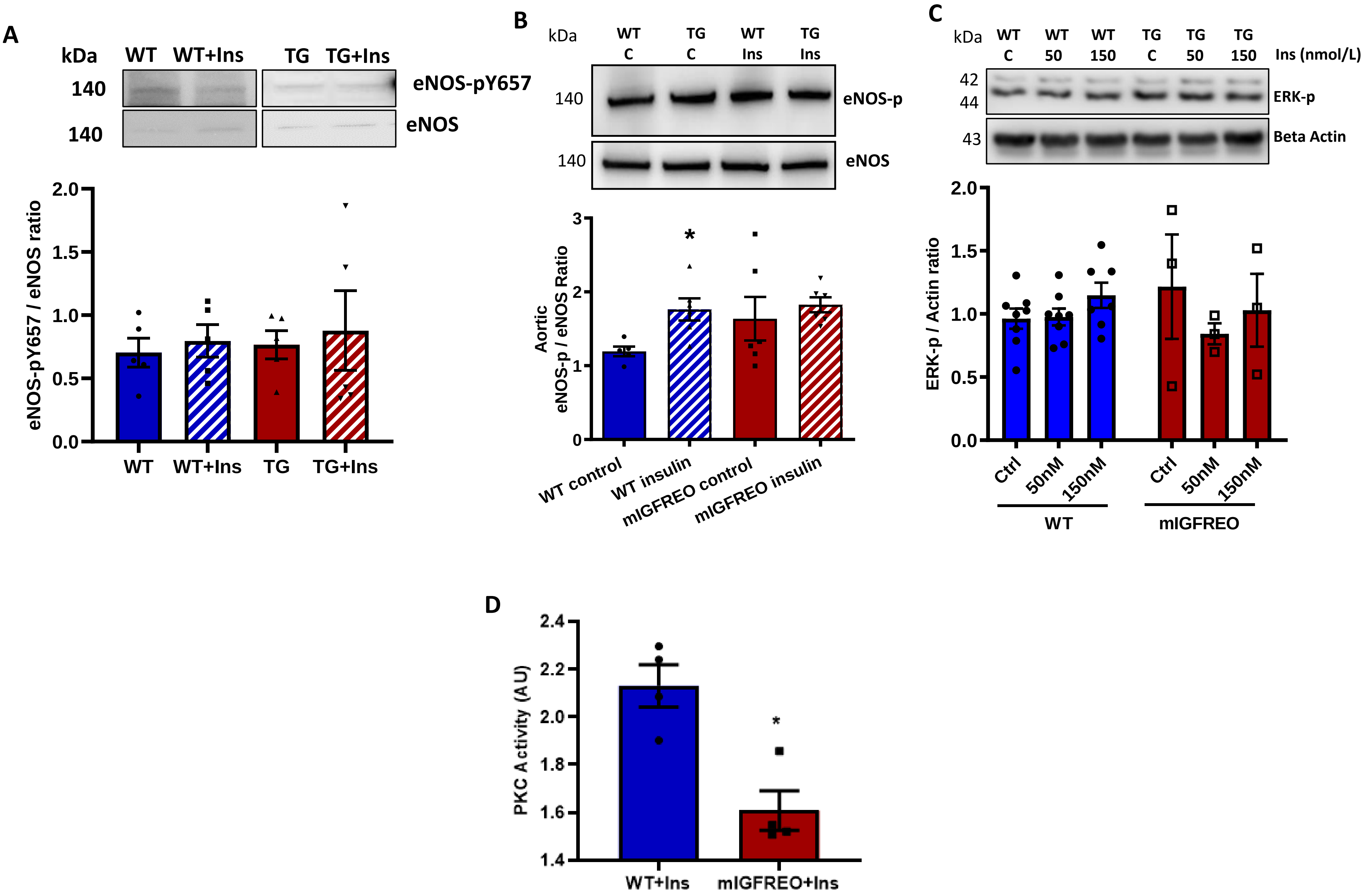

Online Figure III

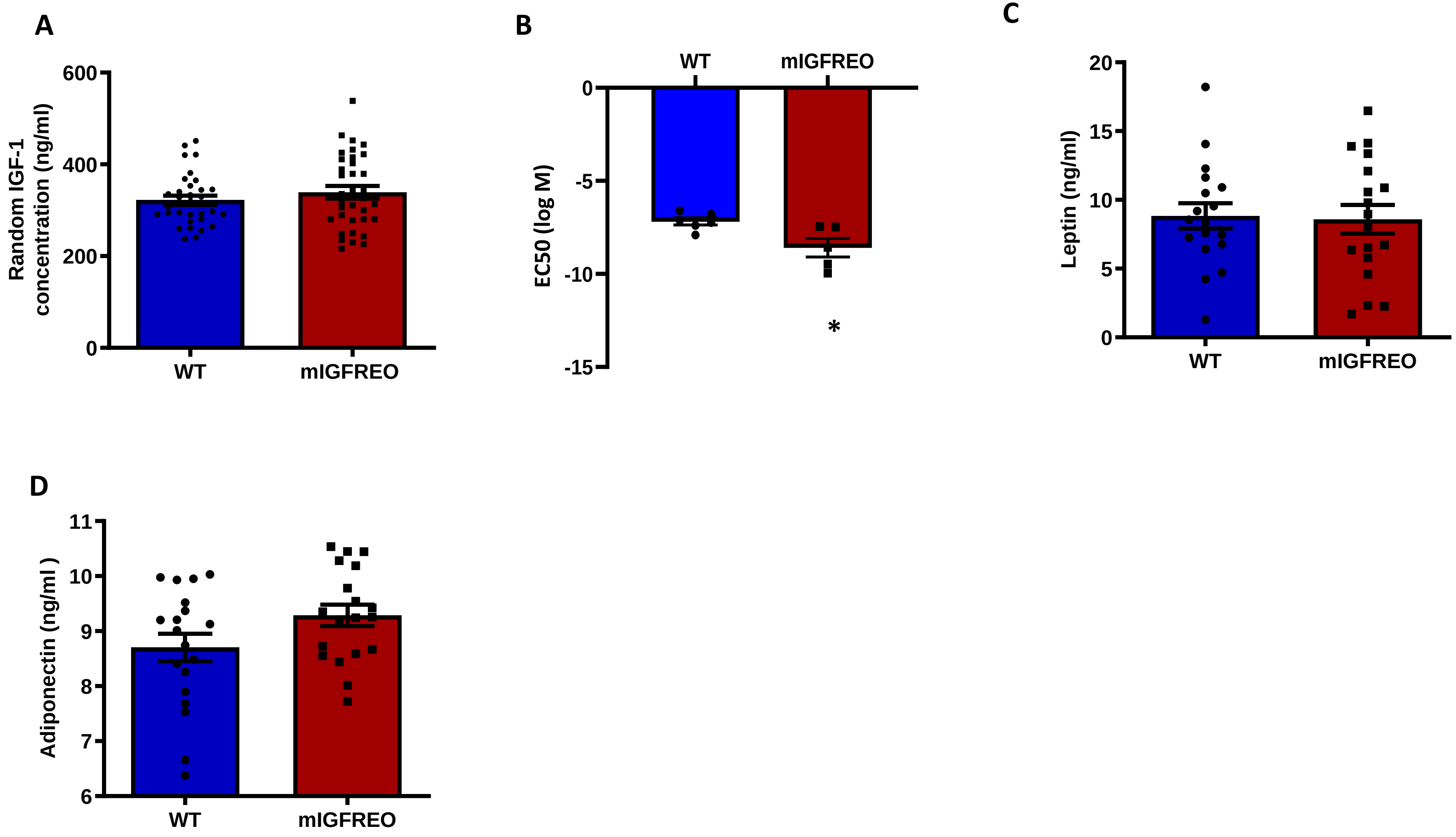

Online Figure IV

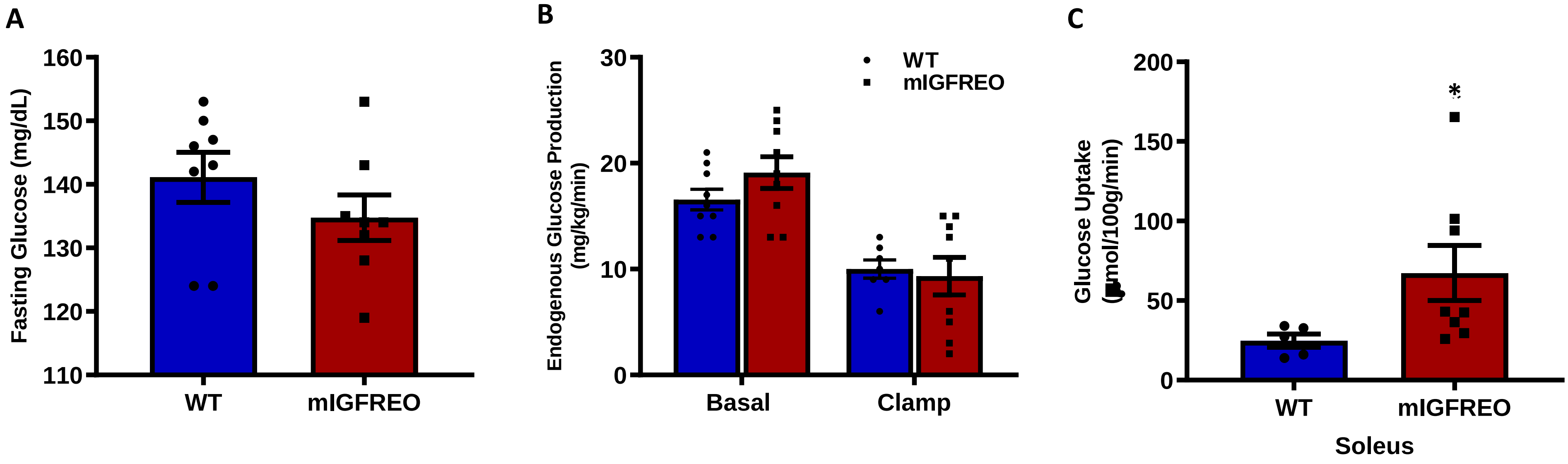

**Online Figure V**

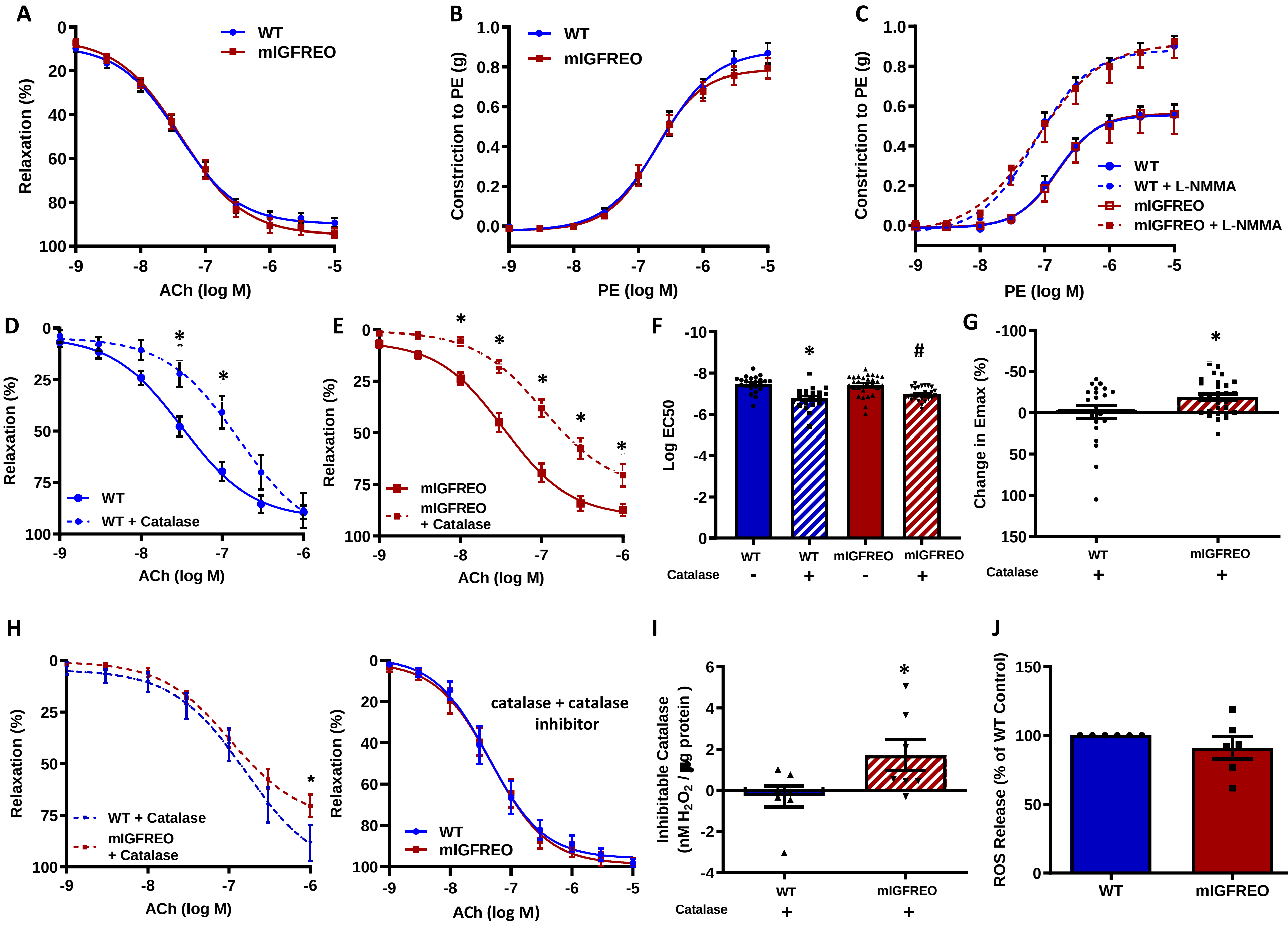

Online Figure VI

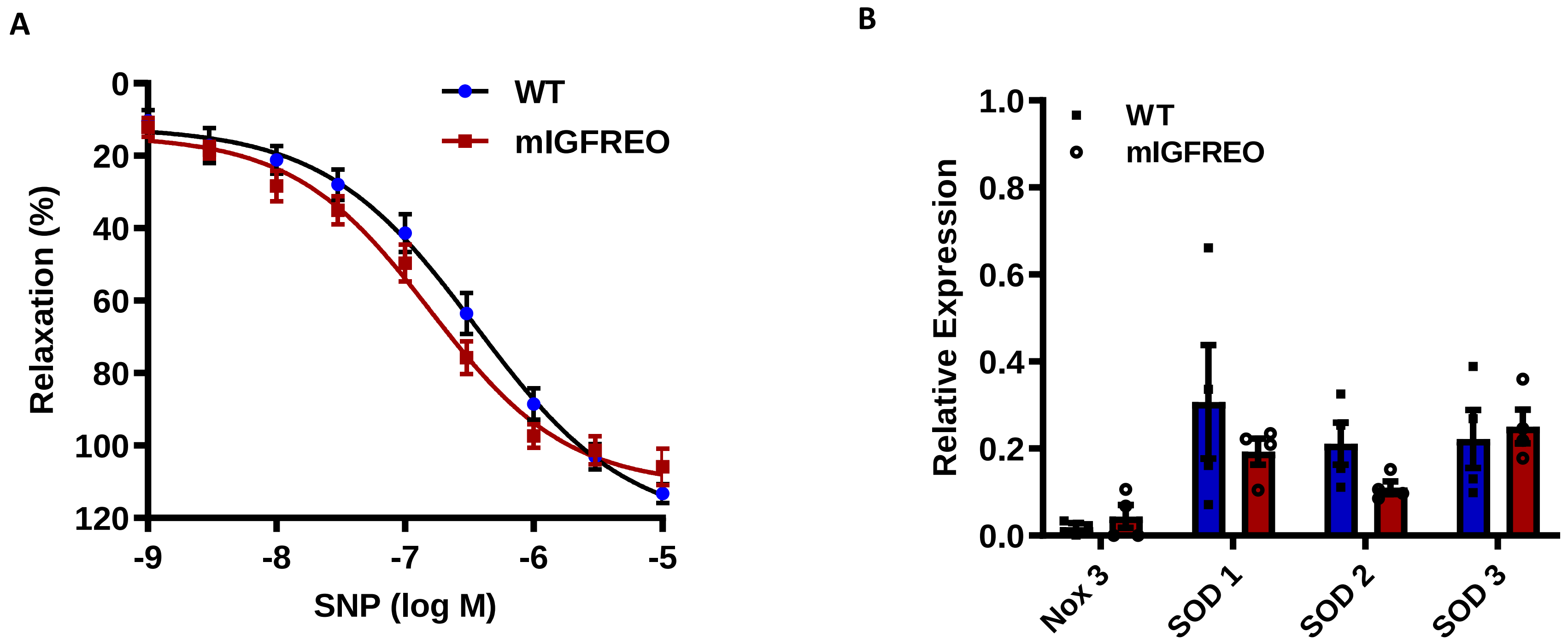

Online Figure VII

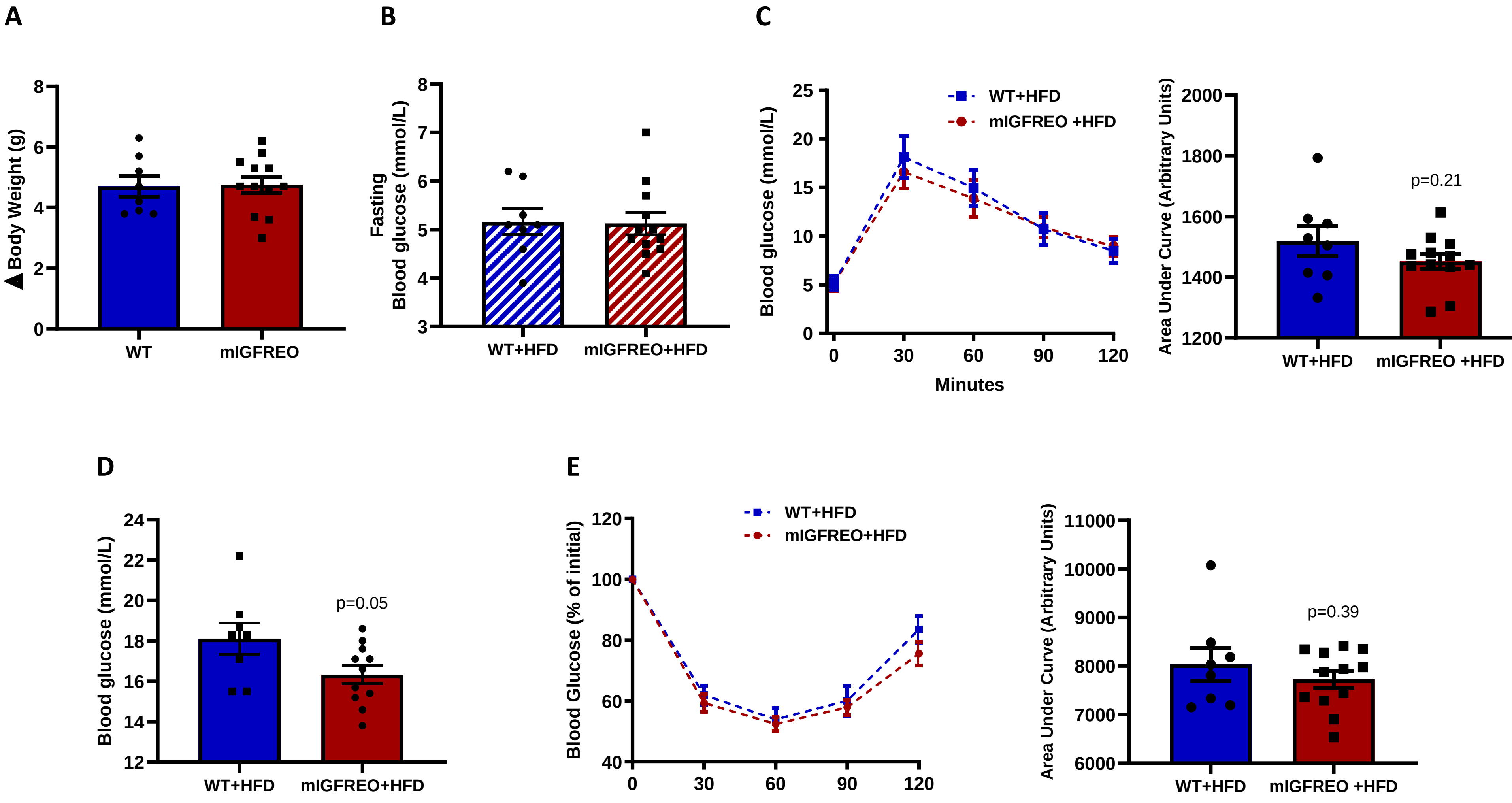


GTT: Blood glucose at 30 minutes
